# Targeted CUL4A inhibition synergizes with cisplatin to yield long-term survival in models of head and neck squamous cell carcinoma through a DDB2-mediated mechanism

**DOI:** 10.1038/s41419-022-04798-6

**Published:** 2022-04-15

**Authors:** Trace M. Jones, Claudia M. Espitia, Aikseng Ooi, Julie E. Bauman, Jennifer S. Carew, Steffan T. Nawrocki

**Affiliations:** 1 University of Arizona Cancer Center, Tucson, AZ USA; 2 Department of Pharmacology and Toxicology, University of Arizona, Tucson, AZ USA

**Keywords:** Preclinical research, Translational research, Cancer therapeutic resistance, Oral cancer

## Abstract

Patients with late-stage and human papillomavirus (HPV)-negative head and neck squamous cell carcinoma (HNSCC) continue to have a very poor prognosis. The development of more effective novel therapies that improve overall survival and overcome drug resistance is an urgent priority. Here we report that HNSCC tumors significantly overexpress NEDD8 and exhibit high sensitivity to the first-in-class NEDD8-activating enzyme (NAE) inhibitor pevonedistat. Additional studies established that disruption of NEDD8-mediated protein turnover with pevonedistat dramatically augmented cisplatin-induced DNA damage and apoptosis in HNSCC models. Further analysis revealed that the specific pevonedistat target CUL4A played an essential role in driving the synergy of the pevonedistat and cisplatin combination. Targeted inhibition of CUL4A resulted in significant downregulation in Damage Specific DNA binding protein 2 (DDB2), a DNA-damage recognition protein that promotes nucleotide excision repair and resistance to cisplatin. Silencing of CUL4A or DDB2 enhanced cisplatin-induced DNA damage and apoptosis in a manner similar to that of pevonedistat demonstrating that targeted inhibition of CUL4A may be a novel approach to augment cisplatin therapy. Administration of pevonedistat to mice bearing HNSCC tumors significantly decreased DDB2 expression in tumor cells, increased DNA damage and potently enhanced the activity of cisplatin to yield tumor regression and long-term survival of all animals. Our findings provide strong rationale for clinical investigation of CUL4A inhibition with pevonedistat as a novel strategy to augment the efficacy of cisplatin therapy for patients with HNSCC and identify loss of DDB2 as a key pharmacodynamic mediator controlling sensitivity to this regimen.

## Introduction

Head and neck cancer is a general term for malignancies of the upper aerodigestive tract. The vast majority of these tumors originate in the epithelial mucosal lining and are classified as squamous cell carcinomas [1]. Approximately 900,000 new cases of head and neck squamous cell carcinoma (HNSCC) are diagnosed worldwide each year. More than 80% are attributed to environmental carcinogenesis associated with tobacco, areca nut, and alcohol use and the minority to viral carcinogenesis due to human papillomavirus (HPV) or Epstein Barr virus [2]. While patients presenting with early stage, HPV-negative disease have a favorable prognosis, most patients present with locally advanced disease and have limited overall survival despite treatment intensification [3]. Despite the U.S. FDA approval of anti-EGFR targeted therapeutics and anti-PD1 immunotherapies over the past two decades, relative survival rates of HPV-negative HNSCC patients remains virtually unchanged. This underscores an urgent and critical need to develop therapeutics that target novel pathways.

The NEDDylation pathway controls the degradation of a subset of proteins that regulate DNA damage repair, cell cycle progression, and apoptosis [4]. The NEDDylation process plays a pivotal role in maintaining cellular homeostasis and is highly regulated by multiple enzymes that mediate the conjugation of a ubiquitin-like molecule, NEDD8, onto specific substrates to modulate their function. The cullin-RING Ligases (CRLs) are a family of E3-ubiquitin ligases whose activity is modulated by NEDD8 conjugation [5]. CRLs are responsible for the ubiquitination and subsequent degradation of hundreds of targets. Overexpression of the various CRLs or upstream enzymes involved in NEDDylation have been observed in multiple tumor types, including HNSCC [6–8]. Hyperactivation of this pathway is associated with a poor prognosis in many cancer types, thus providing a strong rationale for targeting NEDDylation in tumors that depend on this pathway for survival [6]. Pevonedistat is a first-in-class highly selective inhibitor of NEDD8-Activating Enzyme (NAE), the proximal enzyme in the NEDDylation cascade. Pevonedistat is a small molecule adenosine mimetic that forms an irreversible covalent adduct in the ATP-binding pocket of NAE, thus blocking NEDDylation and the downstream degradation of CRL target proteins [9]. Early clinical trials of pevonedistat have demonstrated significant therapeutic activity and favorable safety indicating that further investigation of this agent is warranted [10–12].

The DNA crosslinking agent cisplatin is currently the primary therapeutic used in both the curative and palliative management of HNSCC patients [13]. While many patients experience an initial remission when exposed to curative intent, platinum-based multimodality therapy, a significant proportion relapse within two years of therapy [14]. Similarly, platinum-based chemotherapy plus the anti-EGFR monoclonal antibody cetuximab or the anti-PD1 mAb pembrolizumab drives a high initial response rate in recurrent/metastatic disease, however resistance universally emerges. One established mechanism of resistance to cisplatin is the upregulation of DNA damage repair pathways, in particular nucleotide excision repair (NER), which is responsible for the elimination of the majority of cisplatin lesions thereby reducing its efficacy [15–18]. Interestingly, NER is initiated by a set of CRLs, CUL4A, and CUL4B [19]. Given this, NEDDylation may play an important role in the NER pathway. However, this has not been thoroughly investigated.

Here, we show that pevonedistat significantly augments the DNA damaging effects of cisplatin against HNSCC cells. Mechanistically, this is driven through inhibition of CUL4A, which causes a significant downregulation of DDB2, the primary DNA-damage recognition protein in NER. Importantly, the pevonedistat and cisplatin combination synergistically decreased tumor burden and yielded long-term animal survival in a HNSCC xenograft study. Our collective findings establish the rationale for clinical testing of this combination in patients with HNSCC.

## Materials and methods

### Cell lines and cell culture

The HPV-negative HNSCC cell lines FaDu, Cal27, A253, and Detroit 562 were obtained from American Type Culture Collection (ATCC, Manassas, VA) and were mycoplasma free. Cell lines were authenticated using short tandem repeat DNA profiling techniques. Cells were cultured in RPMI-1640 medium supplemented with 10% FBS at 37 °C in 5% carbon dioxide. Harvesting of cells was performed by washing cells in PBS and then incubating cells at 37 °C with a 0.25% trypsin, 2.21 mM EDTA, and 1X sodium bicarbonate solution. Cells were counted using a Beckman Coulter Vi-CELL XR Cell Viability Analyzer (Beckman-Coulter, Brea, CA).

### Chemicals and reagents

Reagents were obtained from the following sources: Pevonedistat (#S7109) was purchased from SelleckChem (Houston, TX), and cisplatin was obtained from the hospital pharmacy. The antibodies anti-NEDD8 (ab81264), anti-p21 (ab109520), anti-p27 (ab32034), anti-γH2AX (Ser-139, ab81299), anti-CUL4A (ab92554), anti-CUL4B (ab67035), anti-phospho-Rb (Ser-780, ab173289), anti-Rb (ab181616), anti-DDB2 (ab181136), anti-E2F1 (ab179445), and anti-cisplatin-DNA adduct (ab103261) were purchased from Abcam (Cambridge, MA). Anti-NAE (CST14321), anti-WEE1 (CST4936), anti-active caspase-3 (CST9661), and anti-β-actin (CST3700) were obtained from Cell Signaling Technology (Danvers, MA). Anti-β-Tubulin (MA5-16308), goat anti-rat Alexa-Fluor 488 (#11006), goat anti-rabbit Alexa-Fluor 554 (#11010), goat anti-mouse Alexa-Fluor 488 (#11001), Prolong Gold antifade with DAPI (#P36931) and SYBR-Gold (#511494) were purchased from Thermo Fisher (Waltham, MA). Goat Anti-Rabbit HRP tagged secondary antibody (#111-035-144) was obtained from Jackson ImmunoResearch Laboratories (West Grove, PA).

### NEDD8 expression in clinical samples

The HTSeq generated total number of mapped reads per gene count data for 500 HNSCC tumor and 44 normal samples were downloaded from the GDC data portal on 31 July 2020. The data were analyzed using the DESeq2 package in the R statistical environment [20]. Variance stabilized transformed data were used for graphing and visualization.

### Cell viability assays

3-(4,5-dimethylthiazol-2-yl)-2,5-diphenyltetrazolium bromide (MTT, #M5655, Sigma, St. Louis, MO) viability assay was performed in eight replicates. Cells were incubated in the specified concentration of drug for 72 h. In all, 50 μL of 4 mg/ml MTT reagent was added to each well and cells were incubated for 2 h at 37 °C. Formazan precipitate was solubilized in DMSO and absorbances were quantified at 570 nm using a SpectraMax plate reader (Molecular Devices, San Jose, CA).

Colony assays were performed in triplicate. Cells were plated and incubated for 24 h with the appropriate drug concentration. Drugs were washed away and cells were incubated in fresh media for 10 days. Cells were then fixed at room temperature with 3:1 methanol-acetic acid solution for five minutes and stained for five minutes in a 0.5% crystal violet in methanol solution. Colonies were manually counted.

### Immunoblotting

Cells were incubated for 24 or 48 h in indicated drug concentrations. Cells were harvested and lysed and proteins were separated by SDS-PAGE and transferred to a nitrocellulose membrane. Membranes were blocked at room temperature for one hour in 5% BSA in TBST. Membranes were incubated overnight at 4 °C with the indicated primary antibody. Fluorescent secondary antibodies were added for one hour at room temperature. Bands were detected using a fluorescent imager (LI-COR, Lincoln, NE). β-actin and β-tubulin were used to demonstrate equal protein loading. Quantification of blots was carried out using ImageStudio densitometry software.

### Oxidative DNA damage assay

FaDu cells were treated with 300 nM pevonedistat for 24 h. Cells were washed with PBS, trypsinized, and stained with a FITC-tagged 8-oxoguanine antibody (ab183393). Wash and fixation buffers (BD550480) were purchased from BD BioSciences (San Jose, CA). Stained cells were analyzed using a BD FACSCelesta flow cytometer. Average FITC signal intensity was measured for each sample. Experiments were performed in triplicate.

### Quantitative RT-PCR

Cells were treated with the appropriate drug and concentration for 24 h and then were collected. RNA was isolated using RNeasy mini kit (#74134, Qiagen, Germantown, MD) according to the manufacturer’s protocol. cDNA was produced according to the manufacturer’s (ThermoFisher, Waltham, MA) instructions using the high-capacity cDNA reverse transcription kit (#4374966). Taqman primers for *DDB2* (#4331182) and *GAPDH* (#4331182) were obtained from ThermoFisher. All experiments were performed in triplicate.

### Apoptosis assays

For active caspase-3 assay, cells were incubated for 24–48 h with the indicated drug concentrations. Cells were washed with PBS, trypsinized, and stained for active caspase-3 following the protocol using the FITC Active Caspase-3 Apoptosis Kit (BD550480) according to the manufacturer’s instructions (BD BioSciences). Quantification of FITC fluorescence intensity was performed on a BD FACSCelesta flow cytometer. All experiments were performed in triplicate.

Propidium Iodide (PI) staining was performed in triplicate. Cells were incubated for 24–72 h in specified drug concentration. Cells were stained with a 25 μg/mL PI, 0.1% TritonX-100, 0.1% sodium citrate solution for four hours at 4 °C. DNA content of cells was analyzed using a BD FACSCelesta flow cytometer (BD Biosciences).

### Alkaline comet assay

Cells were treated with pevonedistat, cisplatin, or a combination of both for 24 h. The comet assay was performed using the Comet Assay kit (#4250-050-K, R&D Systems, Minneapolis, MN) following manufacturer’s instructions. Briefly, test samples were mixed with low melting point agarose gel, spread onto chamber slides, and allowed to cool. Slides were immersed in Lysis Solution and incubated at 4 °C for one hour. Slides were incubated for 20 min at room temperature (RT) in Alkaline Unwinding solution (200 mM NaOH, 1 mM EDTA). Slides were submersed in Alkaline Electrophoresis Solution (200 mM NaOH, 1 mM EDTA) inside the electrophoresis chamber, and subjected to 21 volts for one hour. Slides were washed twice, for five minutes, with water. Slides were washed for five minutes in 70% ethanol. Slides were allowed to dry at 37 °C for 15 min. Samples were stained with SYBR Gold in TE buffer, pH 7.5 for 30 min at RT. Cells were imaged using a Zeiss Axio Vert.A1 microscope. Tail Moments (product of amount of DNA in tail and tail length) were calculated using Open Comet software. Five images, spanning each section of the slide, were used to quantify tail moments for each condition.

### Immunocytochemistry

Cells were plated in two-well chamber slides and treated with the appropriate concentration of pevonedistat, cisplatin, or the combination for 18–24 h. Cells were washed with PBS, fixed in 4% formaldehyde or 70% ethanol (anti-cisplatin-DNA adduct) for 15 min at RT, permeabilized with 0.2% TritonX-100 for 10 min at RT, and incubated overnight at 4 °C with a γH2AX or cisplatin-DNA adduct primary antibody. Goat anti-mouse Alexa-Fluor-488, goat anti-mouse Alexa-Fluor-544, or goal anti-rat Alexa-Fluor-488 secondary antibodies were used to visualize γH2AX and cisplatin-DNA adducts. DAPI was used as a nuclear counterstain. Images were taken using a Zeiss Axio Vert.A1 microscope. ImageJ software was used to calculate the average signal intensity for each condition.

### shRNA silencing of *CUL4A*, *CUL4B*, *DDB2*, and *E2F1*

FaDu cells were infected with lentiviral particles containing non-targeted (control) or target-specific short hairpin RNA (shRNA) directed at *CUL4A* (sc-44355-V), *CUL4B* (sc-37572-V), *DDB2* (sc-37799-V), and *E2F1* (sc-29297-V) according to the manufacturer’s protocol (Santa Cruz Biotechnology, Santa Cruz, CA). Successful transfection was selected for with puromycin. Transfected cells were treated with the indicated drugs and concentrations. Apoptosis and DNA damage signaling were assessed by PI-FACS and immunocytochemistry analyses. Knockdown efficiency was determined by immunoblotting.

### DDB2 reporter assay

FaDu cells were transfected with lentiviral particles containing a Gaussia luciferase gene regulated by a *DDB2* (LPP-HPRM30091-LvPG02), *GAPDH* (LPP-GAPDH-LVPG02), or negative control (LPP-NEG-LVPG02) promoter (GeneCopoeia, Rockville, MD). Each lentiviral construct contained a puromycin resistance gene to select successfully transfected populations. Transfected cells were incubated with the stated concentration of pevonedistat for 24 h. Luciferase expression was quantified using a Secrete Pair Gaussia Luciferase Assay kit (#LF062, GeneCopoeia, Rockville, MD) according to the manufacturer’s protocol. Luminescence measurements were obtained using a Molecular Devices SpectraMax plate reader. Experiments were performed in triplicate.

### Overexpression of CUL4A and DDB2

FaDu cells were infected with lentiviral particles containing a puromycin resistance gene, a *CUL4A* (#RC214798L4V) cDNA construct, or a *DDB2* (#RC200390L4V) cDNA construct according to the manufacturer’s protocol (Origene, Rockville, MD). Successful transfection was selected for with puromycin. Cell viability and DNA damage was assessed as described above. Overexpression efficiency was confirmed by immunoblotting.

### In vivo evaluation

All in vivo studies were conducted to conform to the guidelines in the “Guide for Care and Use of Laboratory Animals” under an approved institutional animal care and use committee (IACUC) protocol at the University of Arizona. FaDu cells were trypsinized, collected, washed in PBS, and suspended in a 1:1 mixture of Hanks balanced salt solution (HBSS) and Matrigel (#354234, Corning). Cells were injected subcutaneously into the flanks of 8-week old female nude (nu/nu) mice (Taconic, Rensselaer, NY). Tumors were grown to a starting volume of 150 mm^3^. Mice were then randomly assigned into treatment groups (*N* = 10 per group) to receive vehicle control, pevonedistat 60 mg/kg SC five times a week, cisplatin 3 mg/kg IP twice a week, or both pevonedistat and cisplatin for approximately three weeks. Tumor volume and mouse weight were measured twice weekly. At the cessation of treatment, representative tumors were excised, formalin-fixed, and paraffin embedded for pharmacodynamic analyses. Mice were monitored twice weekly for 100 days to establish long-term survival. Animal studies were conducted in an unblinded fashion to assure that mice in each group received the appropriate treatments.

### Immunohistochemistry

Paraffin-embedded tumor sections were deparaffinized in xylene, immersed in a graded series of ethanol, and rehydrated in pH 7.5 PBS. Sections were microwaved in citrate buffer for five minutes to retrieve epitopes. Endogenous peroxide blocking was performed by incubation of samples in 3% H_2_O_2_ in methanol for 10 min. Samples were incubated in blocking solution (5% horse and 1% goat serum in PBS) for 20 min. Primary antibodies targeting γH2AX, active caspase-3, DDB2, phospho-Rb, Rb, and p21 were diluted in blocking solution. Samples were incubated with the appropriate primary antibody overnight at 4 °C. After washing with PBS, samples were incubated with the appropriate secondary antibody for 90 min at RT. After washing with PBS, samples were then incubated with 3,3′-diaminobenzidine (#K3468, Dako, Santa Clara, CA) for 30 min at RT. Slides were rinsed with water and counterstained for one minute with Gill’s hematoxylin (#GHS132, Sigma). Images were captured on Zeiss Axio Vert.A1 microscope. ImageJ software with a Fiji plugin was used to quantify staining intensity.

### Synergy analysis

The combination indices (CI) for pevonedistat and cisplatin were calculated using a 72-hour MTT assay. CompuSyn software (Combosyn, Inc., Paramus, NJ) was utilized to calculate CI values.

### Statistical analyses

Statistical significance of differences among samples was determined using the Student’s *t* test, one-way ANOVA, and a Kaplan-Meier analysis where appropriate. Differences were considered significant at *p* < 0.05. For TCGA data analysis, HNSCC RNAseq data were downloaded from the National Cancer Institute, GDC data portal in HTSeq-Counts format. Differentially expressed genes were identified using a negative binomial test method implemented in the DESeq 2 package [20].

## Results

### Inhibition of NEDDylation stabilizes CRL target proteins and reduces HNSCC cell viability

**Fig. 1:**
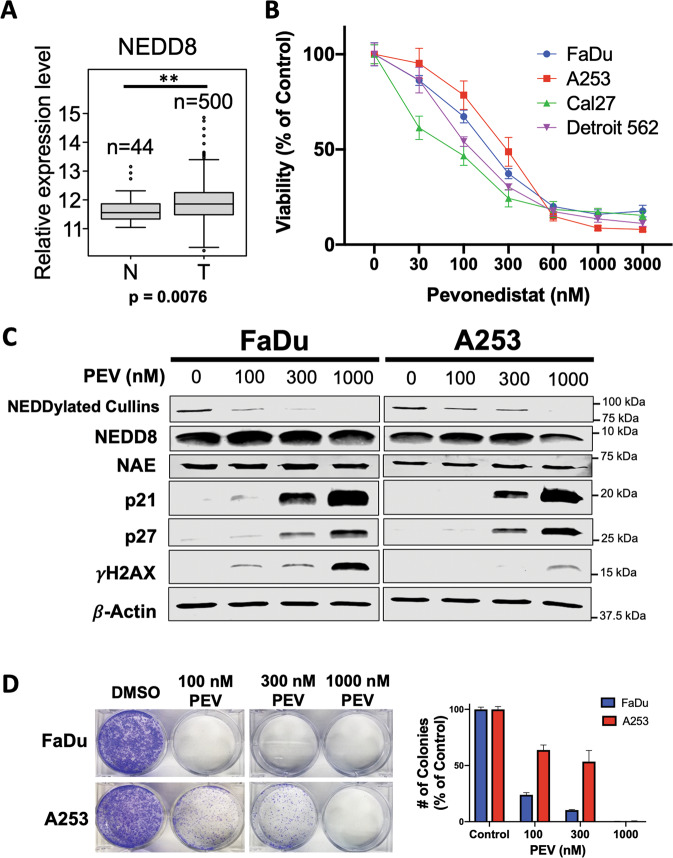
The NEDD8 pathway is a rational target for HNSCC therapy. **A** NEDD8 is significantly elevated in clinical cases of HNSCC. Gene expression data from the GDC data portal were analyzed for levels of NEDD8 in 500 HNSCC tumor (T) and 44 adjacent normal (N) tissue samples. Mean ± SD. **Indicates a significant difference at *p* = 0.0076. **B** Pevonedistat decreases HNSCC cell viability in a dose-dependent manner. FaDu, A253, Cal27, and Detroit-562 cell lines were treated with varying concentrations of pevonedistat for 72 h. Viability was assessed by MTT assay. Mean ± SD, *n* = 8. **C** Pevonedistat stabilizes CRL targets in HNSCC cell lines. FaDu and A253 HNSCC cells were treated with the indicated concentrations of pevonedistat for 24 h. NEDD8, NAE, p21, p27, and γH2AX were measured by immunoblotting. β-Actin was used as a loading control. **D** Pevonedistat inhibits colony formation in HNSCC cell lines. FaDu and A253 cell lines were plated and treated with various concentrations of pevonedistat for 24 h. The cells were then incubated in fresh media for 10 days and colonies were quantified. Mean ± SD, *n* = 3.

To establish the clinical status of the NEDDylation cascade in HNSCC, we first determined NEDD8 levels in normal (*n* = 44) and HNSCC (*n* = 500) tissue using the TCGA database (Fig. 1A). Head and neck tumors were found to significantly overexpress NEDD8 when compared to normal adjacent tissue (*p* = 0.0076), thus establishing the rationale for exploring targeted inhibition of NEDDylation as an approach for HNSCC therapy. Accordingly, we quantified the effect of pevonedistat (dose range 30-3000 nM) on the viability of HPV-negative HNSCC cell lines originating from a variety of anatomical sites (FaDu, A253, Cal27, Detroit 562). Pevonedistat reduced the viability of HNSCC cells in a dose-dependent manner (Fig. 1B). Notably, all cell lines exhibited an IC_50_ value in the sub-micromolar range. To better understand the protein-directed pharmacodynamics (PD) of pevonedistat, we conducted immunoblotting for CRL target proteins as well as the functional targets of pevonedistat (Fig. 1C). As we and others have previously demonstrated, pevonedistat treatment decreased levels of NEDDylated cullins without impacting levels of free NEDD8 or NAE and stabilized established CRL targets, p21 and p27, in a dose-dependent manner [21–26]. Treatment with pevonedistat also increased expression of the DNA damage marker γH2AX. To further assess the DNA-damaging activity of pevonedistat, we stained treated cells for 8-oxoguanine, a well-established indicator of oxidative DNA-damage (Supplementary Fig. S1). Consistent with previous findings in other models, FaDu cells treated with 300 nM of pevonedistat displayed elevated levels of 8-oxoguanine, further confirming that pevonedistat induces DNA damage as a single agent [27]. We then analyzed the ability of pevonedistat to disrupt colony formation. 24 h exposure with various concentrations (100–1000 nM) of pevonedistat significantly decreased the clonogenic potential of HNSCC cells (Fig. 1D).

### Pevonedistat significantly increases the DNA damaging and anticancer activity of cisplatin in vitro

**Fig. 2:**
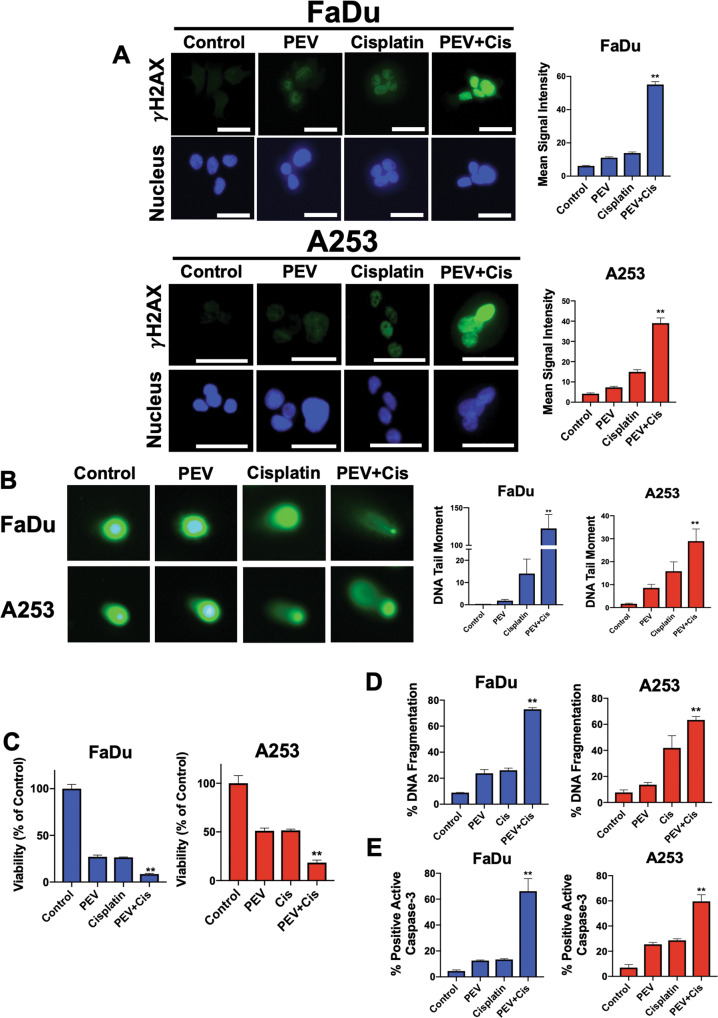
Pevonedistat significantly augments cisplatin-mediated DNA damage. **A** Pevonedistat and cisplatin treatment significantly increase γH2AX expression. FaDu and A253 cells were treated with 600 nM pevonedistat, 3 μM cisplatin, or the combination for 24 h. γH2AX levels were measured by immunocytochemistry and quantified using ImageJ software. Representative images are shown. DAPI was used as a counterstain. Mean signal intensity ± SEM, *n* = 54 cells per condition. Scale bar = 20 μm. **B** Pevonedistat significantly augments cisplatin-induced DNA damage. Cells were treated with 600 nM pevonedistat, 3 μM cisplatin, or the combination for 24 h. DNA damage was determined by comet assay and DNA tail moment was determined. Mean ± SEM, *n* = 27. **C** Pevonedistat significantly enhances cisplatin-mediated reductions in cell viability. FaDu and A253 cells were treated with 0.3 μM pevonedistat, 1–3 μM cisplatin, or the combination for 72 h. Cell viability was determined by MTT assay. Mean ± SD, *n* = 4. **D**, **E** Pevonedistat significantly augments cisplatin-induced apoptosis. FaDu and A253 cells were treated with 1 μM pevonedistat, 10 μM cisplatin, or the combination for 24 h. Apoptosis was measured by PI-FACS analysis (**D**) and active caspase-3 assay (**E**). Mean ± SD, *n* = 3. **Indicates a significant difference from either monotherapy, *p* < 0.05.

Based on the observed DNA damaging effects of pevonedistat, we hypothesized that it may significantly augment the efficacy of cisplatin. Indeed, co-treatment with pevonedistat and cisplatin dramatically increased the intracellular levels of γH2AX as quantified by immunocytochemistry (Fig. 2A). Immunoblotting assays confirmed these results (Supplementary Fig. S2). To further investigate the DNA-directed effects of this combination, we performed alkaline comet assays to detect the presence of physical DNA double strand breaks (DSBs) (Fig. 2B). Consistent with the elevated levels of γH2AX induced by this combination, comet assays revealed a significant increase in DNA DSBs in HNSCC cells. Finally, we used immunocytochemistry to quantify cisplatin-DNA adducts (Supplementary Fig. S3 and Supplementary Table S1). We detected significant levels of cisplatin-DNA adducts following single agent cisplatin treatment. Importantly, co-treatment with pevonedistat significantly increased both cisplatin-DNA adduct signal intensity (Supplementary Figure S3) and the proportion of cells positive for the lesions (Supplementary Table S1) over what was achieved with cisplatin monotherapy. This demonstrated that the increased levels of DNA damage induced by combination treatment are linked to the ability of pevonedistat to enhance the formation of cisplatin-DNA adducts. We next investigated whether the synergistic effects of pevonedistat and cisplatin with respect to DNA damage would translate into synergistic anticancer activity. Cells were treated with pevonedistat, cisplatin, or both drugs to determine the combination indices (CI, Fig. 2C). Formal synergy analyses showed that the combination of pevonedistat and cisplatin yielded CI values less than one, indicating true synergy (Supplementary Table S2). We confirmed this finding by quantifying DNA fragmentation by PI-FACS analysis in FaDu and A253 cells (Fig. 2D and Supplementary Fig. S4). While each single agent displayed significant pro-apoptotic effects, the combination of pevonedistat and cisplatin showed a marked improvement in the induction of DNA fragmentation. In agreement with this data, we also observed a significant increase in active caspase-3 levels following combination treatment (Fig. 2E). Taken together, these data demonstrate that pevonedistat and cisplatin synergistically induce DNA damage-mediated apoptosis in HNSCC models.

### Knockdown of CUL4A, but not CUL4B, significantly sensitizes HNSCC cells to cisplatin

**Fig. 3:**
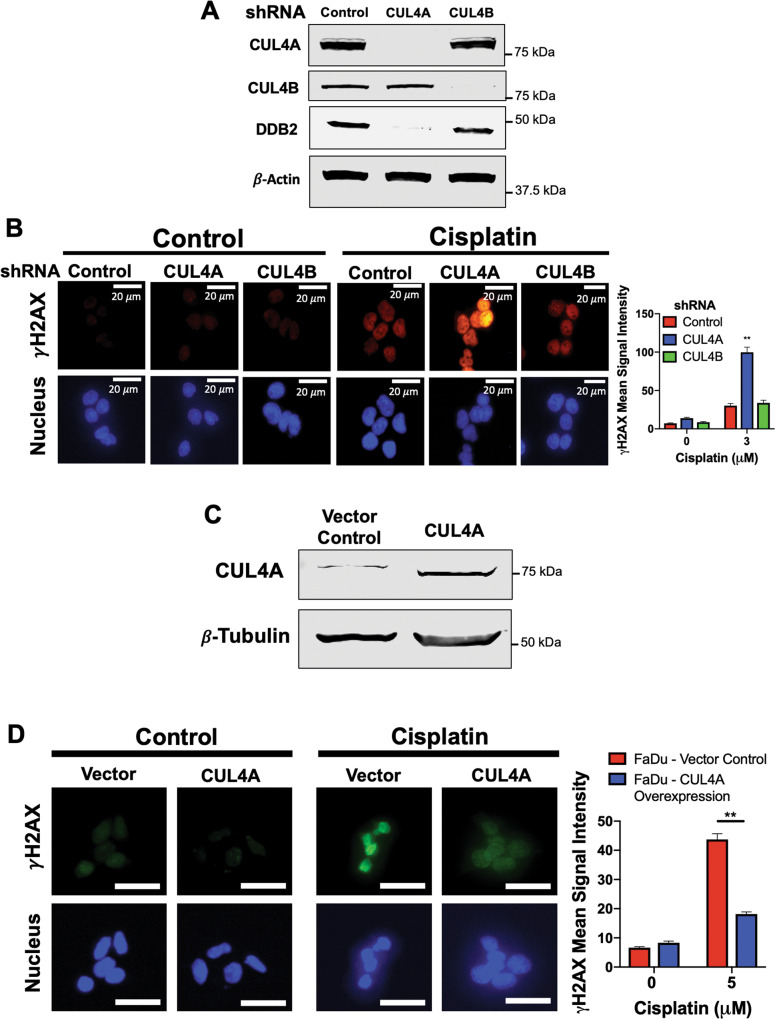
CUL4A regulates cisplatin-induced DNA damage. **A** Lentiviral shRNA knockdown of CUL4A decreases DDB2 expression. FaDu cells were transfected with lentiviral shRNA particles targeting CUL4A, CUL4B or a scramble control. The levels of CUL4A, CUL4B, and DDB2 were determined by immunoblotting. **B** Knockdown of CUL4A leads to increased γH2AX expression. FaDu cells were transfected with lentiviral shRNA targeting CUL4A, CUL4B, or a scramble control. Cells were then treated with 3 μM cisplatin for 24 h. γH2AX levels were determined and quantified by ImageJ software. DAPI was used as a counterstain. Representative images are shown. Mean signal intensity ±SEM, *n* = 20 cells per condition. **Indicates a significant difference from other treated groups, *p* < 0.01. **C** FaDu cells were transfected with lentiviral particles containing a functional CUL4A expression vector. Overexpression of CUL4A was confirmed by immunoblotting following puromycin selection. **D** Overexpression of CUL4A decreases cisplatin-mediated DNA damage. FaDu cells transfected with CUL4A overexpression and control plasmid were incubated with 5 μM cisplatin for 24 h. γH2AX expression was observed and quantified using ImageJ software. DAPI was used as a nuclear counterstain. Representative images are shown. Mean signal intensity ±SEM, *n* = 66 cells per condition. Scale bar = 20 μm. **Indicates a significant difference between samples, *p* < 0.01.

We next sought to elucidate the specific downstream CRL target of pevonedistat driving the amplified DNA damaging effects of the pevonedistat and cisplatin combination. Of the eight CRLs present in mammalian cells, CUL4A and CUL4B are the most intrinsically linked to DNA repair, as they have been shown to play an initiatory role in the NER pathway [19]. CUL4 proteins bind to a DDB1 and DDB2 heterodimer and the resulting complex is capable of recognizing bulky adducts on the DNA strand [28]. To genetically mimic their inhibition by pevonedistat, we silenced CUL4A and CUL4B expression in FaDu cells (Fig. 3A). Interestingly, DDB2 was shown to be highly downregulated in response to knockdown of CUL4A, but not CUL4B. DDB2 acts as an adaptor in the damage recognition complex and interacts with damaged DNA [29].

To assess the effect of CUL4A and CUL4B silencing on cisplatin-induced DNA damage, we performed immunocytochemistry for γH2AX (Fig. 3B). In response to cisplatin treatment, the vector control and CUL4B shRNA cells displayed γH2AX levels similar to those seen in single-agent cisplatin-treated cells. However, knockdown of CUL4A resulted in a significantly higher induction of γH2AX following cisplatin treatment. To further investigate the role of CUL4A and its relationship to cisplatin sensitivity, we overexpressed CUL4A in FaDu cells (Fig. 3C). We exposed FaDu cells overexpressing CUL4A to slightly higher levels of cisplatin due to the emergence of drug resistance. Overexpression of CUL4A resulted in significantly lower levels of γH2AX (Fig. 3D) and decreased sensitivity to cisplatin-induced apoptosis (Supplementary Fig. S5). Collectively, these data demonstrate that inhibition of CUL4A (and not CUL4B) significantly contributes to the DNA damage-induced apoptosis triggered by pevonedistat and cisplatin combination therapy.

### Pevonedistat-mediated downregulation of DDB2 increases sensitivity to cisplatin

**Fig. 4:**
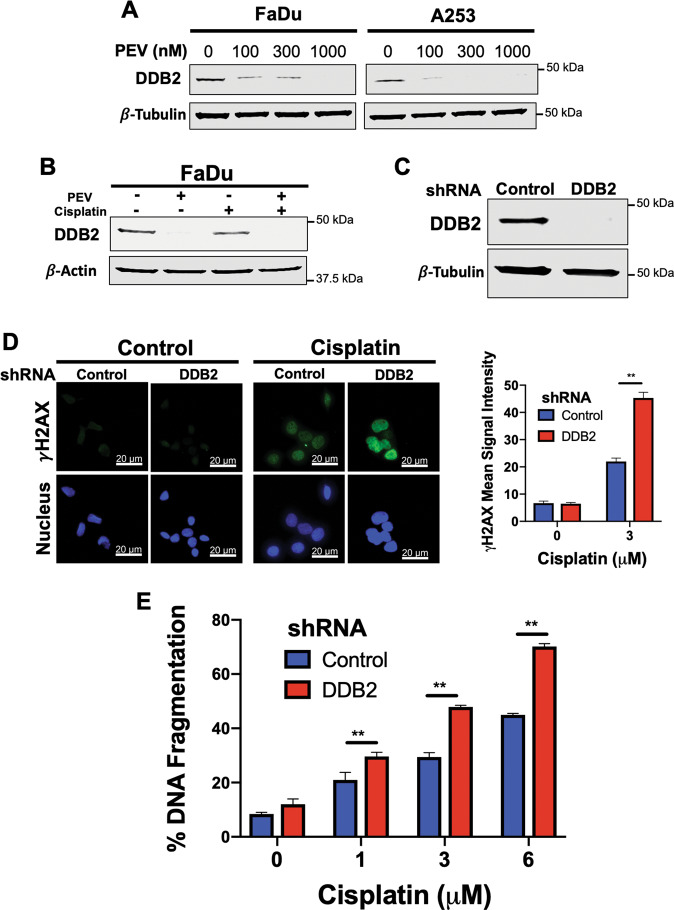
Pevonedistat-mediated downregulation of DDB2 drives its synergy with cisplatin. **A**, **B** Pevonedistat decreases the expression of DDB2. FaDu and A253 cells were treated with the indicated concentrations of pevonedistat (**A**) and were treated with 600 nM pevonedistat, 3 μM cisplatin, and the combination (**B**) for 24 h. DDB2 expression was determined by immunoblotting. **C** DDB2 was silenced in FaDu cells using lentiviral shRNA. Cells were infected with DDB2 and scramble control shRNA and placed under puromycin selection. DDB2 levels were determined by immunoblotting. **D** Knockdown of DDB2 increases γH2AX levels following cisplatin treatment. FaDu cells transfected with lentiviral shDDB2 or control particles were treated with 3 μM cisplatin for 24 h. γH2AX signal was observed by fluorescent imaging and quantified using ImageJ software. DAPI was used as a counterstain. Mean signal intensity ±SEM, *n* = 58 cells per condition. **E** Knockdown of DDB2 increases cisplatin-induced apoptosis. FaDu cells transfected with lentiviral DDB2 or control shRNA were treated with the indicated concentrations of cisplatin for 48 h. Apoptosis was determined by PI-FACS analysis. Mean ± SD, *n* = 3. **Indicates a significant difference between indicated groups, *p* < 0.01.

Our data showed that genetic knockdown of CUL4A resulted in the downregulation of DDB2. We next assessed whether pharmacological inhibition of CUL4A with pevonedistat could recapitulate these effects. Immunoblotting assays demonstrated pevonedistat treatment decreased DDB2 expression in a dose-dependent manner (Fig. 4A). Diminishment of DDB2 levels by pevonedistat also occurred when given in combination with cisplatin (Fig. 4B). We subsequently sought to further understand the significance of DDB2 downregulation as a regulator of pevonedistat sensitivity. Using lentiviral shRNA, we silenced DDB2 in FaDu cells (Fig. 4C) and evaluated γH2AX expression in cells with differing DDB2 levels following cisplatin treatment. Knockdown of DDB2 yielded a significantly higher mean signal intensity of γH2AX when compared to cells transfected with vector control (Fig. 4D). Treatment of control and DDB2 shRNA cells with cisplatin showed that DDB2 knockdown significantly enhanced cisplatin-mediated apoptosis (Fig. 4E). Next, we forced DDB2 overexpression in FaDu cells in the context of pevonedistat treatment to determine whether this would be sufficient to diminish the synergistic activity of the combination (Supplementary Fig. S6A). At a moderate concentration of pevonedistat (300 nM), DDB2 overexpression significantly blunted the anticancer effects of a variety of concentrations of cisplatin (Supplementary Fig. S6B). Using immunocytochemistry, we confirmed that cells overexpressing DDB2 displayed lower levels of γH2AX in response to cisplatin and combination therapy (Supplementary Fig. S7A). Importantly, we also demonstrated that GFP-tagged DDB2 co-localized with γH2AX (Supplementary Fig. S7B). Collectively, these data demonstrate that DDB2 plays an important role in cisplatin sensitivity and that genetic or pharmacological inhibition of CUL4A sensitizes HNSCC cells to cisplatin-induced DNA damage and apoptosis.

### DDB2 is transcriptionally downregulated through decreased activity of E2F1

**Fig. 5:**
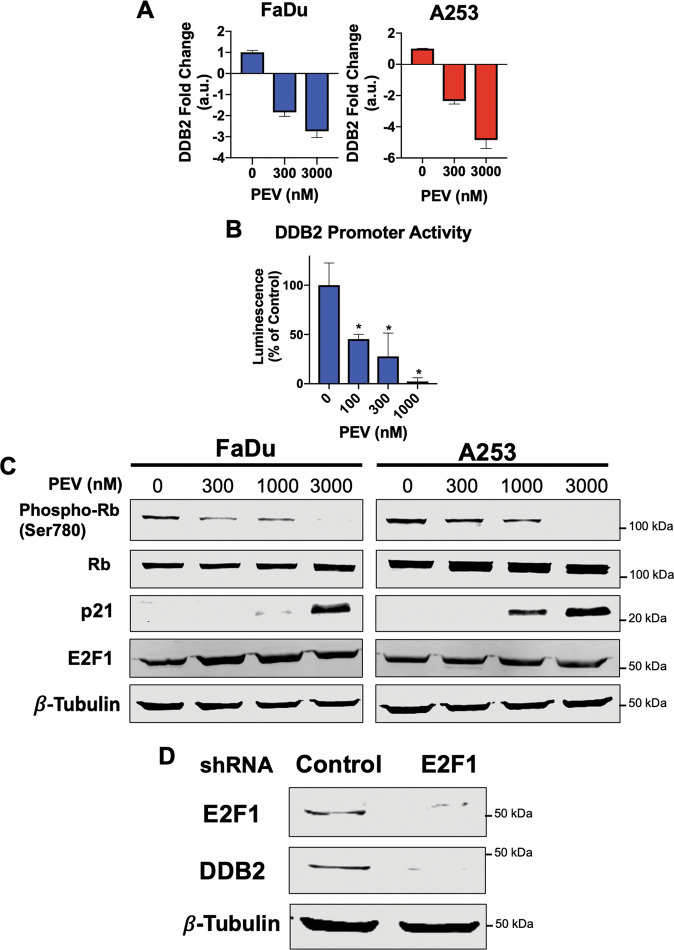
Inhibition of E2F1 underlies pevonedistat-driven suppression of DDB2 levels. **A** Pevonedistat treatment results in the transcriptional downregulation of DDB2. FaDu and A253 cells were treated with the indicated concentrations of pevonedistat for 24 h. qRT-PCR was used to quantify changes in DDB2 expression. Mean ± SD, *n* = 3. **B** Pevonedistat decreases DDB2 promoter activity. FaDu cells were transfected with lentiviral particles containing a DDB2 promoter flanking a luciferase gene. Cells stably expressing this construct were treated with the indicated concentrations of pevonedistat for 24 h and DDB2 promoter activity was quantified. Mean ± SD, *n* = 3. *Indicates a significant difference from control, *p* < 0.05. **C** Pevonedistat increases p21 expression, which is associated with a reduction in Rb phosphorylation. FaDu and A253 cells were treated with the indicated concentrations of pevonedistat for 24 h. Phospho-Rb, total Rb, E2F1, and p21 levels were determined by immunoblotting. **D** E2F1 knockdown results in decreased DDB2 expression. E2F1 was silenced by lentiviral shRNA. The expression levels of E2F1 and DDB2 were measured by immunoblotting.

Our next objective was to investigate the mechanism through which pevonedistat downregulates DDB2. Quantitative RT-PCR (qRT-PCR) analysis revealed that pevonedistat decreases DDB2 expression at the transcriptional level in a dose-dependent manner (Fig. 5A). In order to determine the mechanism of DDB2 transcriptional downregulation, we transfected FaDu cells with a DDB2 promoter flanking a luciferase reporter. This assay demonstrated that treatment with pevonedistat significantly inhibited DDB2 promoter activity (Fig. 5B). The DDB2 promoter contains an E2F1 motif suggesting that E2F1 could be a key regulator of its expression [30]. In agreement with this hypothesis, it was previously reported that cells with elevated E2F1 levels or Rb deficiency both have higher basal levels of DDB2 and display a hyperactive NER pathway [31, 32]. p21 is among the most highly stabilized proteins following treatment with pevonedistat. p21 is responsible for the potent inhibition of CDK4/6 activity [33]. This inhibition of CDK4/6 results in the inhibition of Rb phosphorylation, which disallows the release of the transcription factor E2F1 [34]. To better define this mechanism in response to pevonedistat, we performed a series of immunoblotting experiments. We determined that Rb phosphorylation was decreased and p21 expression was increased in a dose-dependent manner following pevonedistat treatment, however, E2F1 protein expression was unaffected (Fig. 5C). To further evaluate whether E2F1 directly regulates DDB2 expression, we silenced E2F1 expression and showed that its knockdown significantly decreases DDB2 levels (Fig. 5D). Taken together, these findings demonstrate that pevonedistat downregulated DDB2 via inhibition of E2F1 activity.

### The pevonedistat and cisplatin combination yields long-term survival in a HNSCC xenograft model

**Fig. 6:**
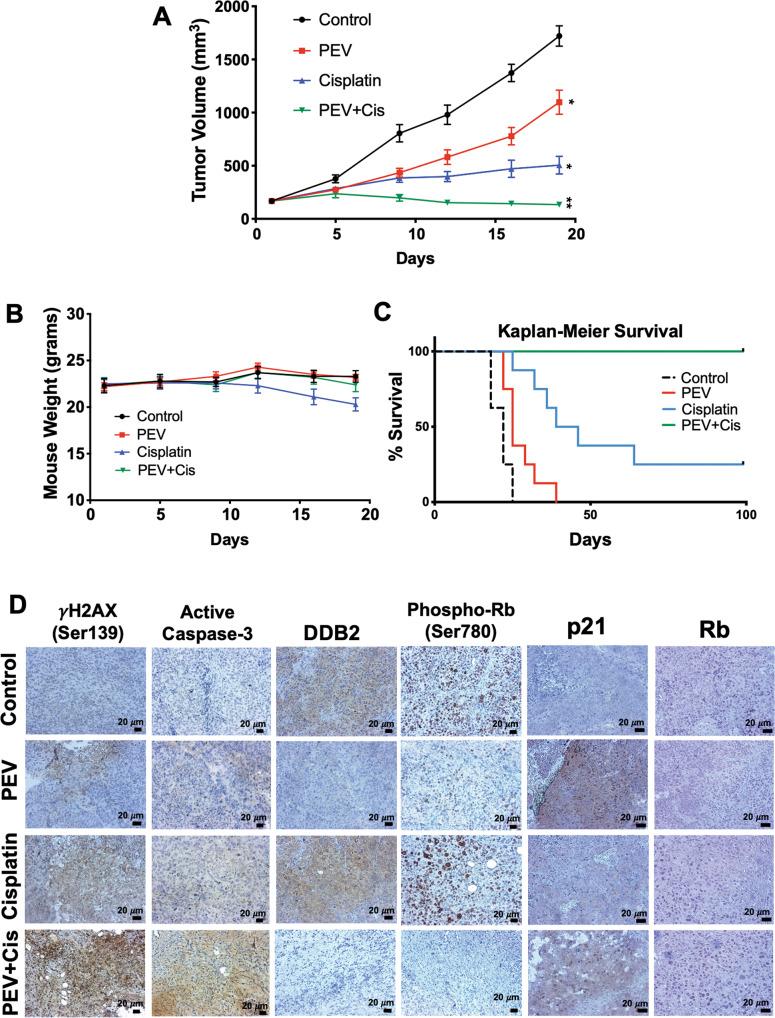
Pevonedistat cooperates with cisplatin to yield long-term survival. **A** The pevonedistat and cisplatin combination promotes tumor regression. FaDu tumors were established in mice. When tumors reach ~150 mm^3^ in size, mice were treated with vehicle control, 60 mg/kg pevonedistat SC QDx5, 3 mg/kg cisplatin IP twice a week, or the combination for approximately 3 weeks. Tumors were measured twice weekly during treatment. Mean ± SEM, *n* = 10 per group. **B** The combination of pevonedistat and cisplatin is well-tolerated in mice. Weight measurements were taken twice a week. Mean ± SEM, *n* = 10 per group. **C** The pevonedistat and cisplatin combination promotes long-term survival in tumor-bearing mice. FaDu tumors were established in mice and treated as described in A. Mice were monitored until Day 100 of the experiment. No signs of toxicity or disease were observed. Overall survival was determined by Kaplan–Meier survival analysis, *n* = 8 per group. **D** Pevonedistat decreases DDB2 expression in vivo and enhances cisplatin-induced γH2AX and cleaved caspase-3 levels in tumor specimens. γH2AX, cleaved caspase-3, DDB2, p21, phospho-Rb, and Rb expression was determined by immunohistochemistry.

We next investigated the therapeutic potential of combining pevonedistat and cisplatin in vivo using the FaDu HNSCC xenograft mouse model. Mice were treated with pevonedistat (60 mg/kg SC, 5 times per week), cisplatin (3 mg/kg IP, twice a week), or the combination for 3 weeks. While both monotherapies significantly decreased tumor burden, the combination resulted in tumor regression (Fig. 6A and Supplementary Table S3). Importantly, pevonedistat monotherapy and the combination were very well tolerated with no significant weight loss or observable signs of toxicity (Fig. 6B). Treatment was stopped after three weeks (21 days) and mice were continuously monitored for 100 days to document animal survival (Fig. 6C). Remarkably, all mice receiving the pevonedistat and cisplatin combination survived out to day 100 with no evidence of tumor recurrence, reenforcing our finding that tumor regression was significant and sustained. The combination regimen was significantly superior with respect to prolonging survival compared to standard cisplatin treatment (Supplementary Table S4). Immunohistochemistry (IHC) PD analyses were performed on paraffin-embedded tumor samples excised from mice in each group (Fig. 6D and Supplementary Fig. S8). Both γH2AX and active caspase-3 were highly elevated in tumors subjected to combination therapy. Similar to our in vitro results, DDB2 and phospho-RB levels were significantly lower in the pevonedistat single agent and combination groups. Furthermore, p21 was shown to be stabilized in tumor samples subjected to both pevonedistat monotherapy and combination treatment. Collectively, our results demonstrate that systemic administration of pevonedistat significantly decreases DDB2 expression, which promotes enhanced sensitivity to cisplatin resulting in long-term survival of HNSCC tumor-bearing animals.

## Discussion

While it remains a mainstay in the clinical management of HNSCC, cisplatin chemotherapy provides less than satisfactory outcomes. Unfortunately, more than half of patients with HPV-negative HNSCC who receive a cisplatin-based regimen will relapse within two years. At recurrence, median overall survival is ~1 year even with salvage treatment with modern targeted and immune therapies [35]. Despite advances in surgery, radiation therapy, and systemic therapies, the survival of HPV-negative HNSCC remain virtually unchanged in the past few decades [36]. Very few compounds targeting key oncogenic pathways in HNSCC have shown efficacy when introduced into the clinical setting [37]. Therefore, there is a critical and urgent unmet medical need to investigate therapeutics that target novel pathways to achieve better outcomes for patients.

Here we report that NEDD8 expression is significantly higher in HNSCC compared to adjacent normal tissue suggesting that targeting NEDDylation may be a novel approach to treat this tumor type. Consistent with high NEDD8 levels, we demonstrated that HPV-negative HNSCC cell lines display heightened sensitivity to pevonedistat. Previous studies have determined that inhibition of NEDDylation exhibits promising efficacy against HNSCC in preclinical studies. These investigations report that pevonedistat suppresses tumor cell growth and induce apoptosis through several mechanisms including activation of both the intrinsic and extrinsic apoptotic cascades [21, 22, 38]. Specifically, pevonedistat stimulated apoptosis via induction of the c-MYC-NOXA and ATF4-CHOP-DR5 pathways [23, 38]. In agreement with these studies, we further show that pevonedistat demonstrates significant activity as a monotherapy in HNSCC models.

Despite the significant anticancer activity of pevonedistat, its optimal application is likely to be as a component of rational combination regimens. We determined that pevonedistat synergistically enhanced DNA damage-mediated apoptosis induced by cisplatin treatment, the mainstay of curative-intent multimodality management of HNSCC. While the benefit of pevonedistat in combination with various DNA damage-inducing agents has been reported in other models, the mechanism of action of this therapeutic approach remains to be fully elucidated [24, 25, 27, 39–41]. We discovered that targeted inhibition of CUL4A drives the synergy of pevonedistat with cisplatin by suppressing DNA damage repair. Conversely, overexpression of CUL4A reduced cisplatin efficacy in HNSCC models. Since CUL4A has been previously reported to be overexpressed in nasopharyngeal and esophageal squamous cell carcinoma, this provides further rationale for the investigation of pevonedistat for HNSCC therapy [42, 43]. Surprisingly, the augmentation of cisplatin-induced DNA damage and apoptosis by pevonedistat or CUL4A knockdown was shown to be mediated through the downregulation of DDB2, which is required for NER. As NER is the primary DNA repair pathway induced by cisplatin-triggered DNA damage, it is a major mechanism that limits cisplatin efficacy and promotes drug resistance [44]. Our data demonstrates that targeting CUL4A genetically or with pevonedistat effectively antagonizes NER by decreasing DDB2 expression. This indicates that pevonedistat treatment may represent a novel precision strategy to enhance cisplatin efficacy against HNSCC cells that exhibit hyperactive NER.

DDB2 has been characterized as a tumor suppressor gene as its mutation results in NER defects and consequential genetic instability. Consistent with this role, DDB2-deficient mice spontaneously develop tumors [45]. However, evidence suggests that DDB2 may function as a double-edged sword with differing functions in pre-malignant vs. malignant cells. Indeed, DDB2-mediated DNA repair has been implicated in resistance to DNA damage-inducing chemotherapy. Considering this, inhibition of DDB2 may be a promising strategy to augment the efficacy of multiple classes of anticancer DNA damaging agents [46]. In our study, we discovered that pevonedistat decreases DDB2 expression at the transcriptional level. While p53 is known to be a significant inducer of DDB2 expression, *TP53* is mutated in most HPV-negative HNSCC, indicating that other regulators of DDB2 are likely more important in this scenario. We and others have demonstrated that pevonedistat dramatically increases the expression of p21 [25, 47, 48]. Overexpression of p21 prevents the phosphorylation of Rb and the subsequent activation of E2F1 [33, 34]. Our study demonstrates that pevonedistat inhibits E2F1-mediated transcription of DDB2. As DDB2 is a key regulator of cisplatin sensitivity, our collective results show that pevonedistat-mediated downregulation of DDB2 is a novel mechanism promoting the synergistic activity of the pevonedistat and cisplatin combination.

To further evaluate the pevonedistat and cisplatin combination for HNSCC therapy, we investigated its efficacy using HNSCC xenograft models. Consistent with our in vitro studies, we detected a significant decrease in DDB2 and upregulation of p21 in tumors treated with pevonedistat. Enhanced levels of apoptosis were also observed in pevonedistat and cisplatin-treated tumors compared to either monotherapy. Importantly, we found that combination therapy resulted in tumor regression in all mice resulting in complete animal survival at Day 100. These results suggest that further evaluation of the pevonedistat and cisplatin combination is warranted in patients with HPV-negative HNSCC.

Pevonedistat is currently demonstrating promising clinical efficacy in the treatment of patients with acute myeloid leukemia (AML). Similar to our findings in HNSCC, preclinical studies reported potent activity of pevonedistat in AML xenograft models [49]. Consistent with its ability to augment DNA damage inducing therapy reported in our study, pevonedistat demonstrated significant ability to enhance the anticancer activity of cytarabine and azacitidine [27, 50]. While our study specifically addressed the relationship between cisplatin and pevonedistat, these results describe a mechanism which may have wide-ranging implications. Many other anticancer agents, such as radiation therapy and 5-fluorouracil, also rely on DNA damage to kill malignant cells. A therapeutic combination including pevonedistat may prove to be an effective approach to inhibit the DNA repair pathways that often culminate in resistance to these treatments. Indeed, our results may explain, in part, why pevonedistat augments the efficacy of multiple cytotoxic anticancer agents. Further studies, including clinical trials, are warranted to fully evaluate the clinical potential of pevonedistat as therapeutic partner with cisplatin in the treatment of HPV-negative HNSCC.

## Supplementary information

Supplementary Figures and Tables

Supplementary Figure S1

Supplementary Figure S2

Supplementary Figure S3

Supplementary Figure S4

Supplementary Figure S5

Supplementary Figure S6

Supplementary Figure S7

Supplementary Figure S8

Supplementary Table S1

Supplementary Table S2

Supplementary Table S3

Supplementary Table S4

Original Data File

Checklist

## Data Availability

All data needed to evaluate the conclusions of this study are present in the manuscript. Requests for additional information related to this paper should be directed to the corresponding author.
