# Supplementary material for: Targeted CUL4A inhibition synergizes with cisplatin to yield long-term survival in models of head and neck squamous cell carcinoma through a DDB2-mediated mechanism
Source: Cell Death Dis. 2022 Apr 15;13(4):350. doi: 10.1038/s41419-022-04798-6 (PMC9012827; doi:10.1038/s41419-022-04798-6)
Supplement: Supplementary file 1 — Supplementary Figures and Tables [file 41419_2022_4798_MOESM1_ESM.pdf]

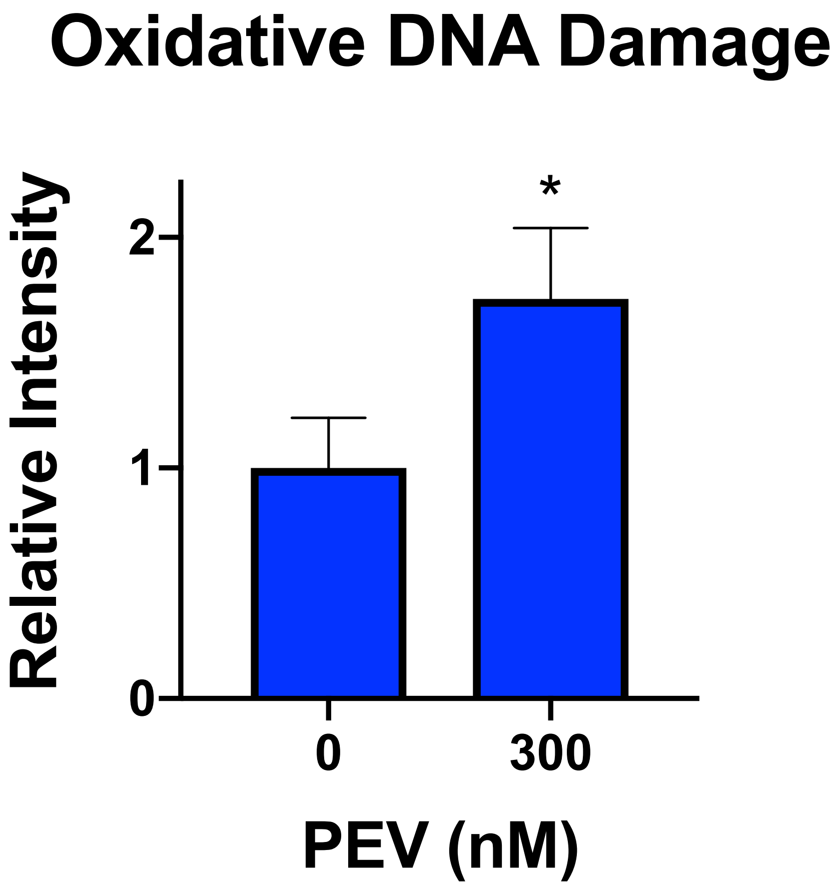

Supplementary Figure S1. PEV treatment induces oxidative DNA damage. FaDu cells were treated with PEV for 24 h. Oxidative DNA damage was measured by staining with a FITC-tagged oxidative DNA antibody and quantified by flow cytometry. Mean +/- SD, n = 3. * Indicates a significant difference from control, $p < 0.05$.

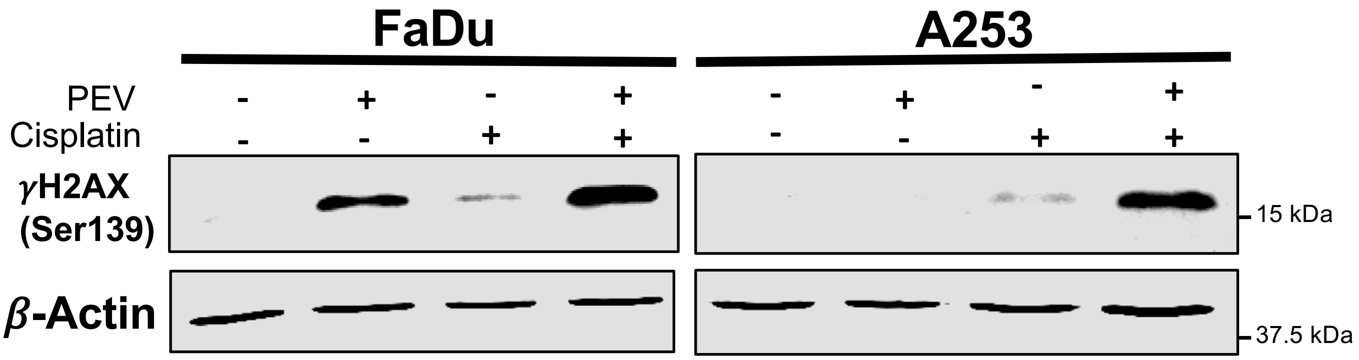

Supplementary Figure S2. The PEV and cisplatin combination significantly increase γH2AX expression. FaDu and A253 cells were treated with 600 nM PEV, 3 μM cisplatin, or the combination for 24 h. γH2AX levels were determined by immunoblotting.

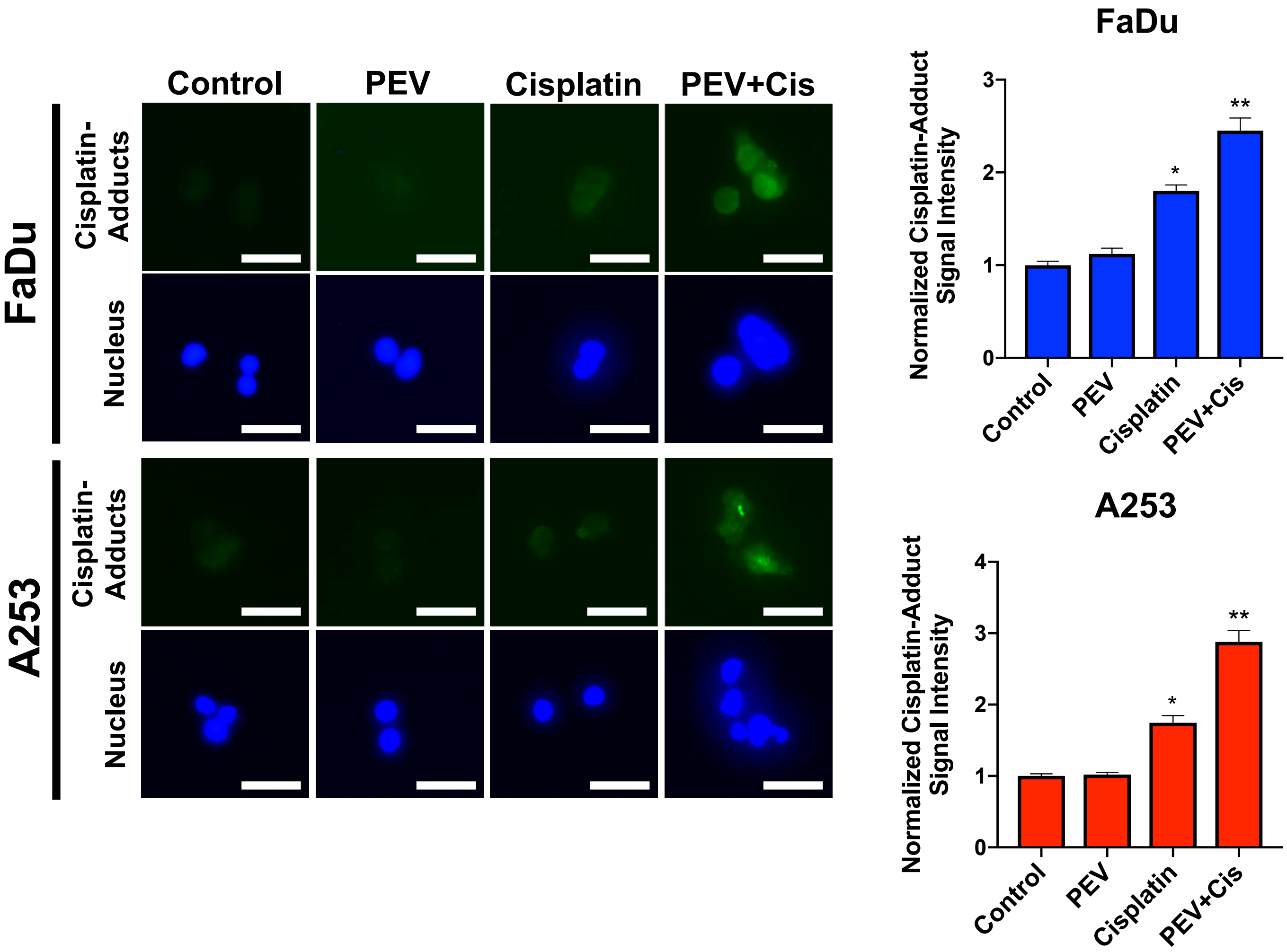

Supplementary Figure S3. The PEV and cisplatin combination significantly increase cisplatin-DNA adduct levels. FaDu and A253 cells were treated with 600 nM PEV, 3 μM cisplatin, or the combination for 18 h. Immunocytochemistry was used to stain for cisplatin adducts. Mean +/- SEM, n = 25. * Denotes a significant difference from control. ** Represents a significant difference from either monotherapy, $p < 0.05$.

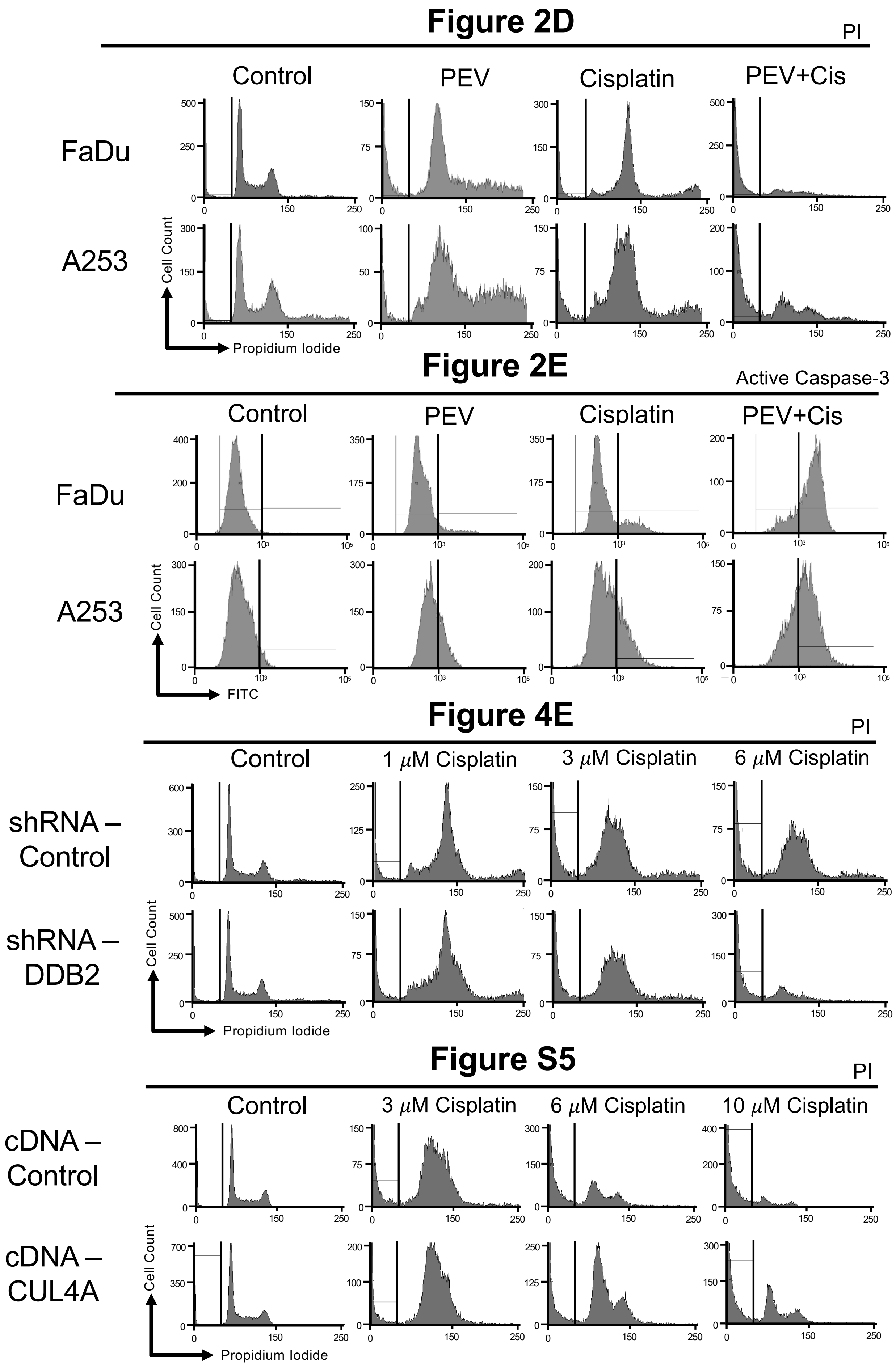

Supplementary Figure S4. Representative histograms of flow cytometry experiments.

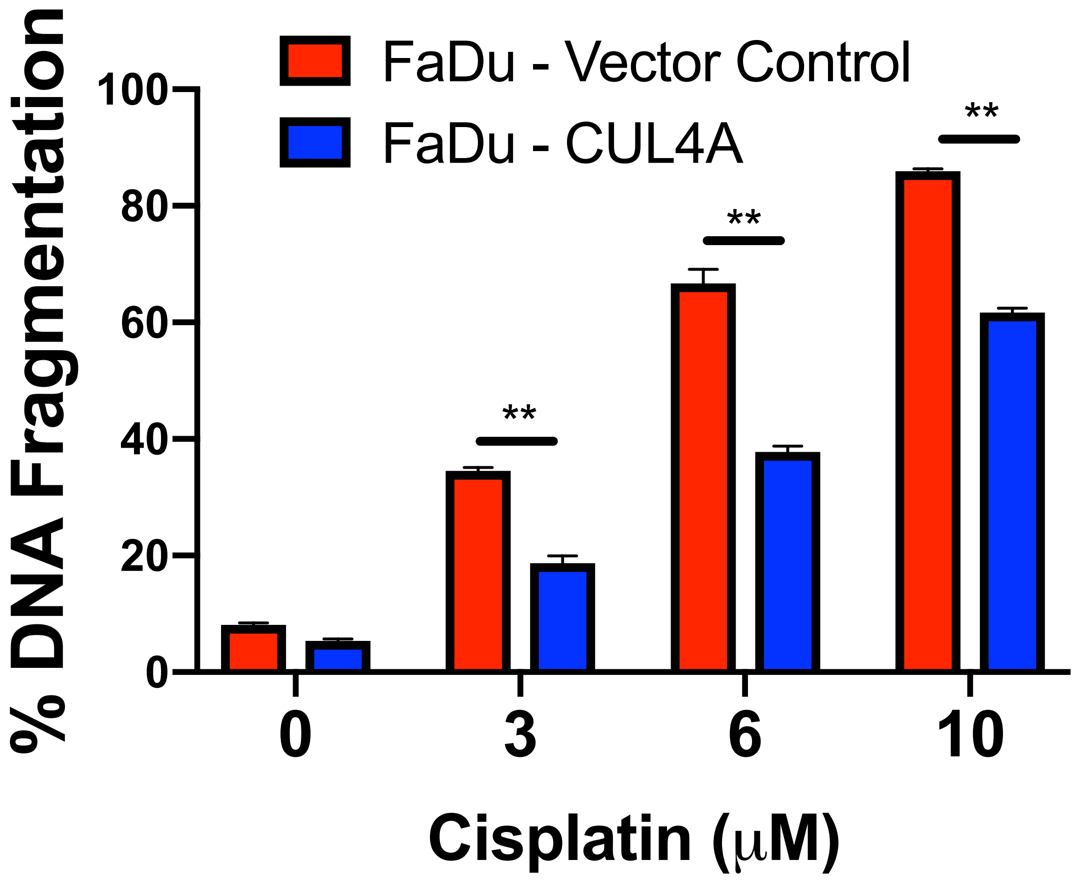

Supplementary Figure S5. Overexpression of CUL4A blunts cisplatin-mediated apoptosis. FaDu cells were transfected with Control or CUL4A overexpression plasmids and placed under puromycin selection. Cells were treated with the indicated concentrations of cisplatin for 24 h and apoptosis was measured by PI-FACS analysis. Mean +/- SD, n = 3. ** Denotes a significant difference between the indicated samples, $p < 0.01$.

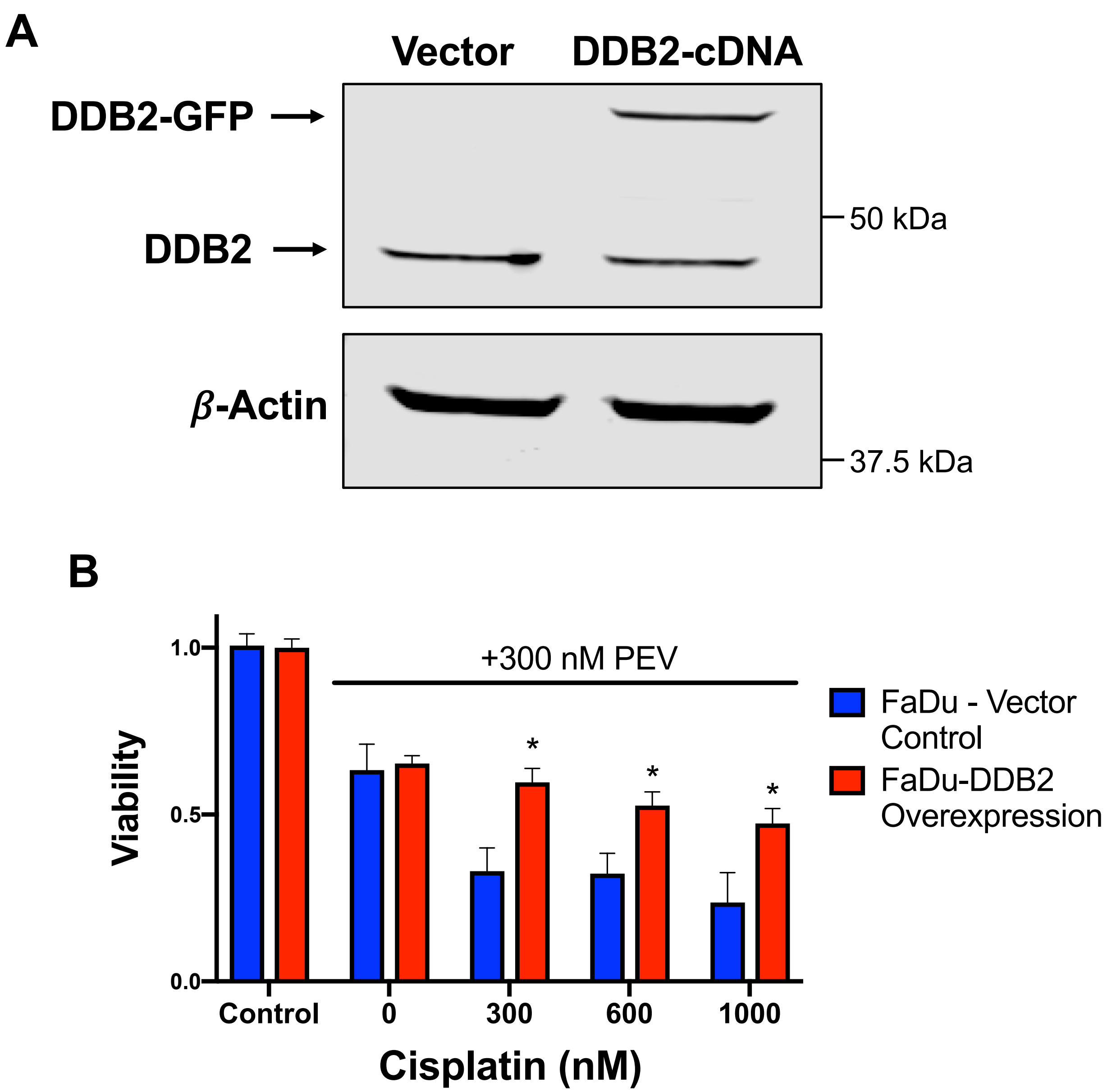

Supplementary Figure S6. Overexpression of DDB2 promotes resistance to the PEV and cisplatin combination. FaDu cells were transfected with scramble control or DDB2-GFP overexpression vectors and placed under puromycin selection. **A**, Protein expression of DDB2 was measured by immunoblotting. **B**, Vector control and DDB2-overexpression (OE) cell lines were treated with 300 nM PEV with or without increasing concentrations of cisplatin for 72 h. Cell viability was assessed by MTT assay. Mean +/- SD, n = 3. * Denotes significance between control and DDB2-OE samples, $p < 0.05$.

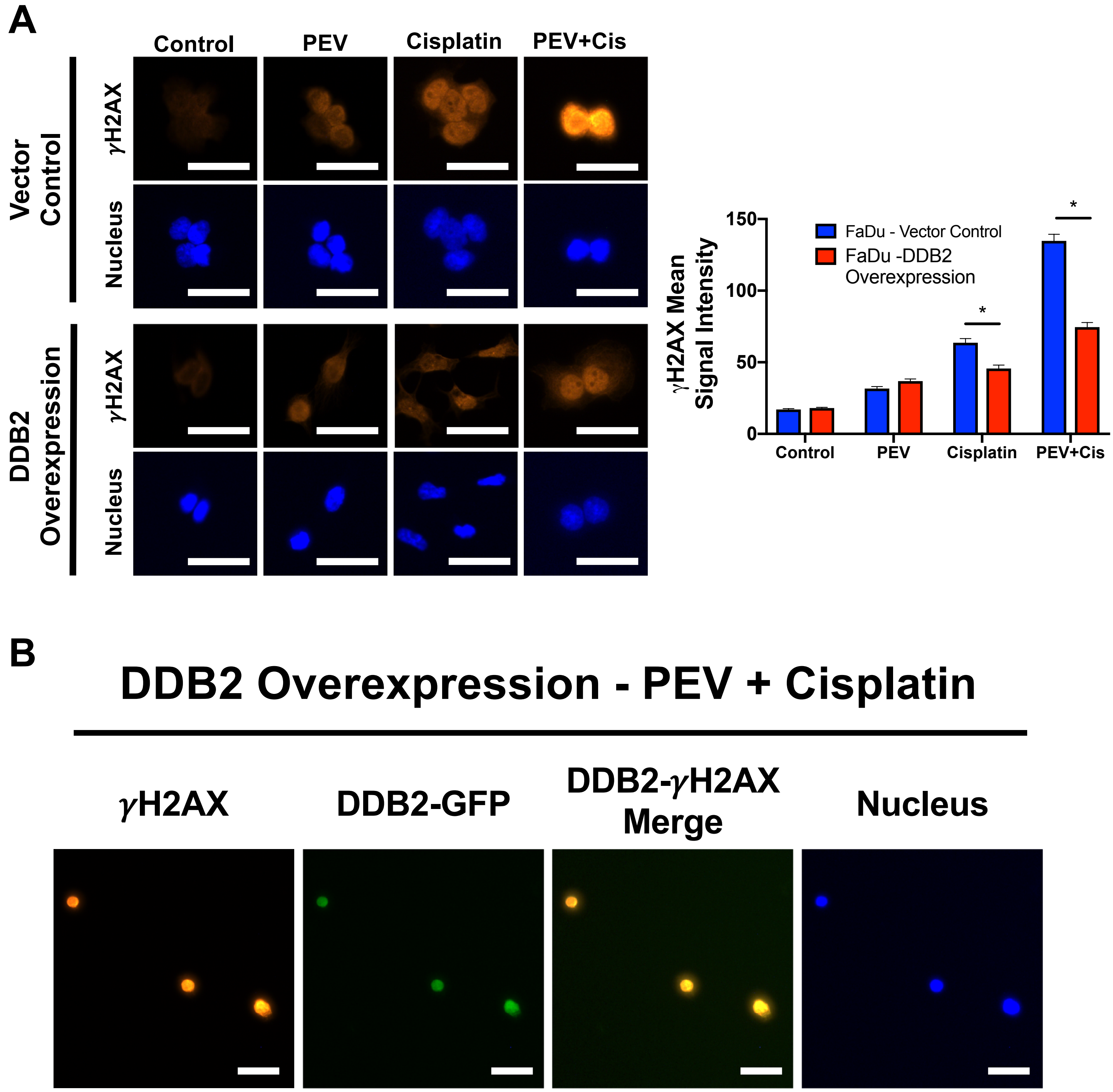

Supplementary Figure S7. DDB2 overexpression yields lower levels of γH2AX in response to cisplatin and PEV treatment. **A**, Cells transfected with control and DDB2 overexpression vectors were treated with 600 nM PEV, 5 μM cisplatin, or the combination for 24 h. Immunocytochemistry was used to quantify γH2AX expression. Mean +/- SEM, n = 25, *p < 0.05. **B**, Representative images of the localization of GFP-tagged DDB2 in treated cells.

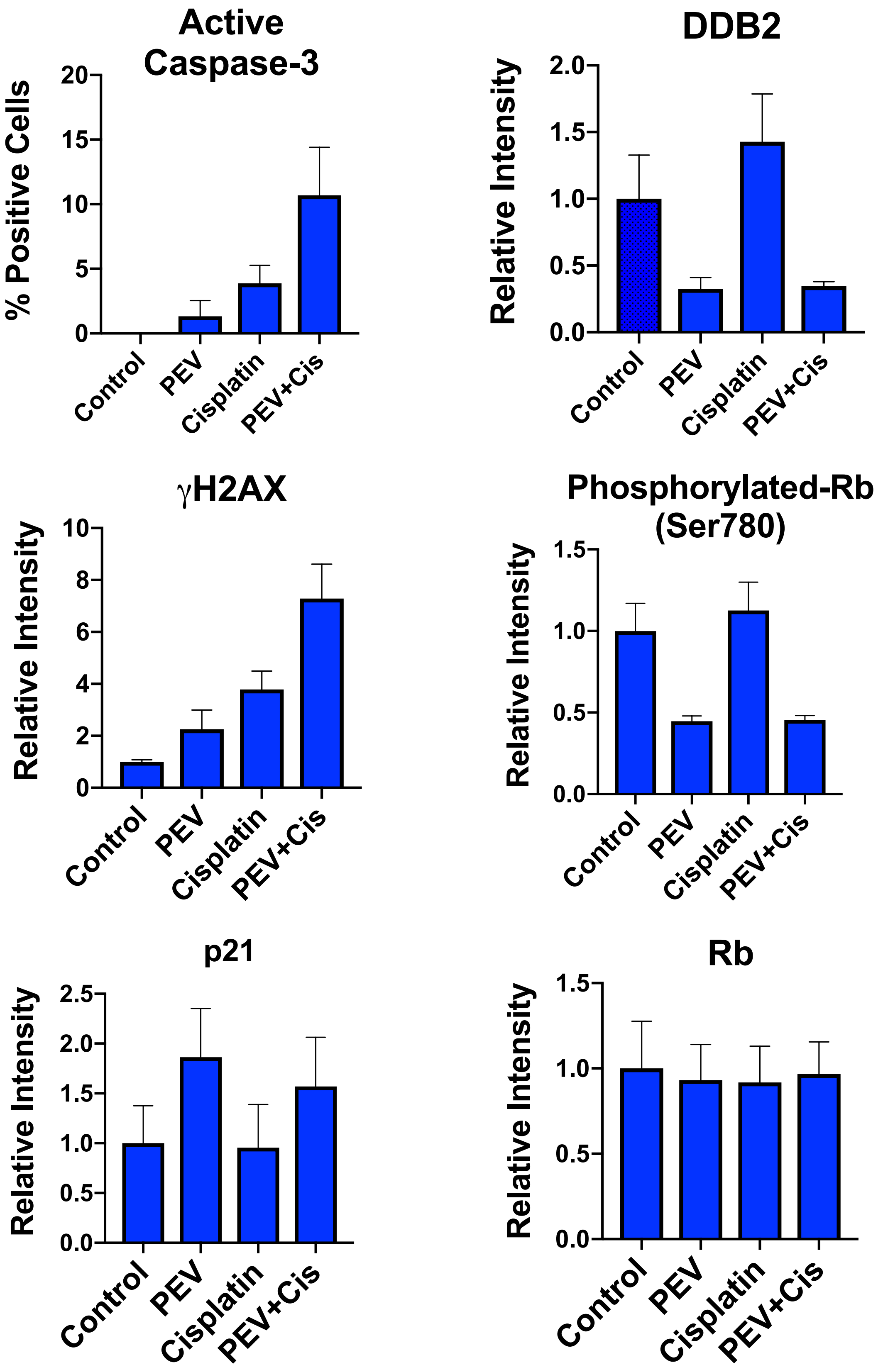

Supplementary Figure S8. Quantification of IHC Images. IHC images were quantified using color deconvolution analysis in ImageJ software. Five representative images of each tumor sample were used for quantification.

Supplementary Table S1. Percentage of cells analyzed in Supplementary Figure S3 that were positive for cisplatin-DNA adducts.

	Control	PEV	Cisplatin	PEV+Cis
FaDu	0.0 %	0.0 %	53.3 %	93.9 %
A253	0.0 %	0.0 %	72.0 %	100.0 %

Supplementary Table S2. Combination Index (CI) tables for FaDu and A253 cells treated with PEV and cisplatin.

FaDu

PEV (nM)	Cisplatin (nM)	CI
300	10	0.15590
300	30	0.14946
300	100	0.13767
300	300	0.15166
300	1000	0.19638
300	3000	0.24200

PEV (nM)	Cisplatin (nM)	CI
10.0	3000.0	0.25013
30.0	3000.0	0.26287
100.0	3000.0	0.16951
300.0	3000.0	0.13477
1000.0	3000.0	0.15375
3000.0	3000.0	0.25573

A253

PEV (nM)	Cisplatin (nM)	CI
300	10	0.35162
300	30	0.34036
300	100	0.30885
300	300	0.26866
300	1000	0.20299
300	3000	0.13352

PEV (nM)	Cisplatin (nM)	CI
10.0	3000.0	0.09384
30.0	3000.0	0.09657
100.0	3000.0	0.10615
300.0	3000.0	0.12569
1000.0	3000.0	0.32199
3000.0	3000.0	0.47235

Supplementary Table S3. Mean xenograft tumor sizes. Mean +/- SEM, n = 10.

Day	Control	PEV	Cisplatin	PEV+Cis
1	167.9 ± 7.6	168.5 ± 5.74	168.6 ± 5.05	168.2 ± 7.28
5	376.8 ± 37.1	274.9 ± 23.07	285.2 ± 22.8	236 ± 38.06
9	805.7 ± 82.1	434.5 ± 41.1	385.3 ± 41.21	197.7 ± 30.90
12	979.7 ± 91.6	581.6 ± 68.94	397.7 ± 47.54	152.4 ± 20.07
16	1373 ± 81.1	778.4 ± 81.17	471.1 ± 80.5	143 ± 11.53
19	1721.2 ± 96	1097.6 ± 113.03	505.9 ± 82.42	134.5 ± 7.48

Supplementary Table S4. The combination of PEV and cisplatin confers long-term survival benefit in FaDu tumor-bearing mice.

Treatment Group	Median Survival
Control	22 Days
PEV	25 Days
Cisplatin	42.5 Days
PEV+Cis	>100 Days
