# Supplementary figures and images for: Targeted CUL4A inhibition synergizes with cisplatin to yield long-term survival in models of head and neck squamous cell carcinoma through a DDB2-mediated mechanism
Source: Cell Death Dis. 2022 Apr 15;13(4):350. doi: 10.1038/s41419-022-04798-6 (PMC9012827; doi:10.1038/s41419-022-04798-6)

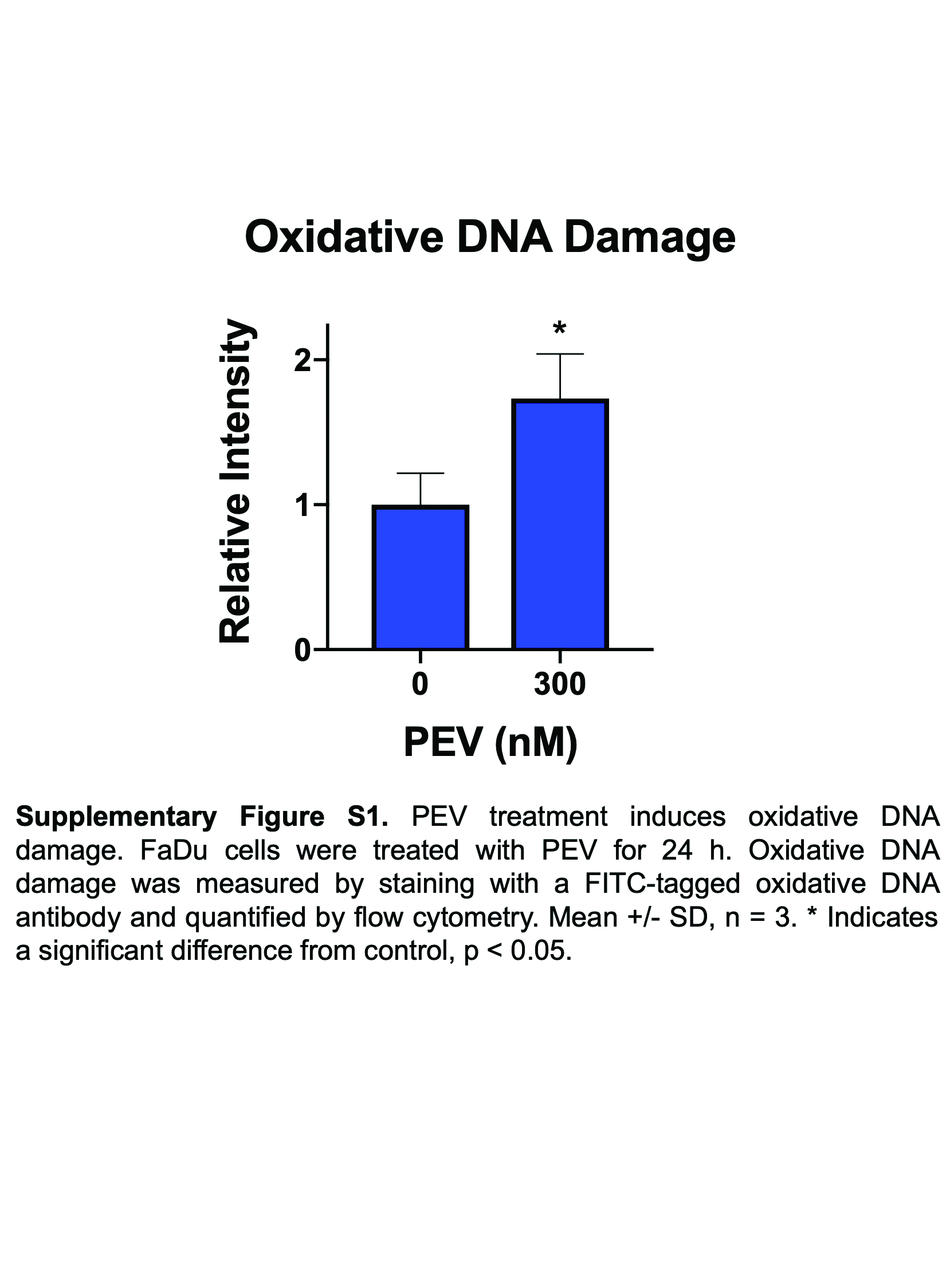

Supplement: Supplementary file 2 — Supplementary Figure S1 [file 41419_2022_4798_MOESM2_ESM.tif]

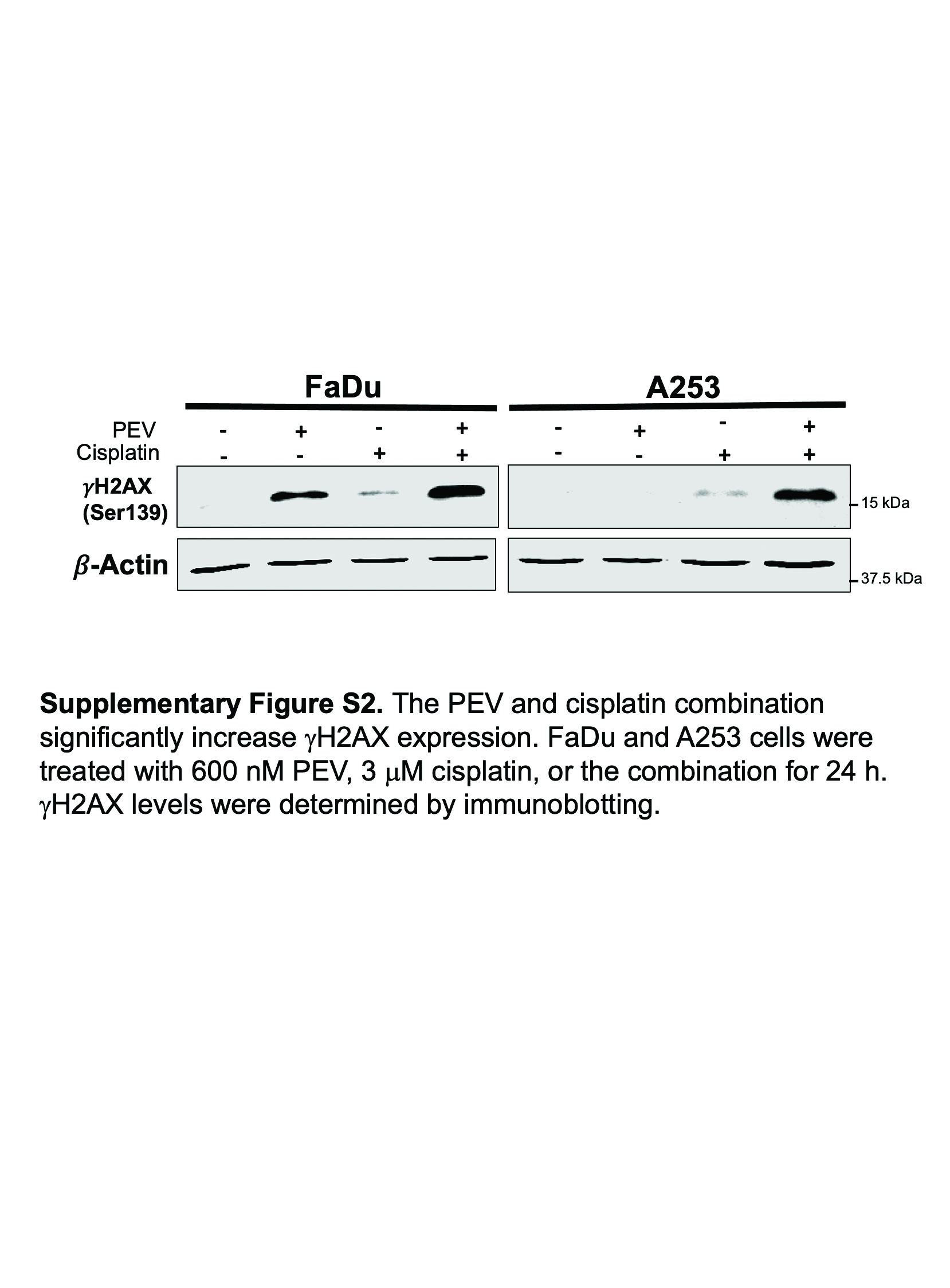

Supplement: Supplementary file 3 — Supplementary Figure S2 [file 41419_2022_4798_MOESM3_ESM.tif]

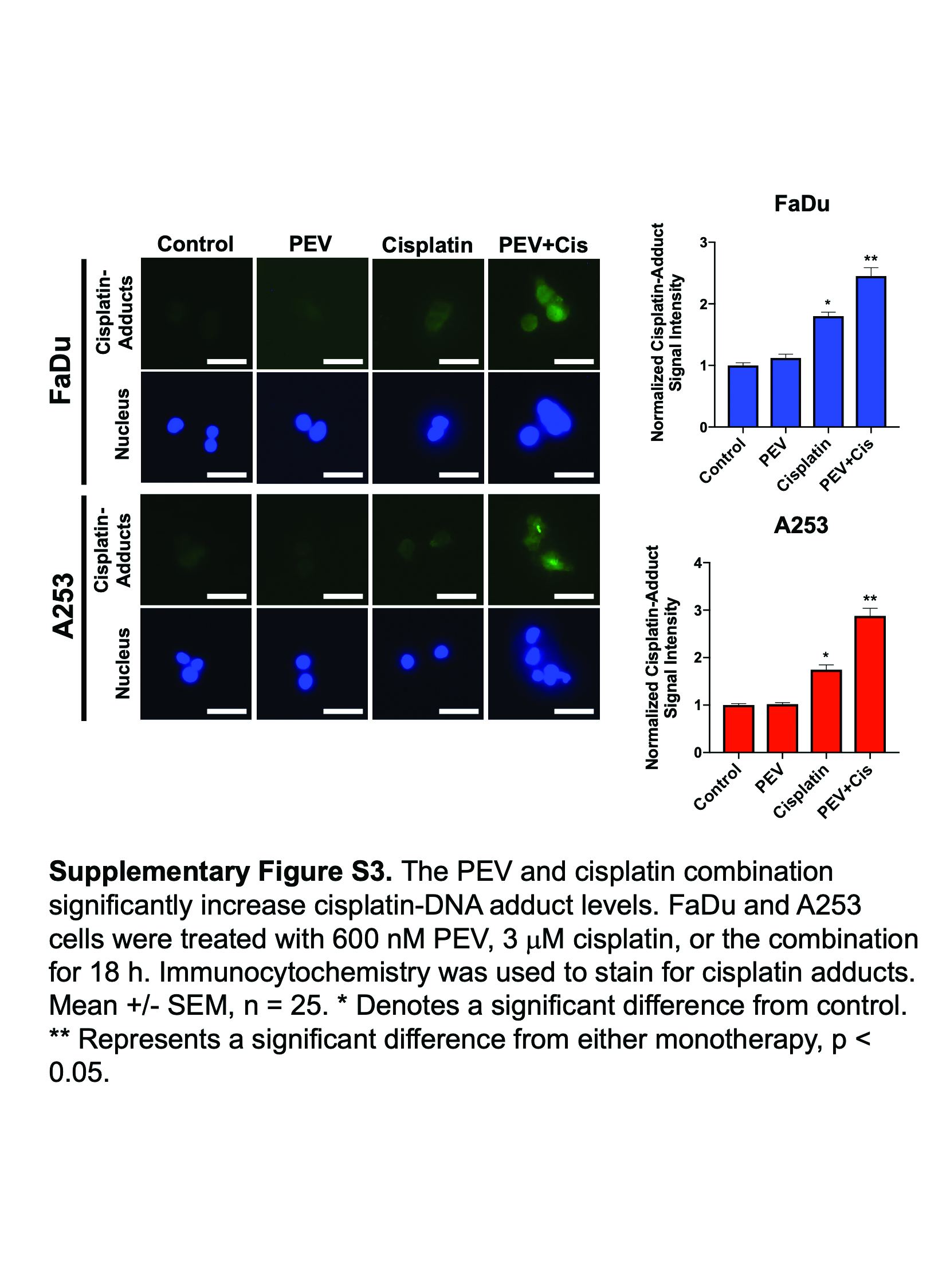

Supplement: Supplementary file 4 — Supplementary Figure S3 [file 41419_2022_4798_MOESM4_ESM.tif]

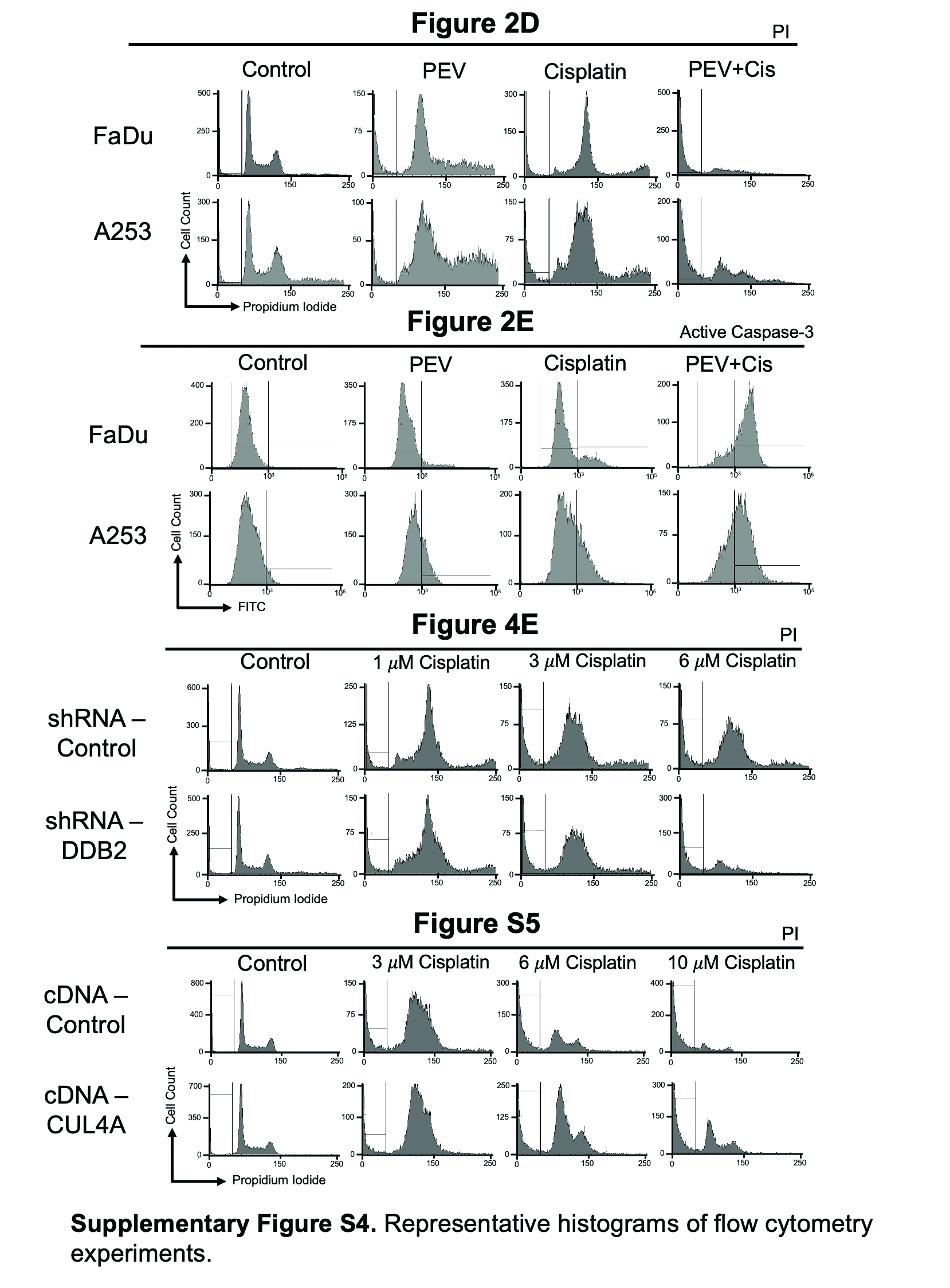

Supplement: Supplementary file 5 — Supplementary Figure S4 [file 41419_2022_4798_MOESM5_ESM.tif]

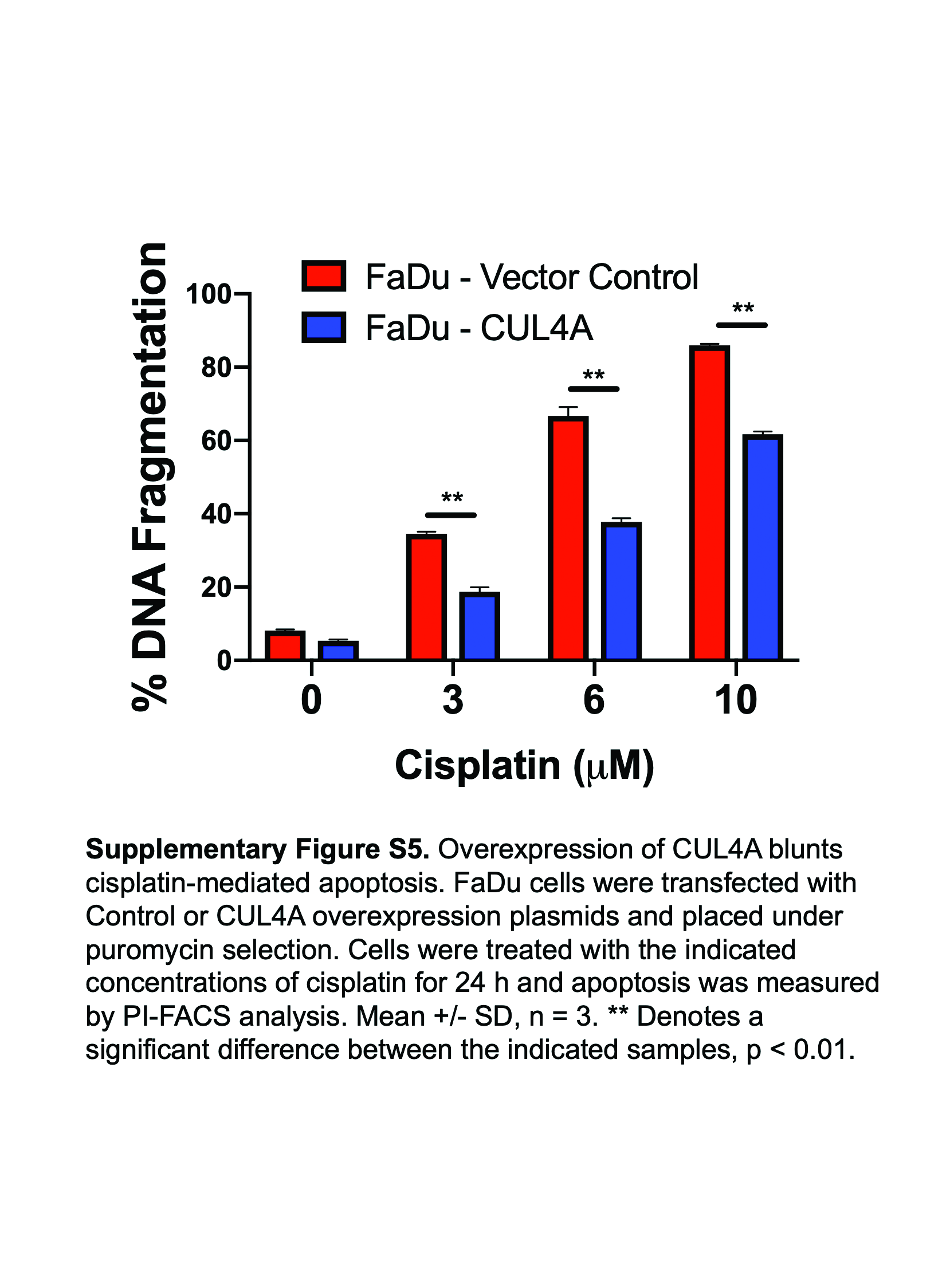

Supplement: Supplementary file 6 — Supplementary Figure S5 [file 41419_2022_4798_MOESM6_ESM.tif]

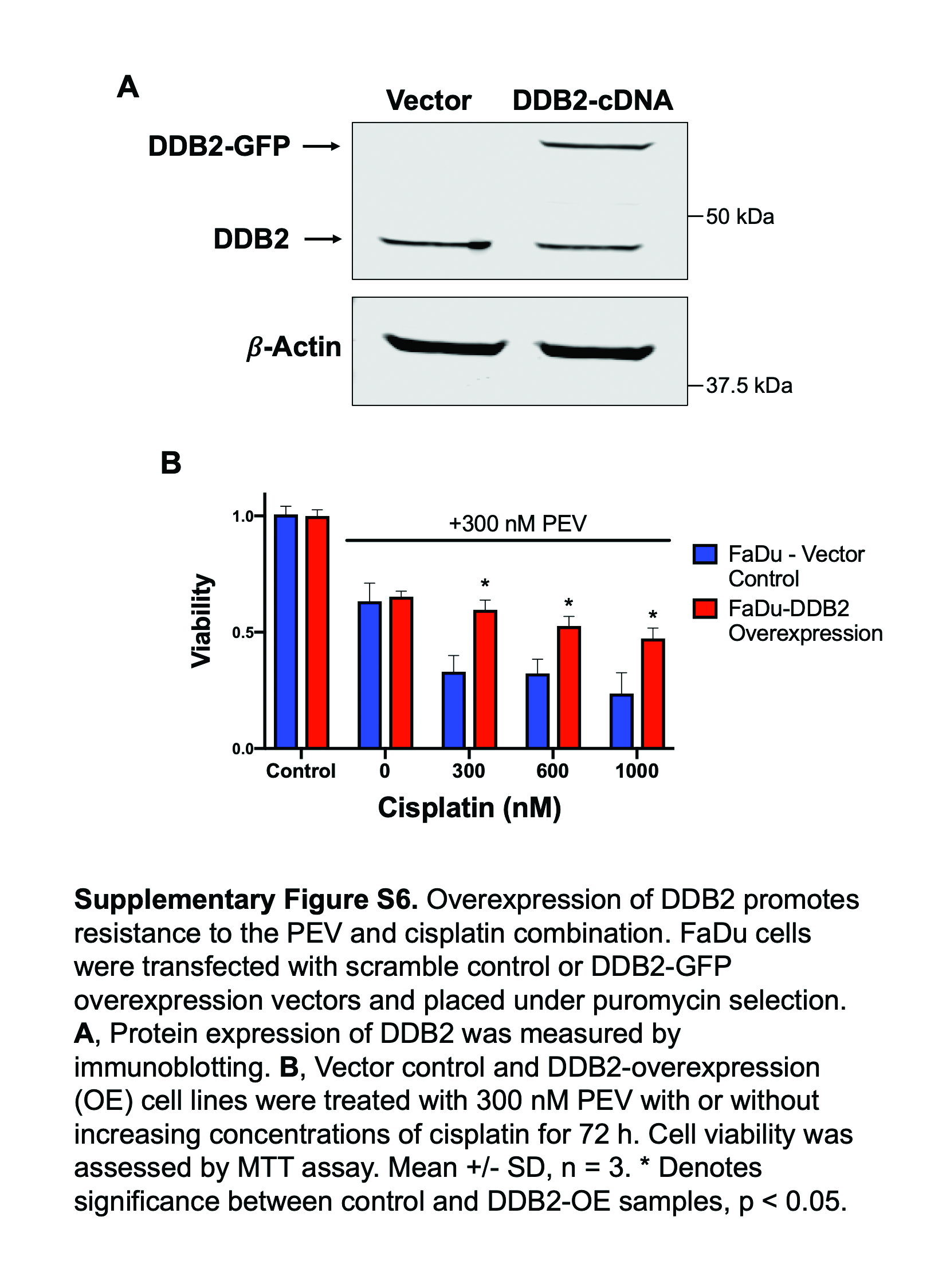

Supplement: Supplementary file 7 — Supplementary Figure S6 [file 41419_2022_4798_MOESM7_ESM.tif]

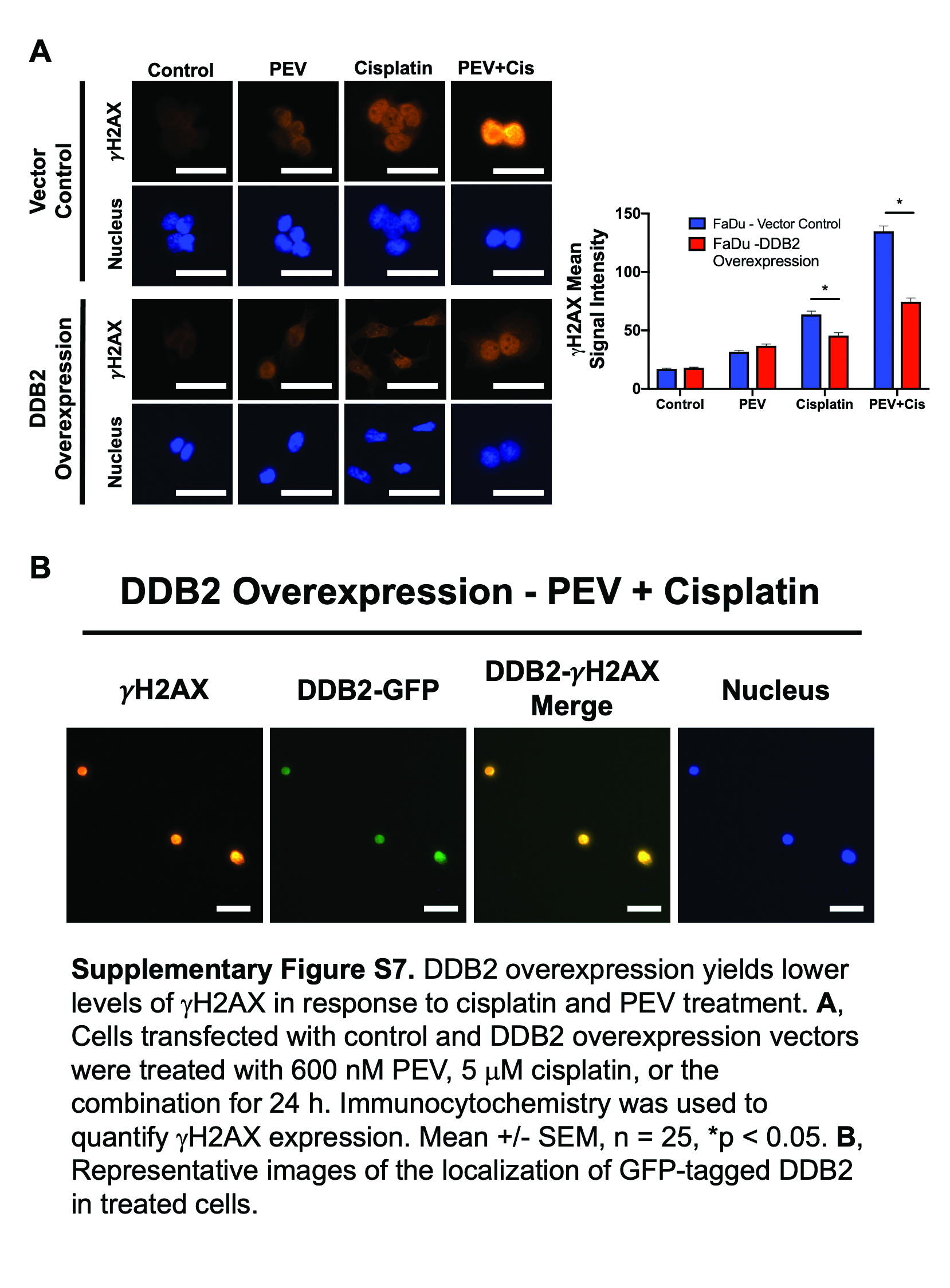

Supplement: Supplementary file 8 — Supplementary Figure S7 [file 41419_2022_4798_MOESM8_ESM.tif]

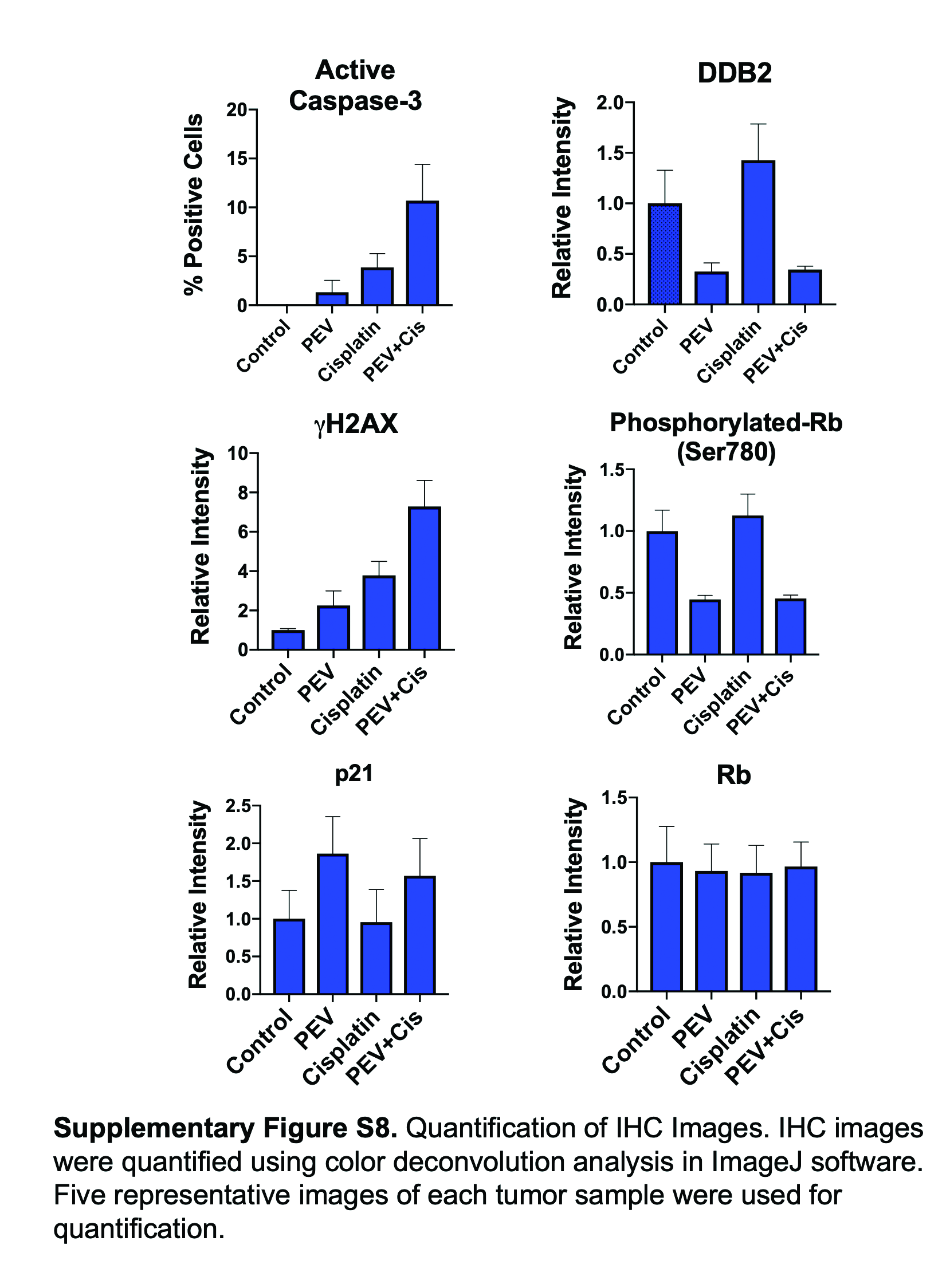

Supplement: Supplementary file 9 — Supplementary Figure S8 [file 41419_2022_4798_MOESM9_ESM.tif]
