# Supplementary material for: Targeted CUL4A inhibition synergizes with cisplatin to yield long-term survival in models of head and neck squamous cell carcinoma through a DDB2-mediated mechanism
Source: Cell Death Dis. 2022 Apr 15;13(4):350. doi: 10.1038/s41419-022-04798-6 (PMC9012827; doi:10.1038/s41419-022-04798-6)
Supplement: Supplementary file 14 — Original Data File [file 41419_2022_4798_MOESM14_ESM.pdf]

Figure 1C

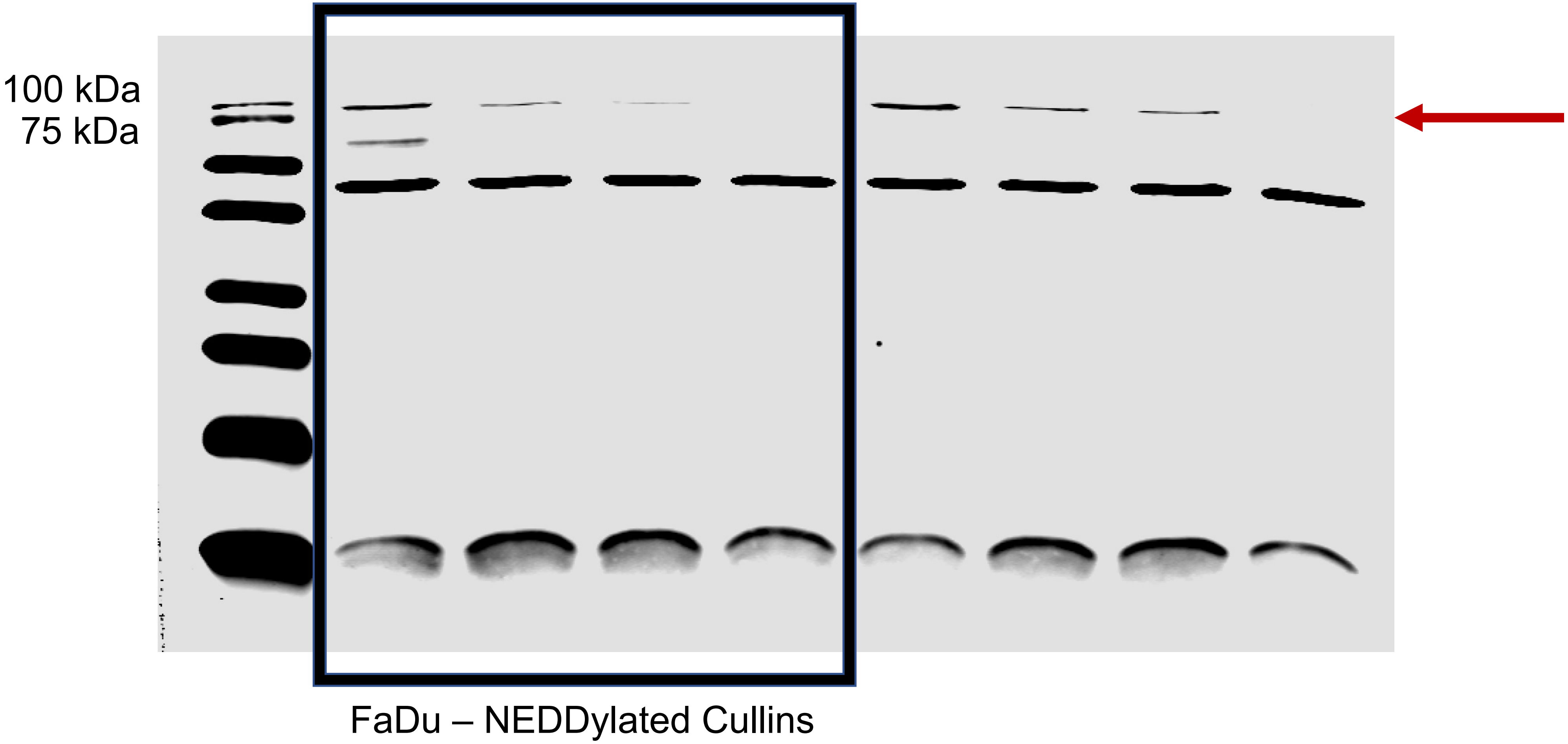

Figure 1C

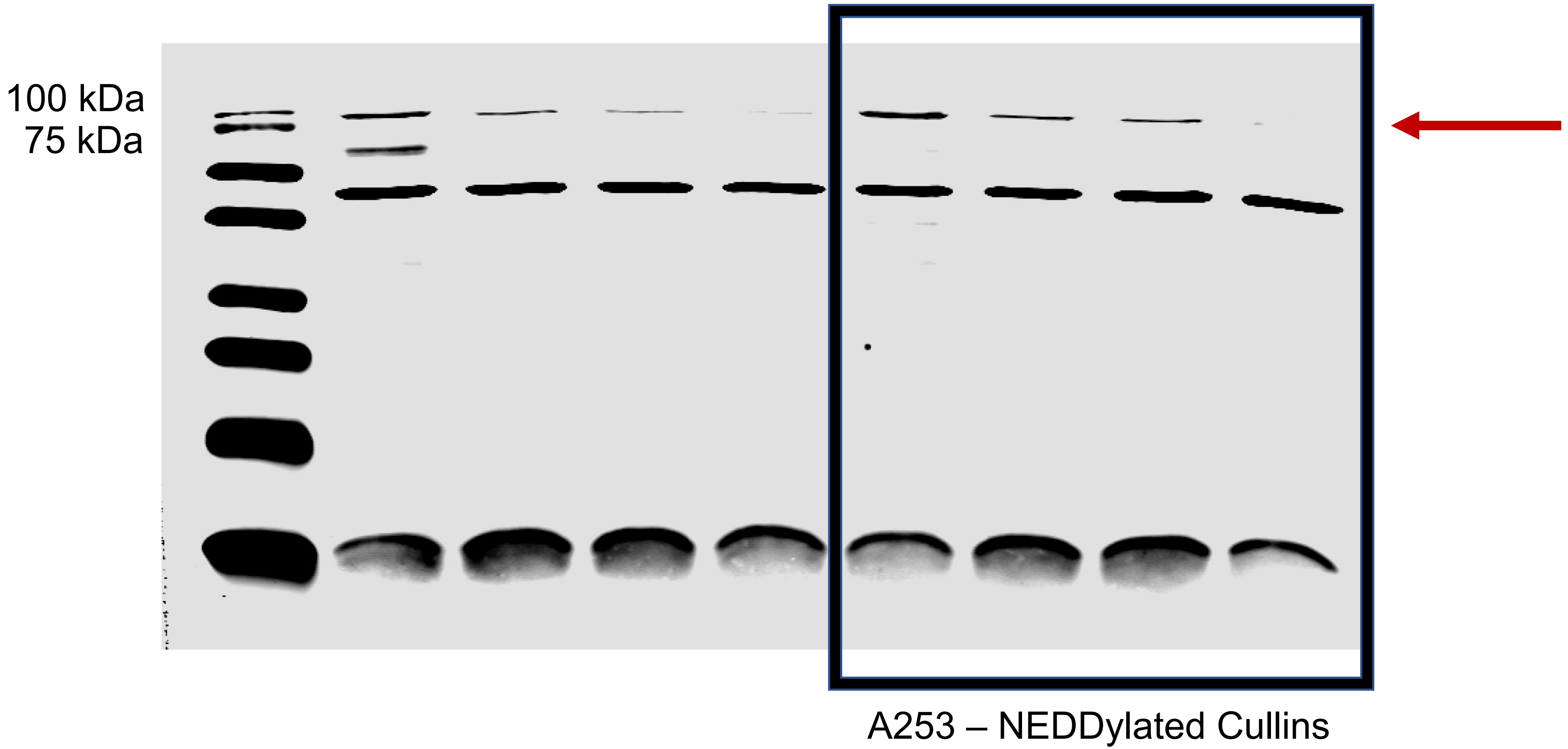

Figure 1C

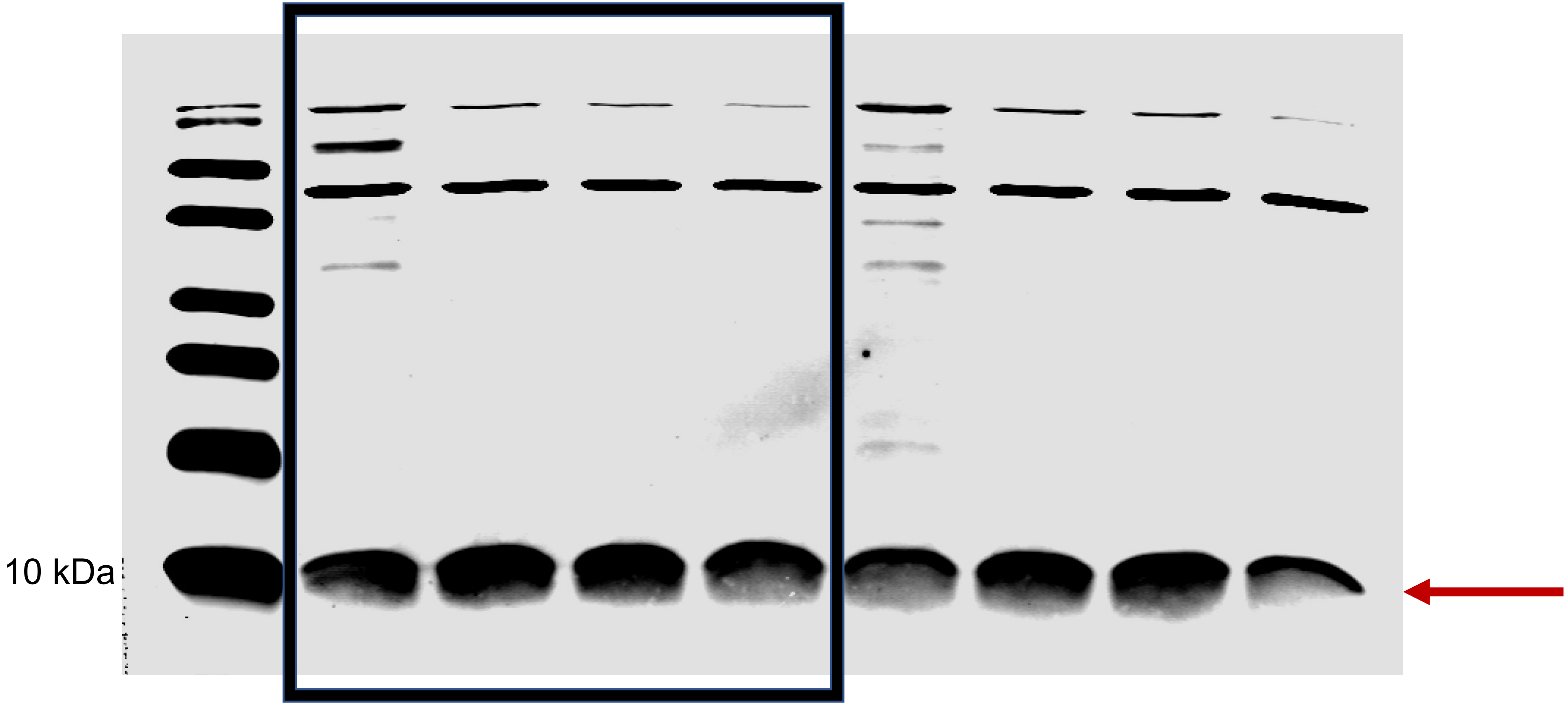

FaDu – Free NEDD8

Figure 1C

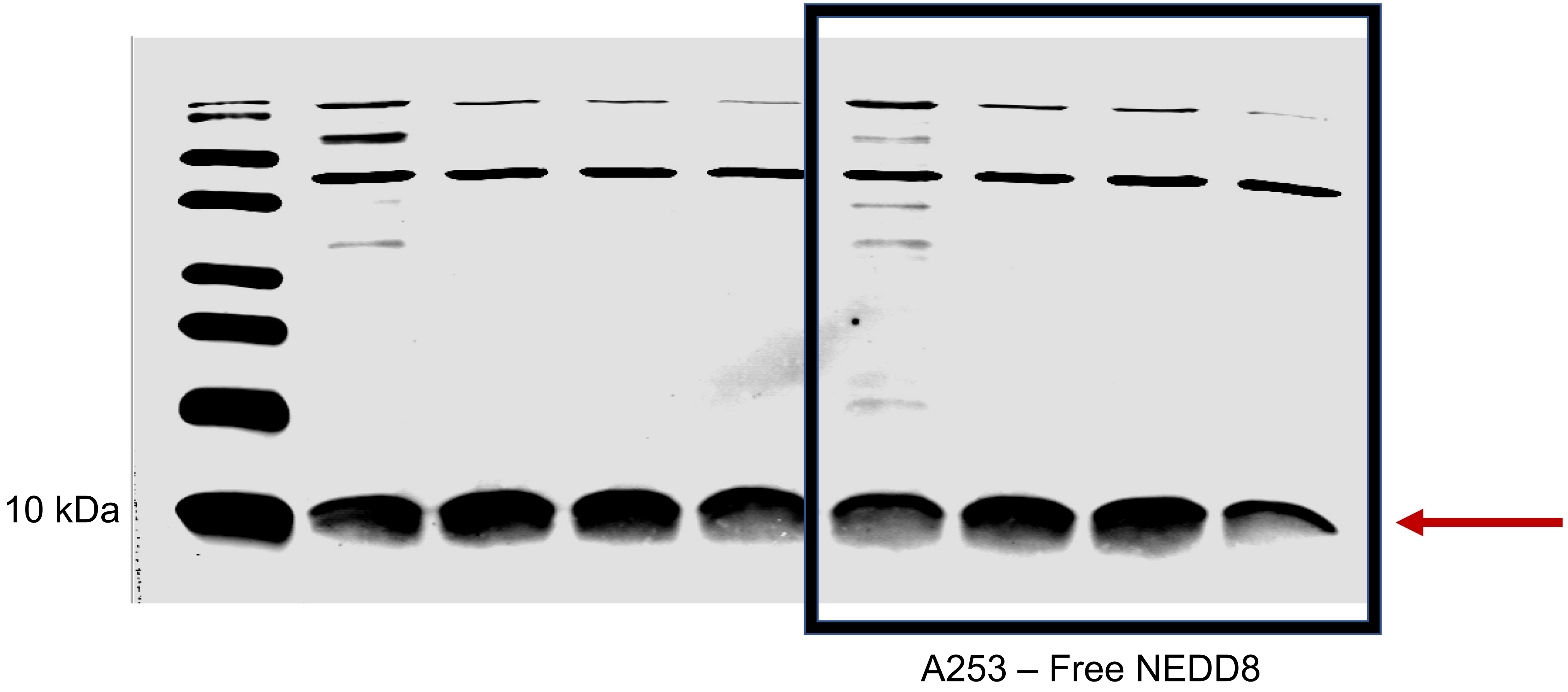

Figure 1C

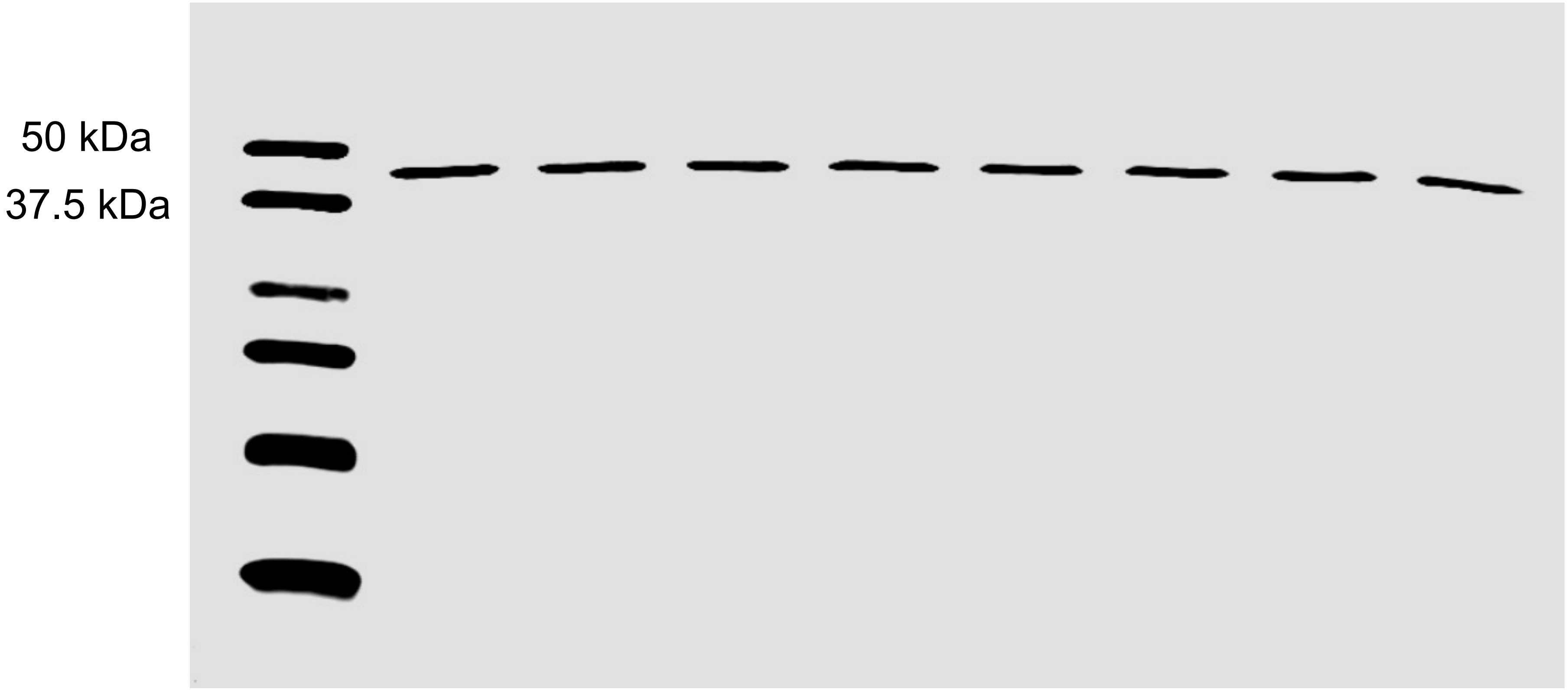

β-Actin

Figure 1C

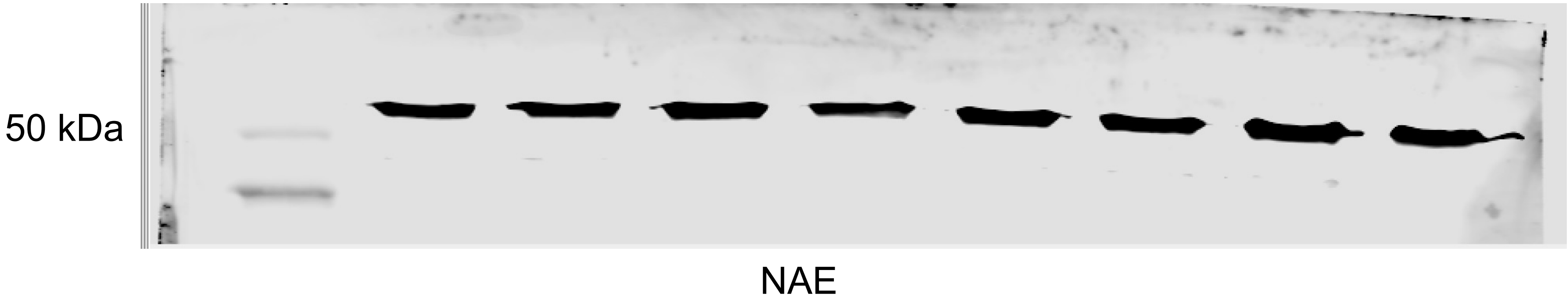

Figure 1C

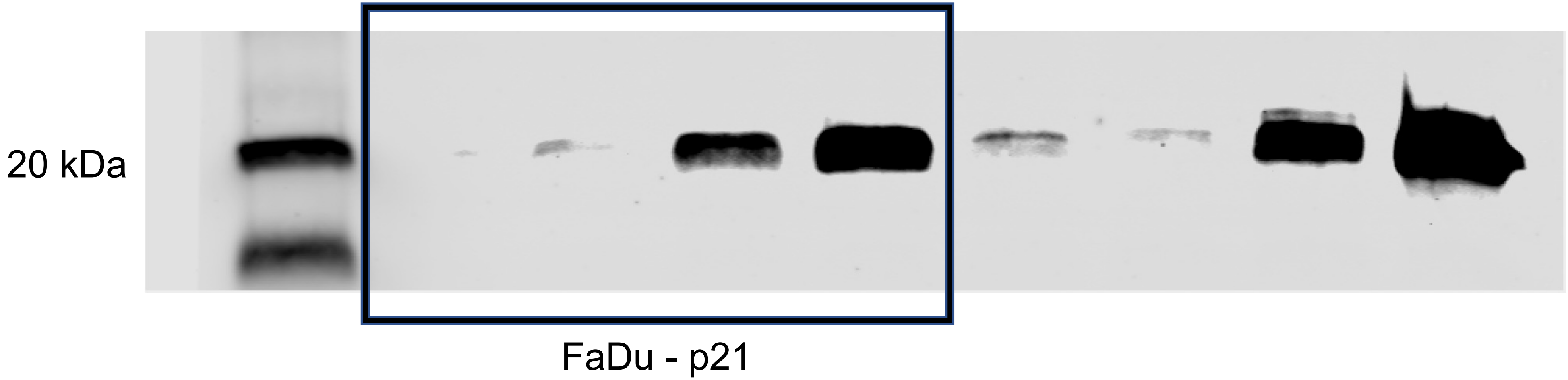

Figure 1C

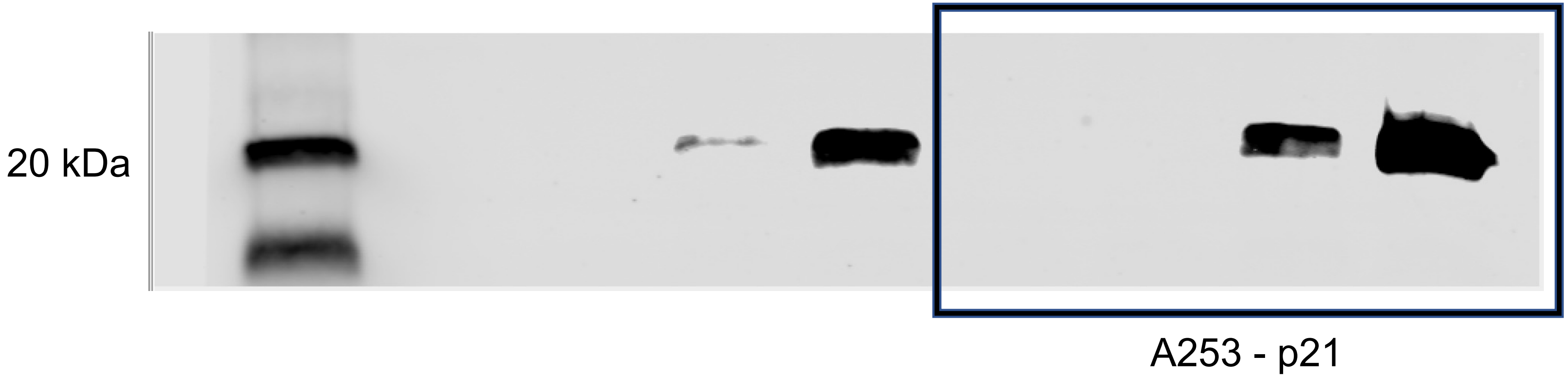

Figure 1C

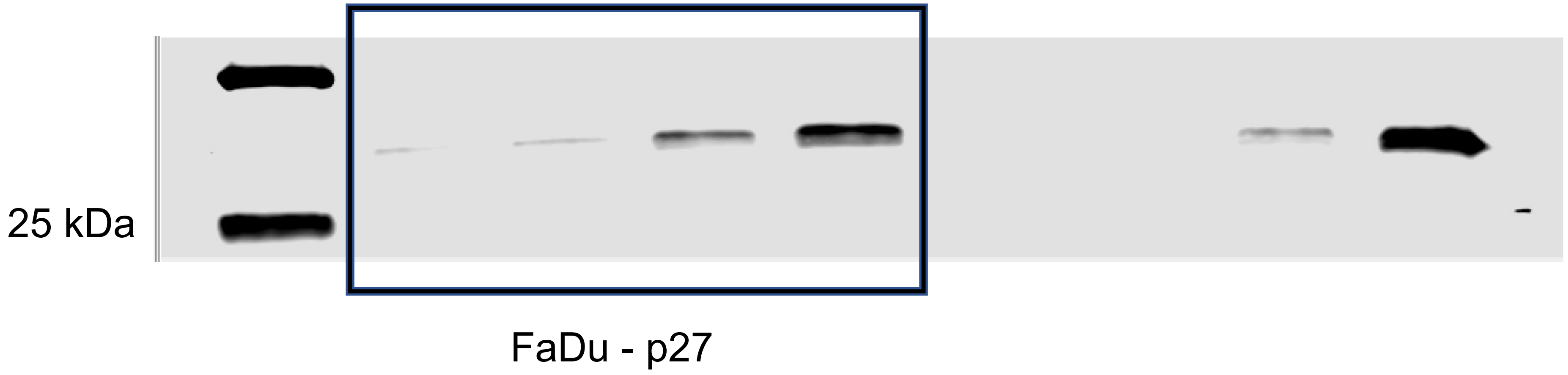

Figure 1C

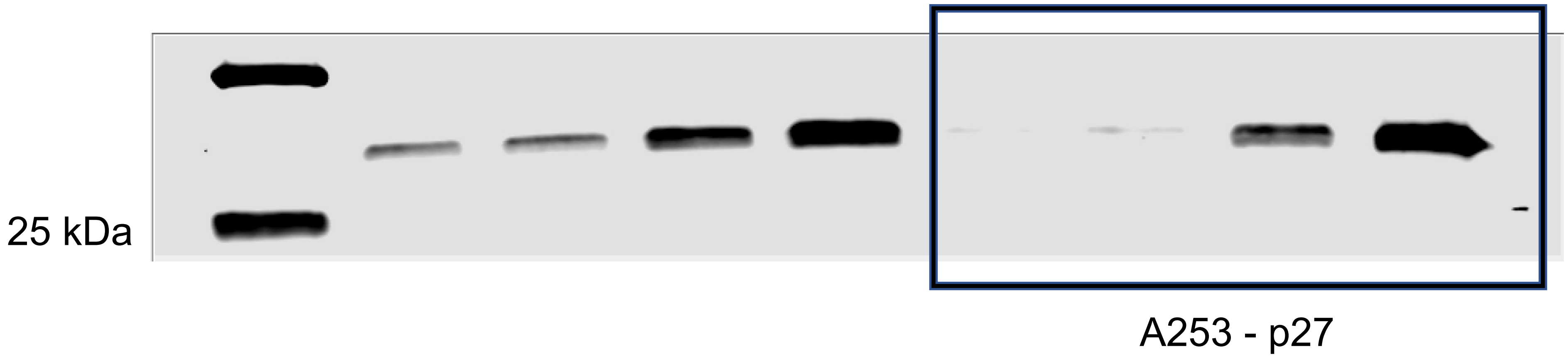

Figure 1C

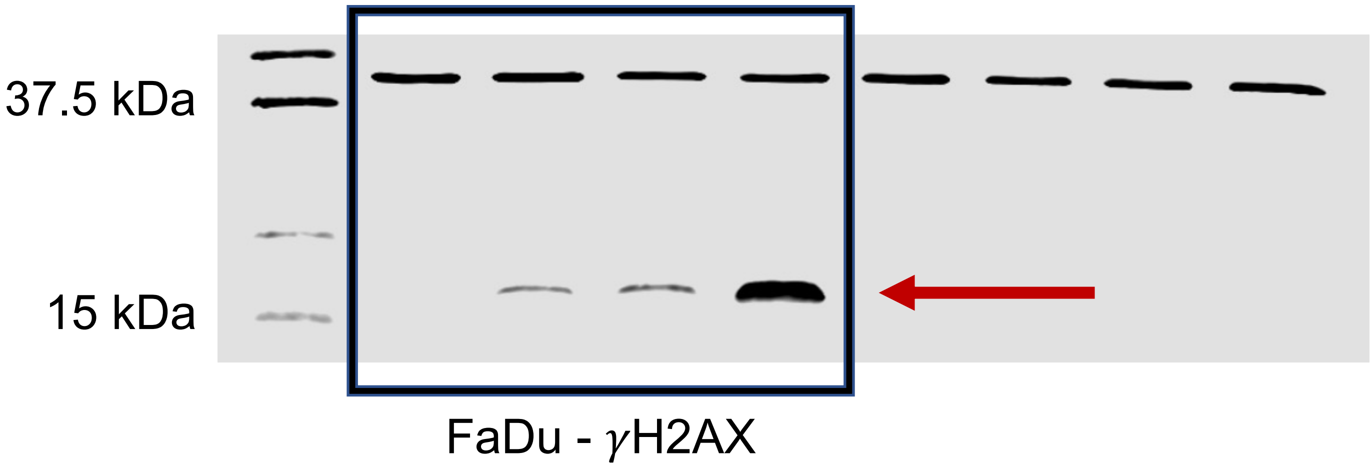

Figure 1C

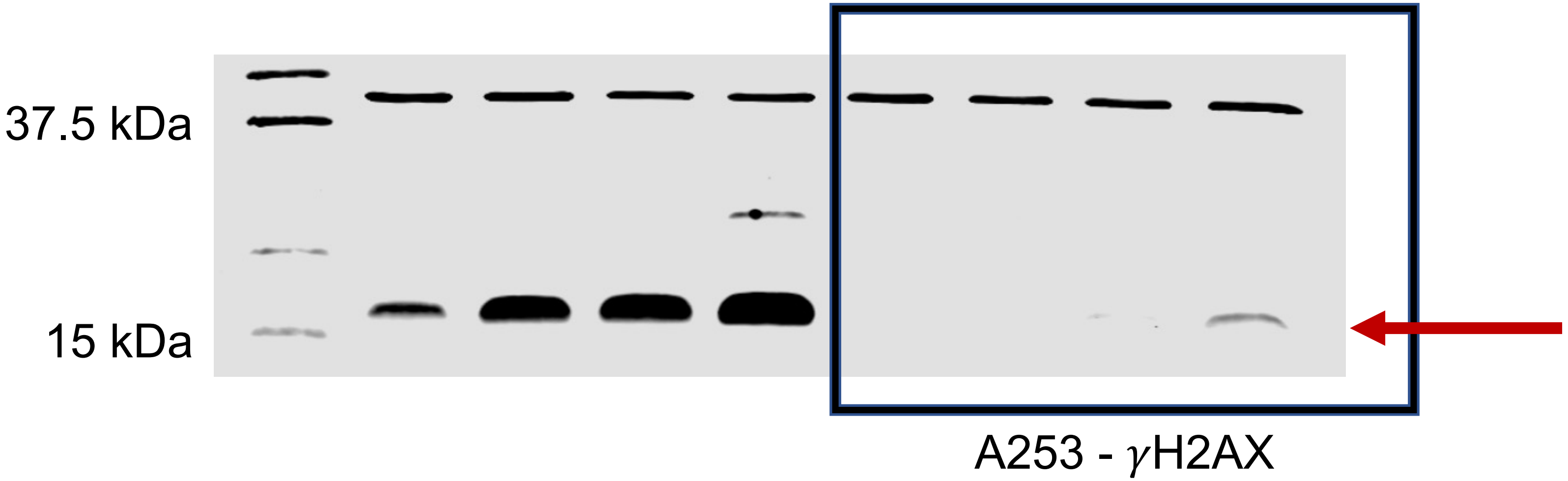

Figure 3A

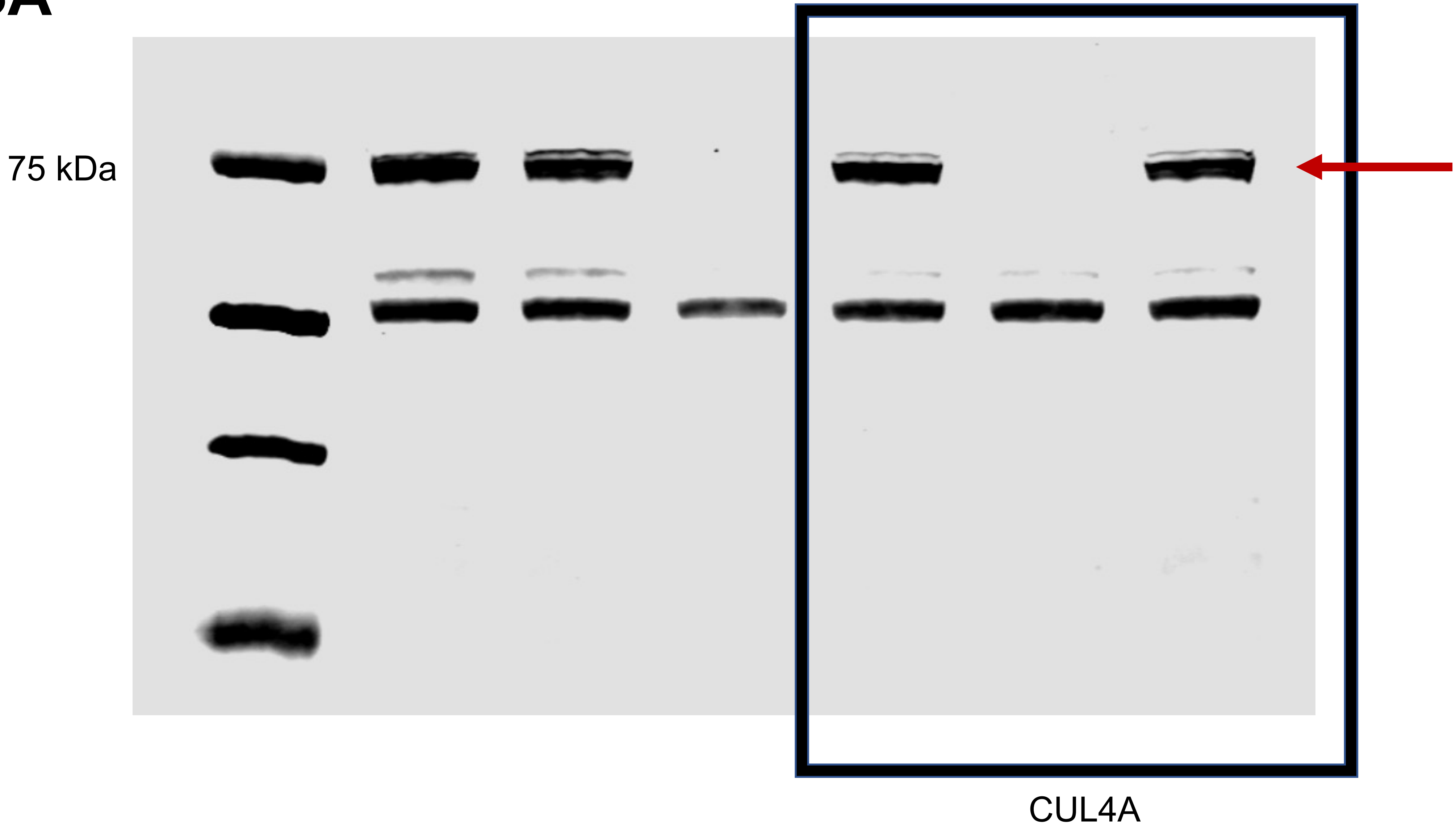

Figure 3A

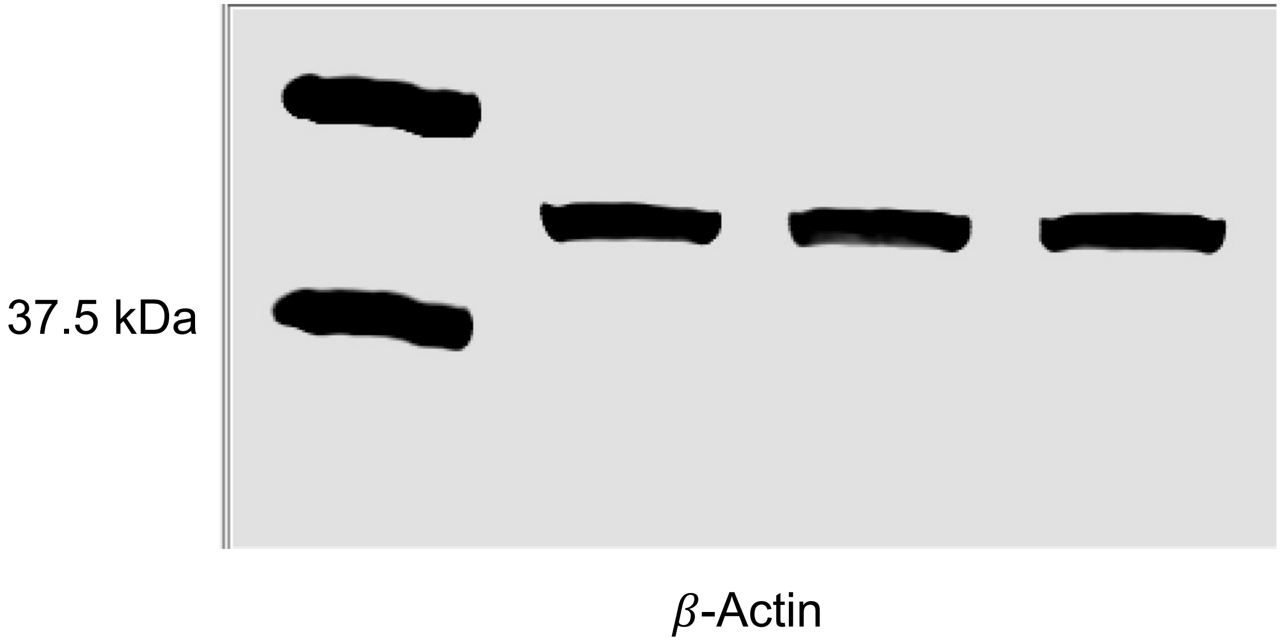

Figure 3A

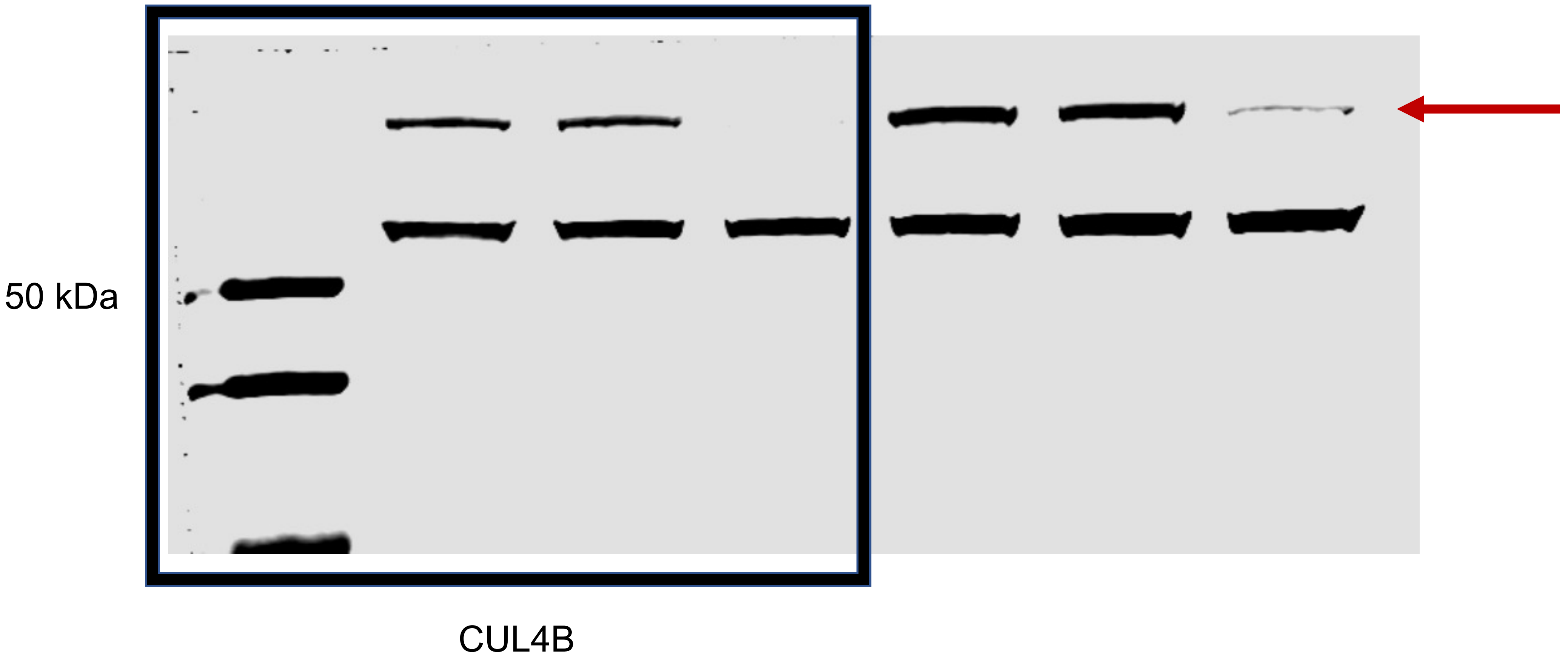

Figure 3A

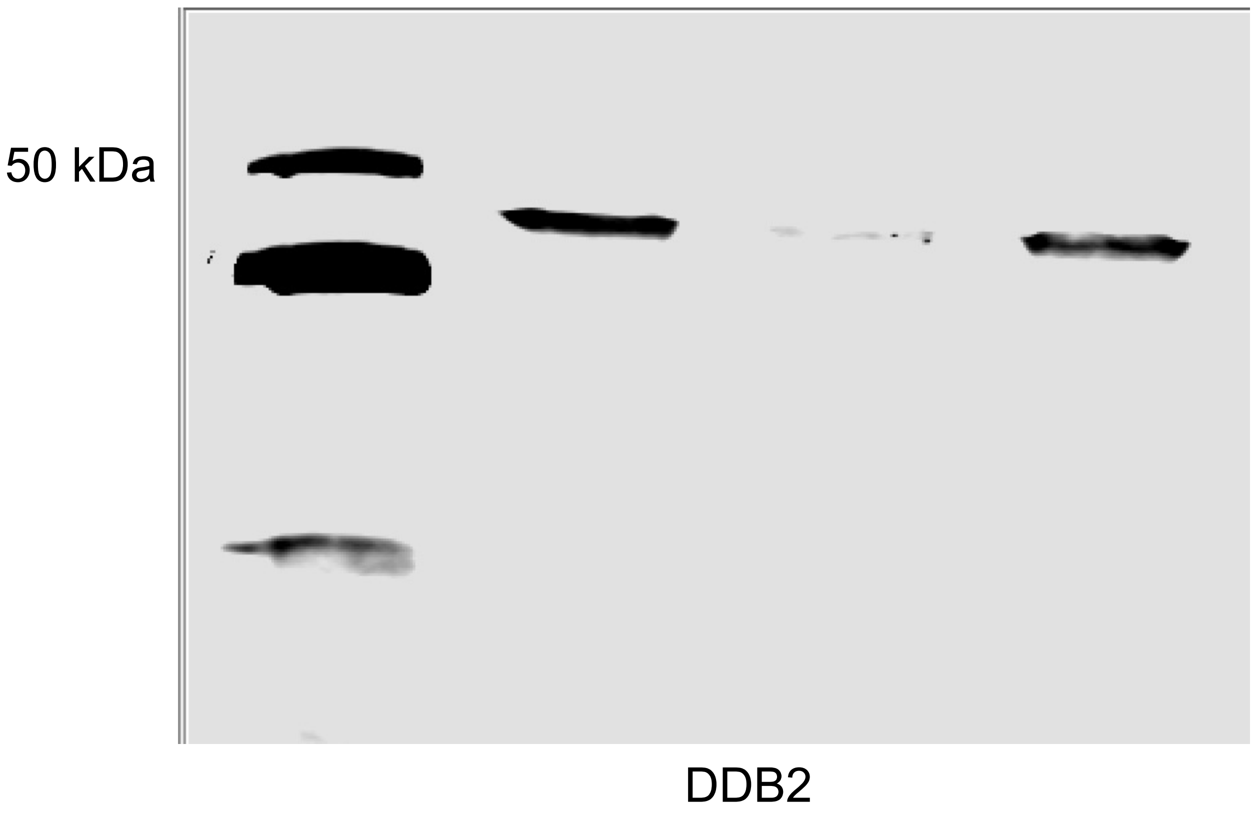

Figure 3C

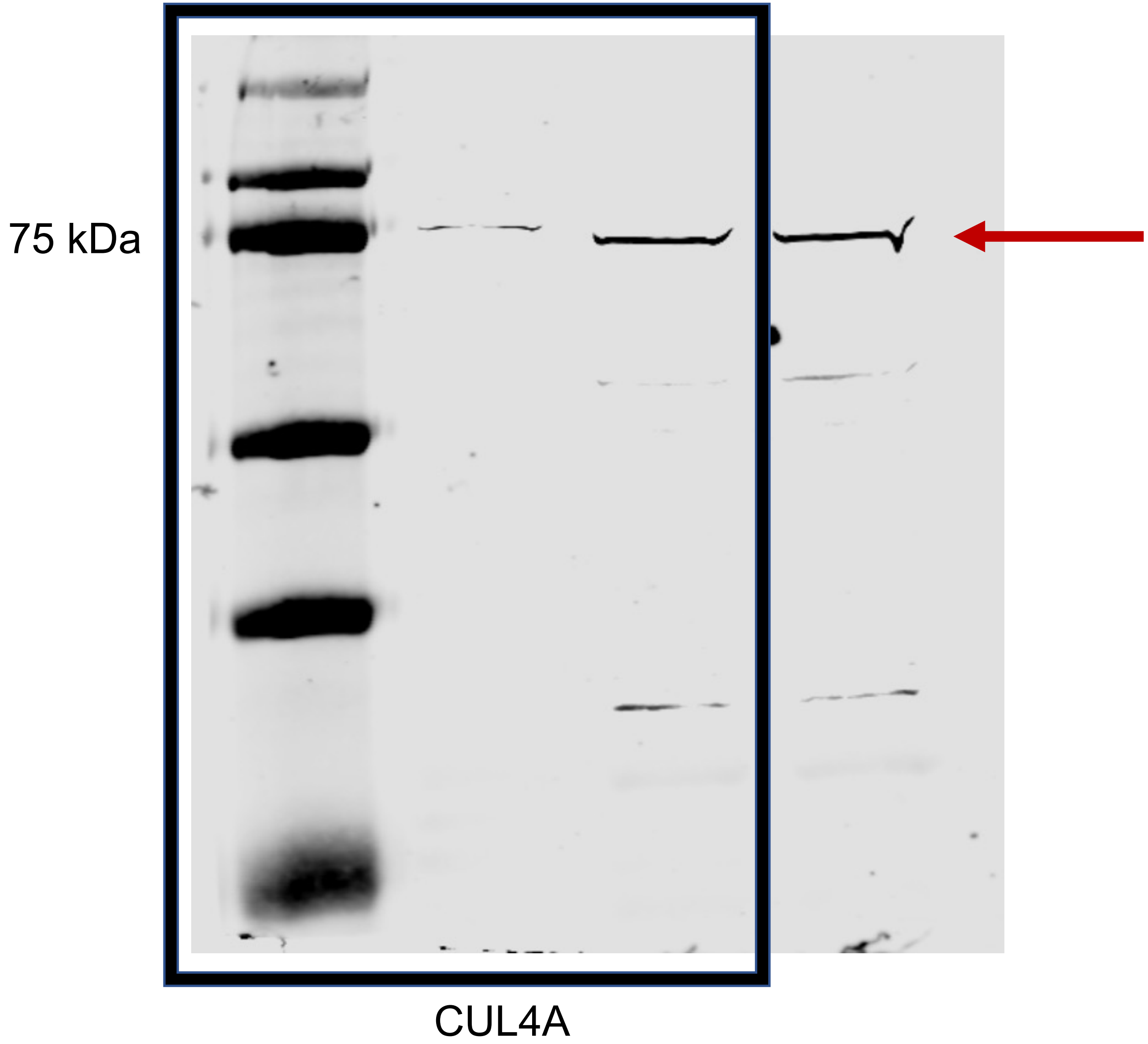

Figure 3C

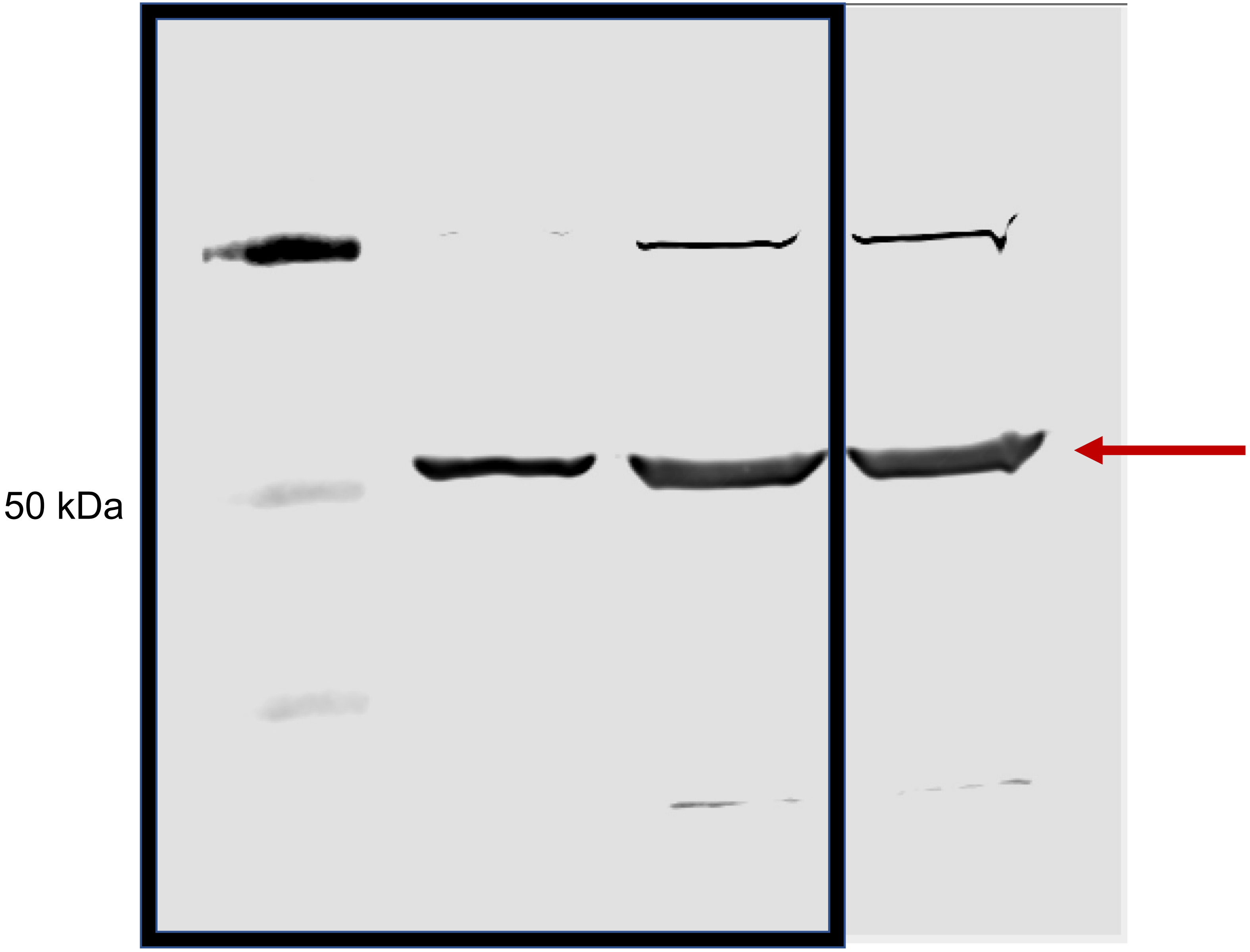

β-Tubulin

Figure 4A

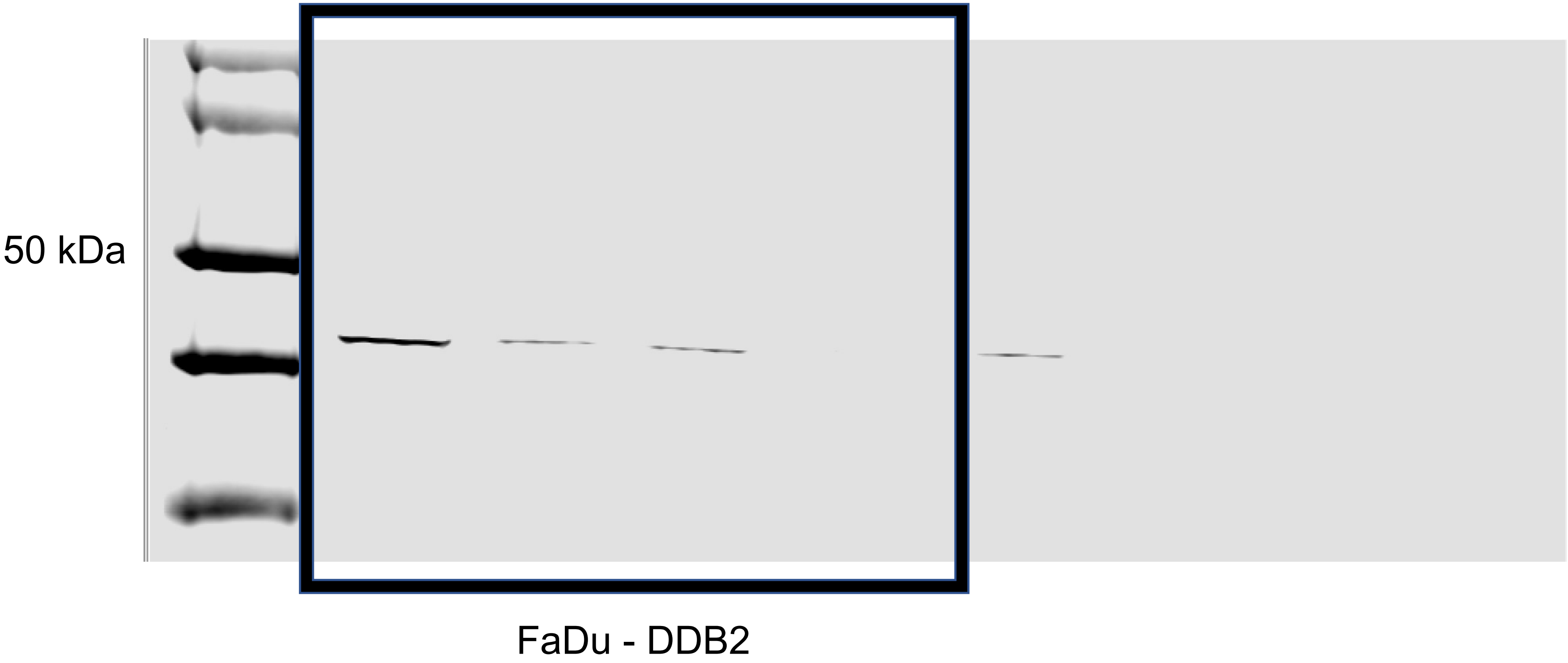

Figure 4A

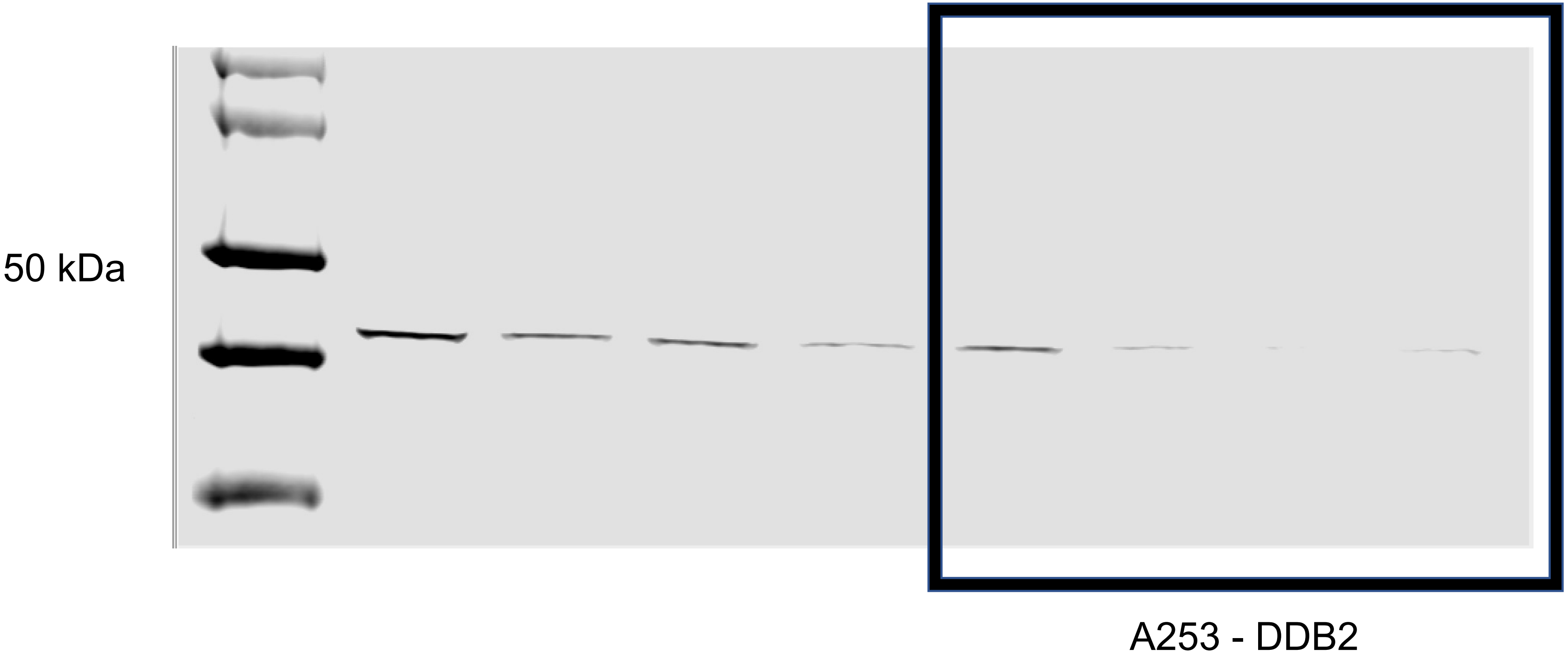

Figure 4A

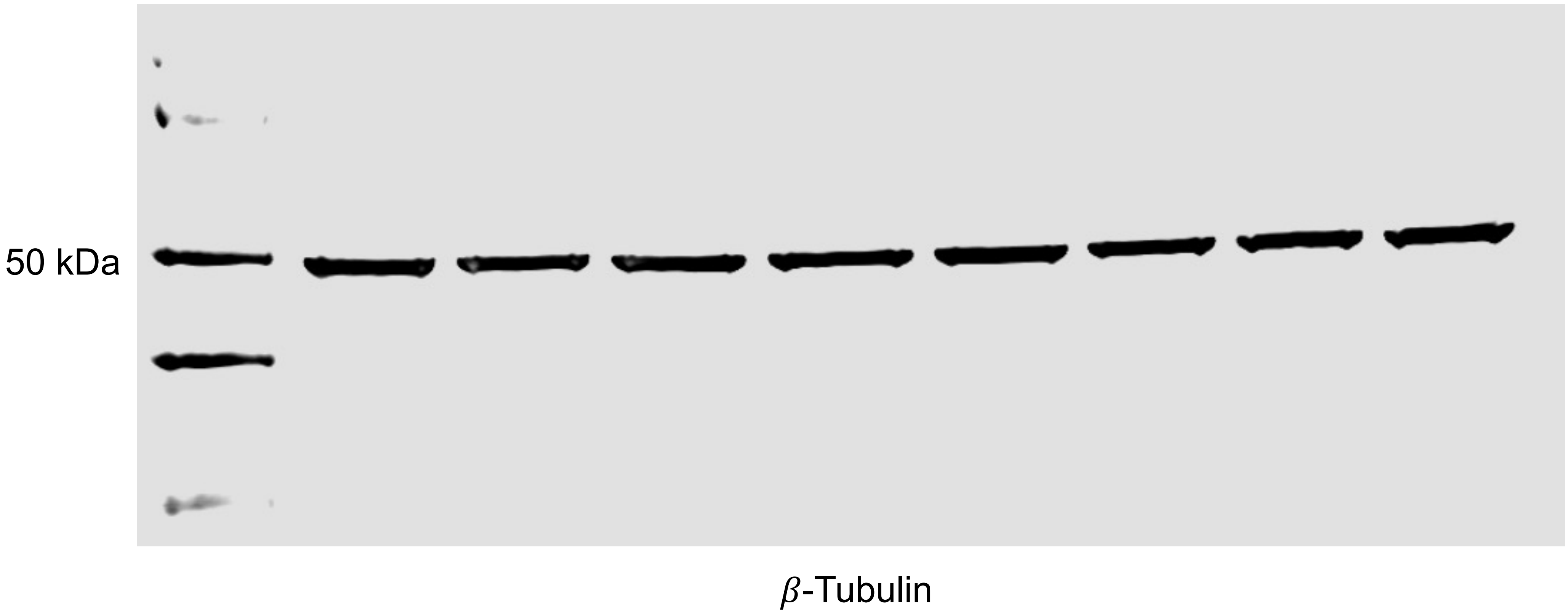

Figure 4B

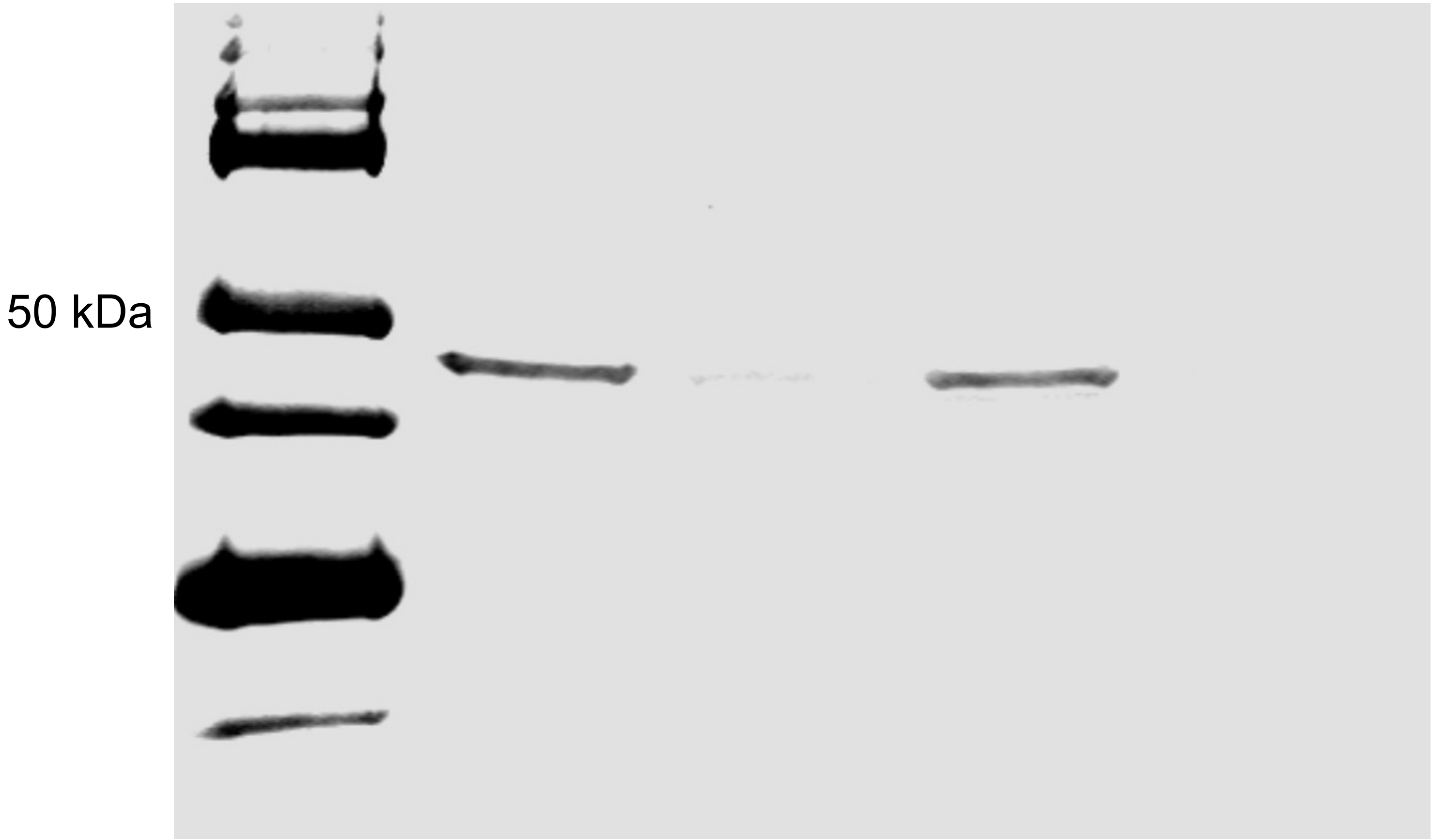

DDB2

Figure 4B

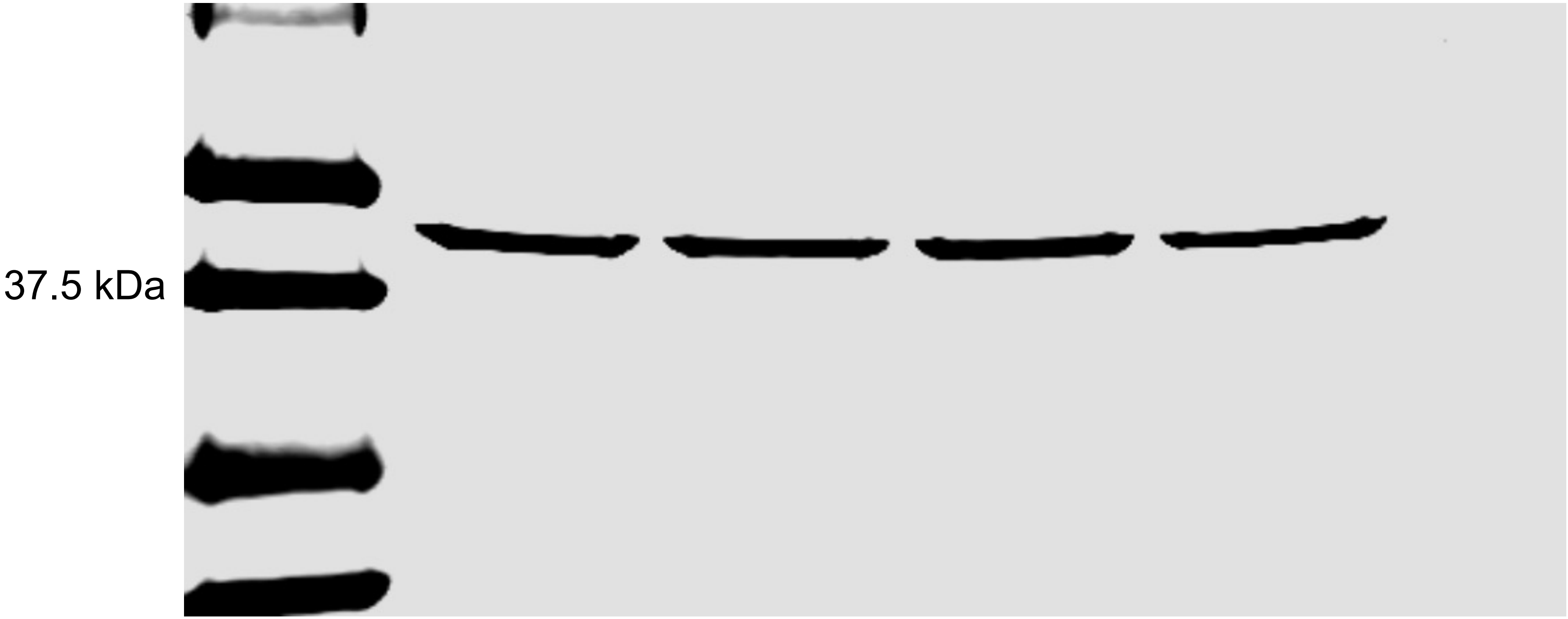

β-Actin

Figure 4C

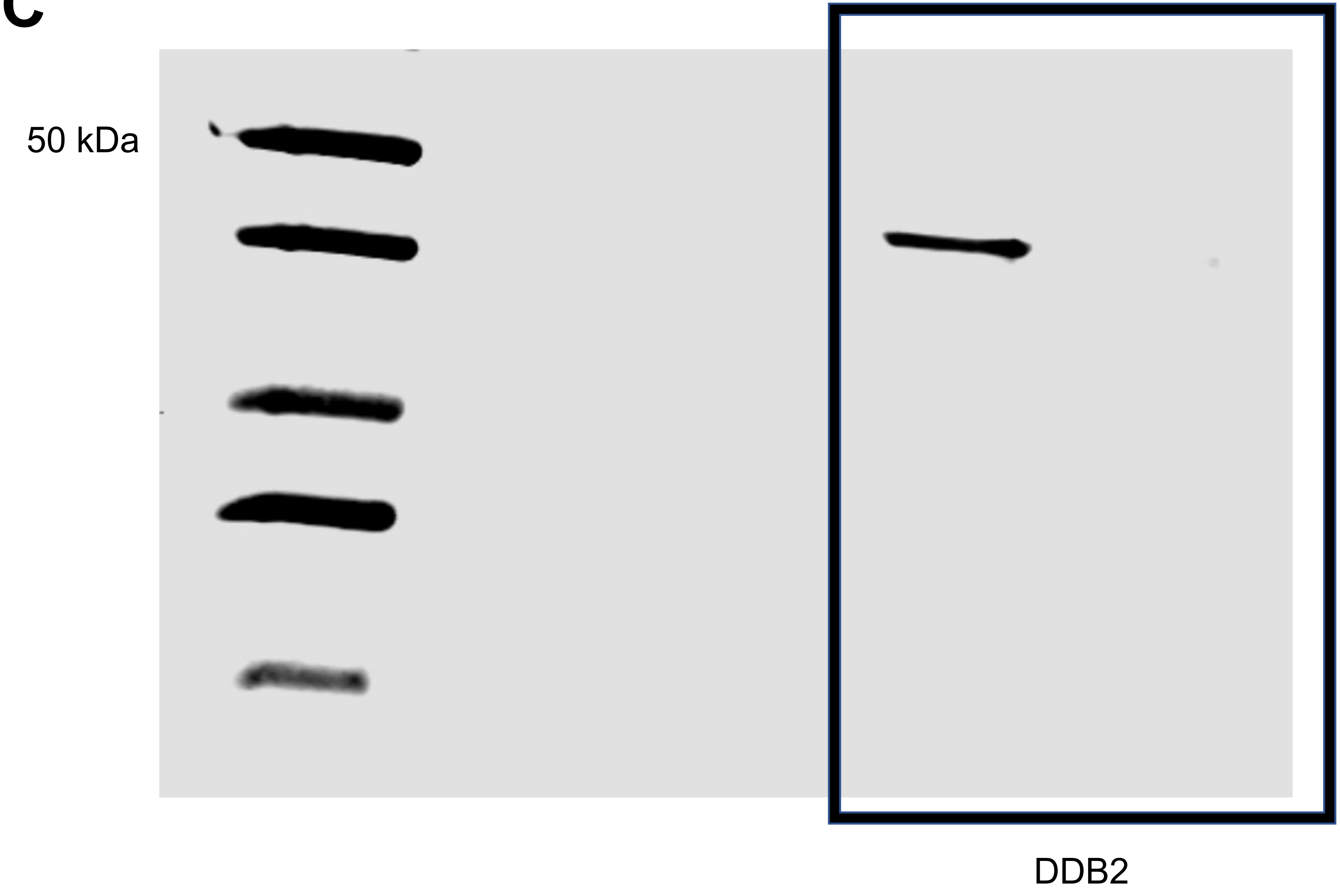

Figure 4C

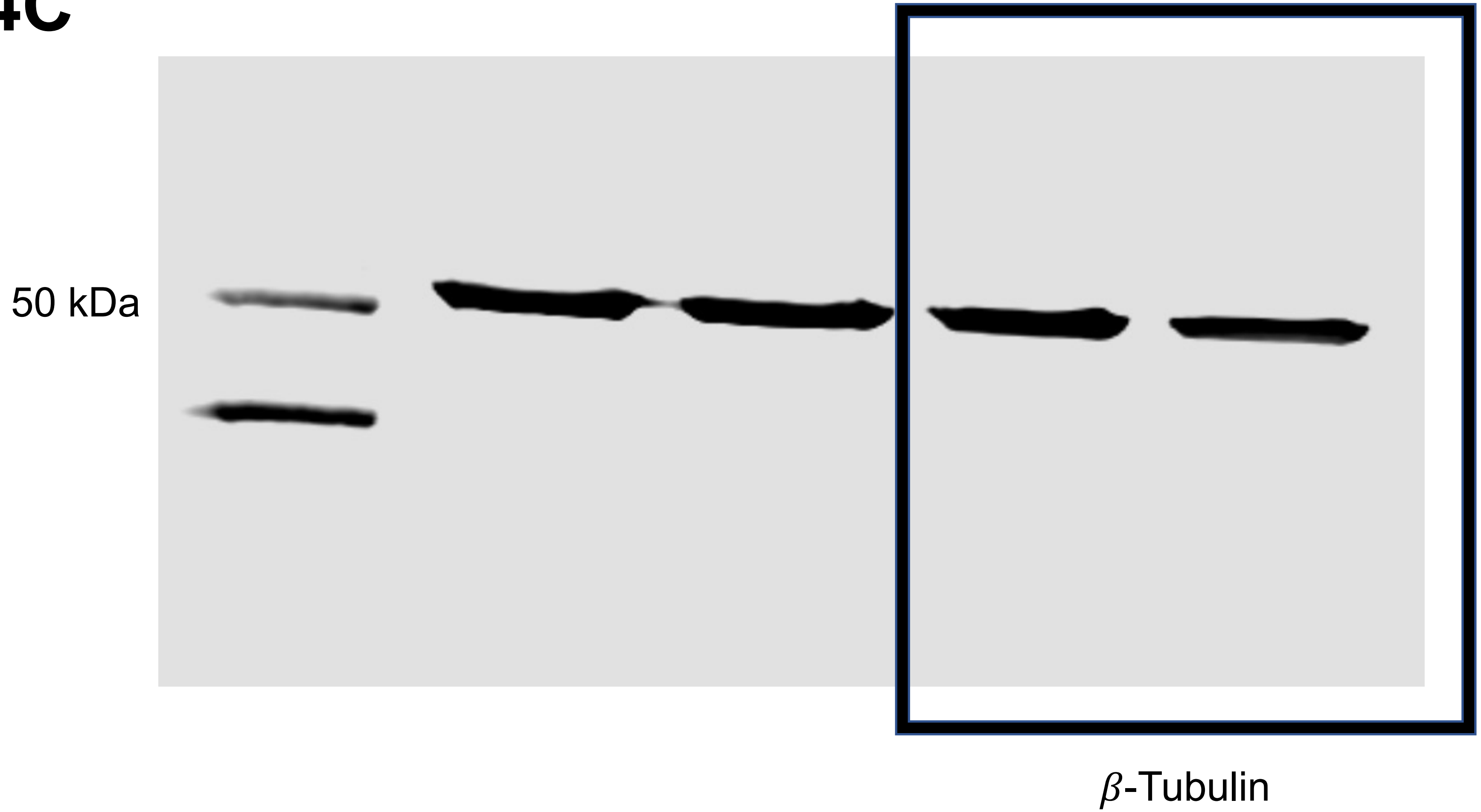

Figure 5C

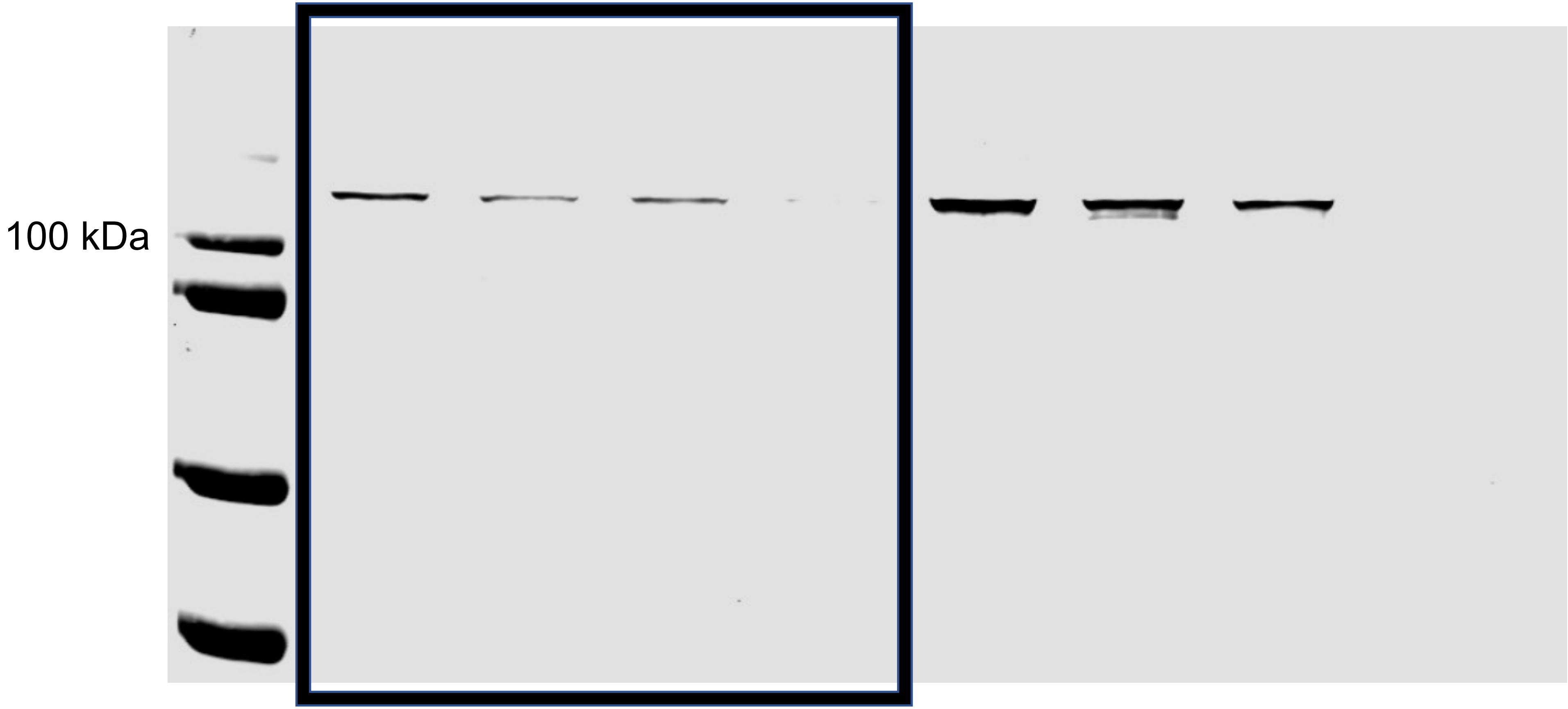

FaDu - p-Rb (Ser780)

Figure 5C

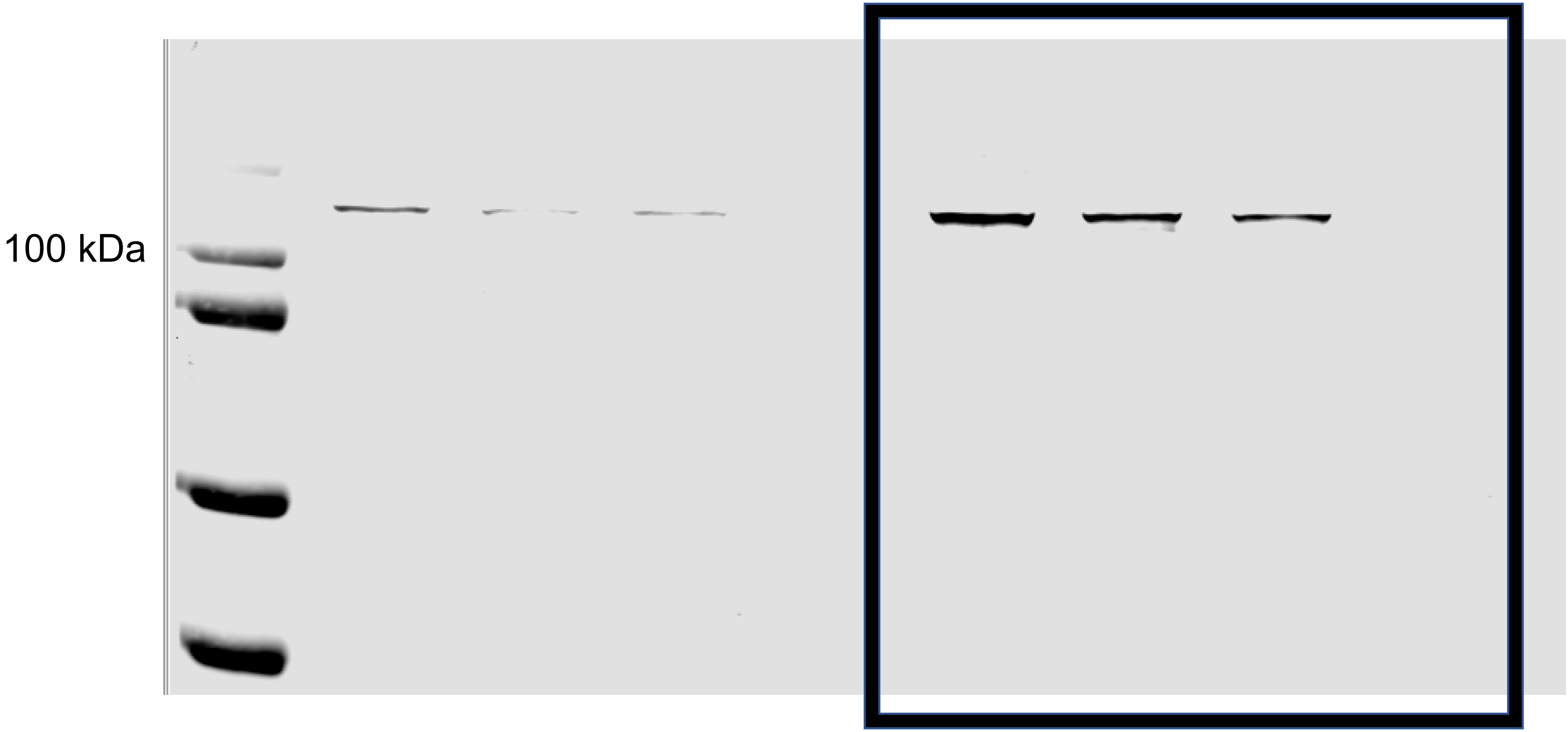

A253 - p-Rb (Ser780)

Figure 5C

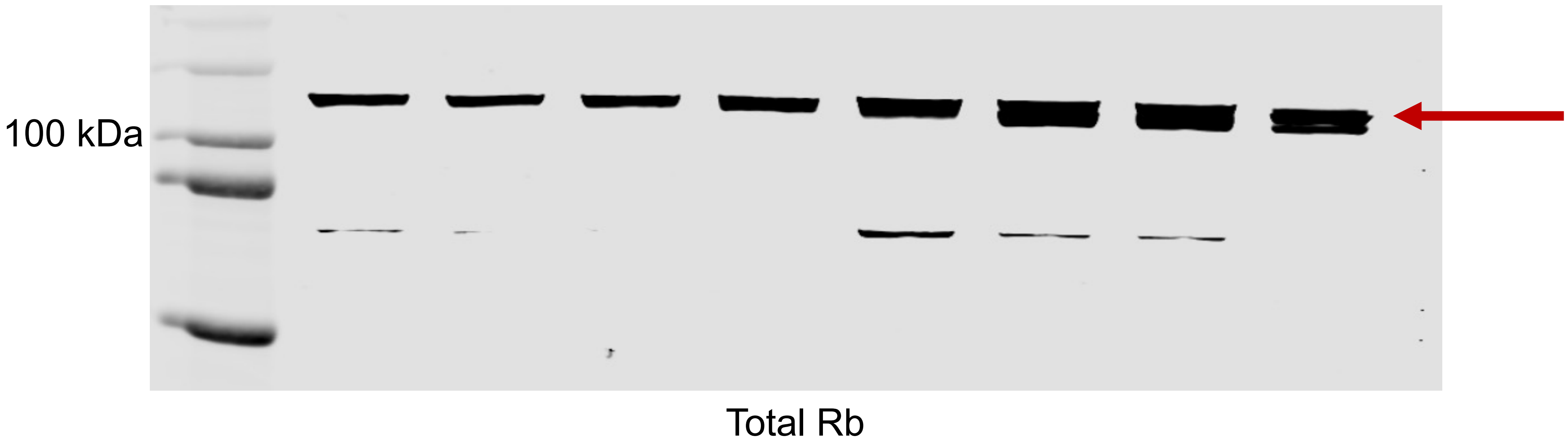

Figure 5C

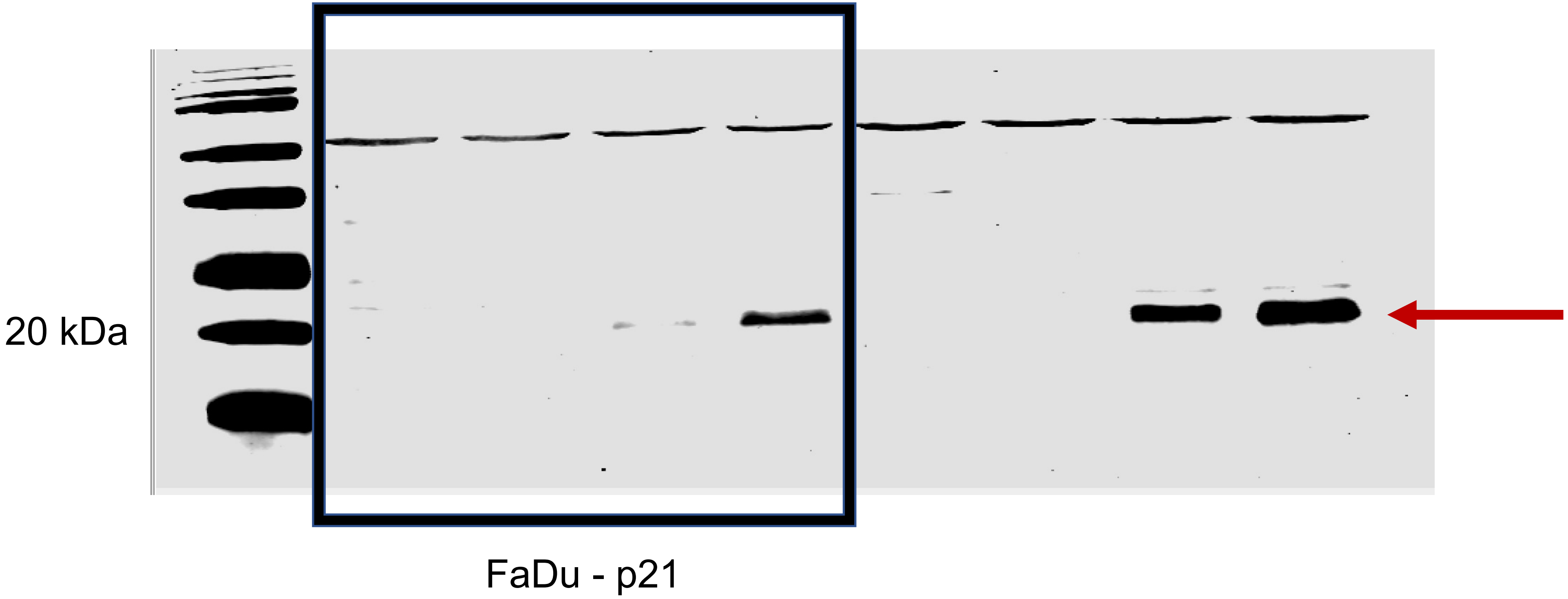

Figure 5C

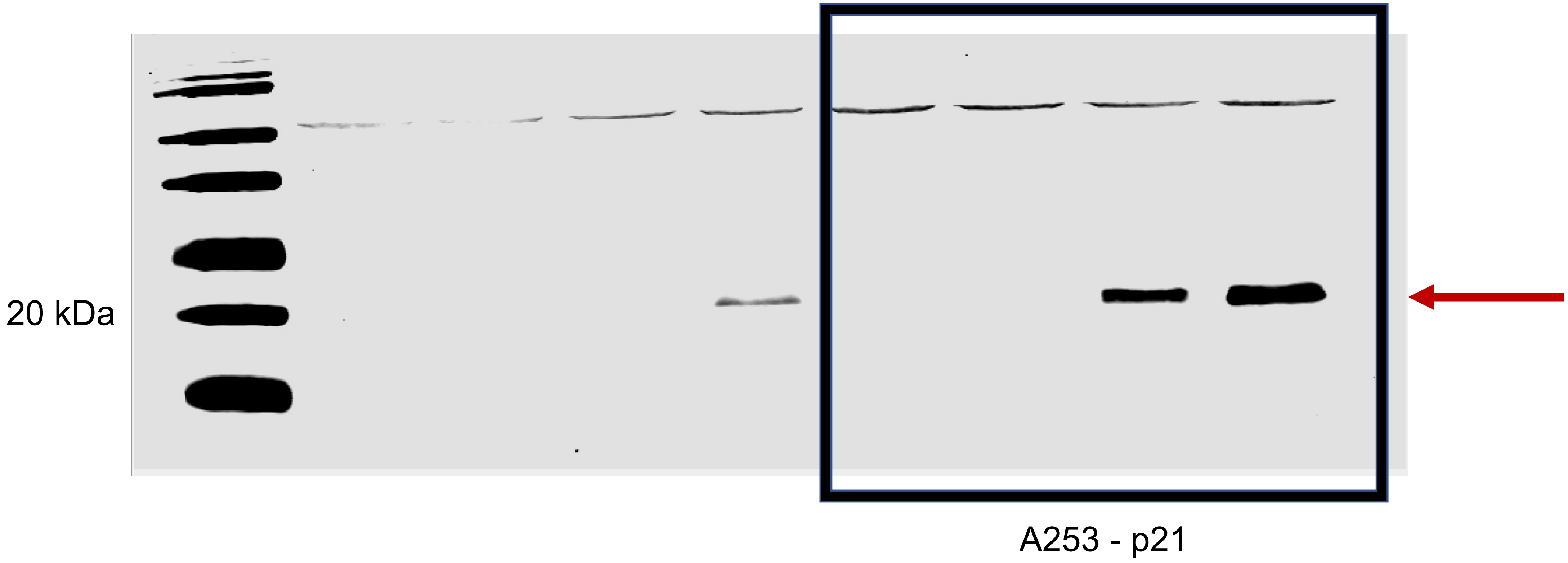

Figure 5C

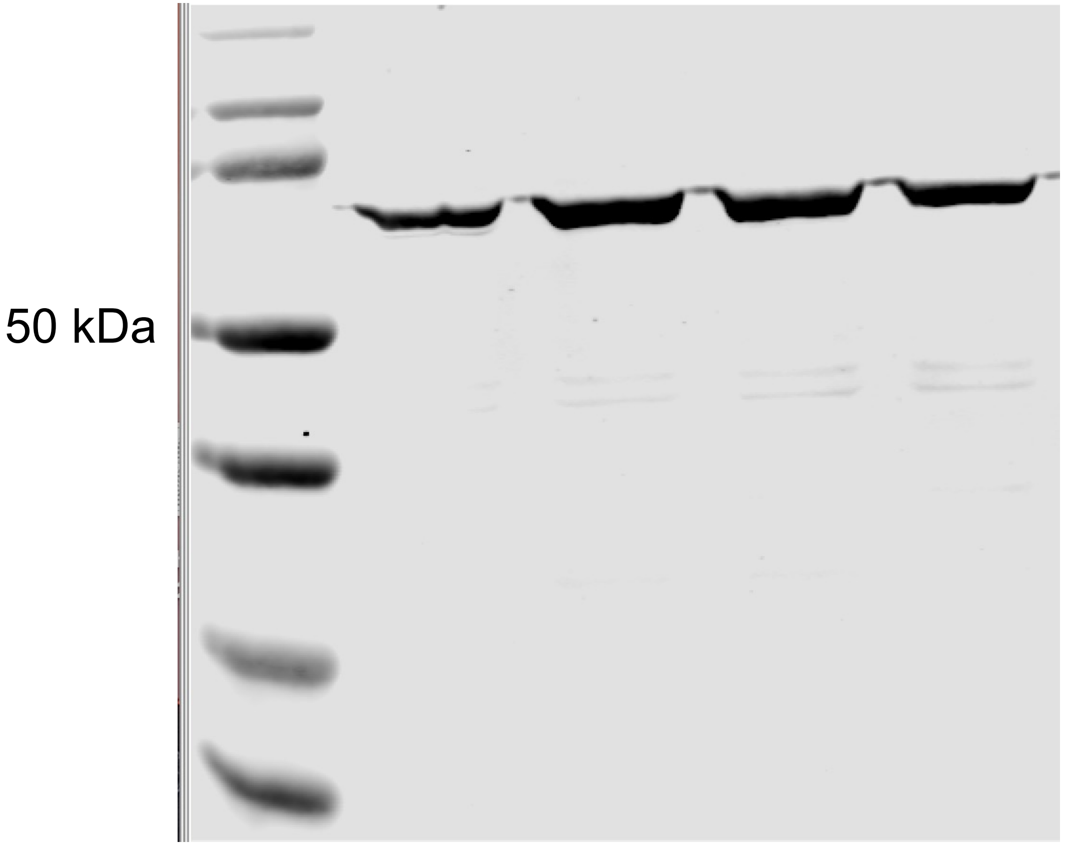

FaDu – E2F1

Figure 5C

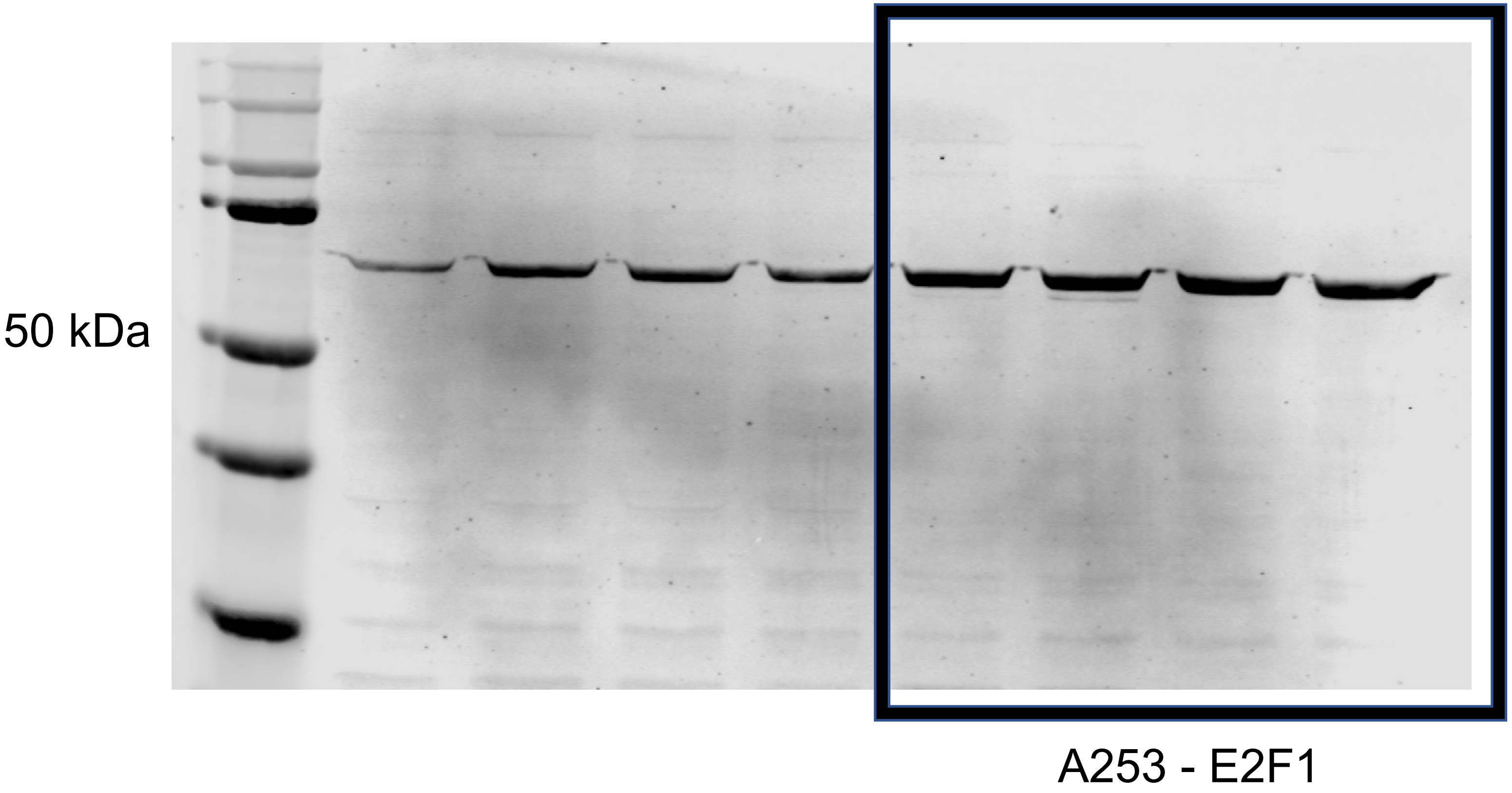

Figure 5C

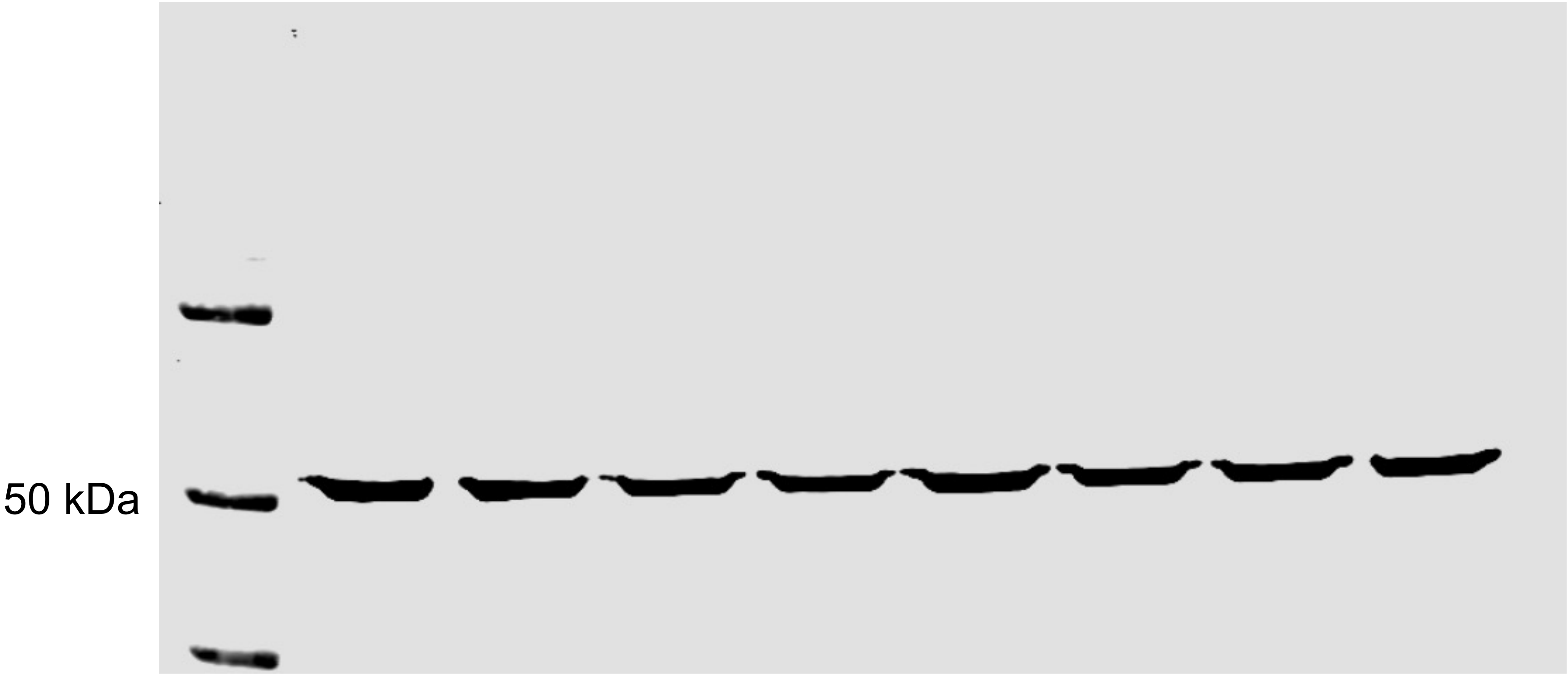

β-Tubulin

Figure 5D

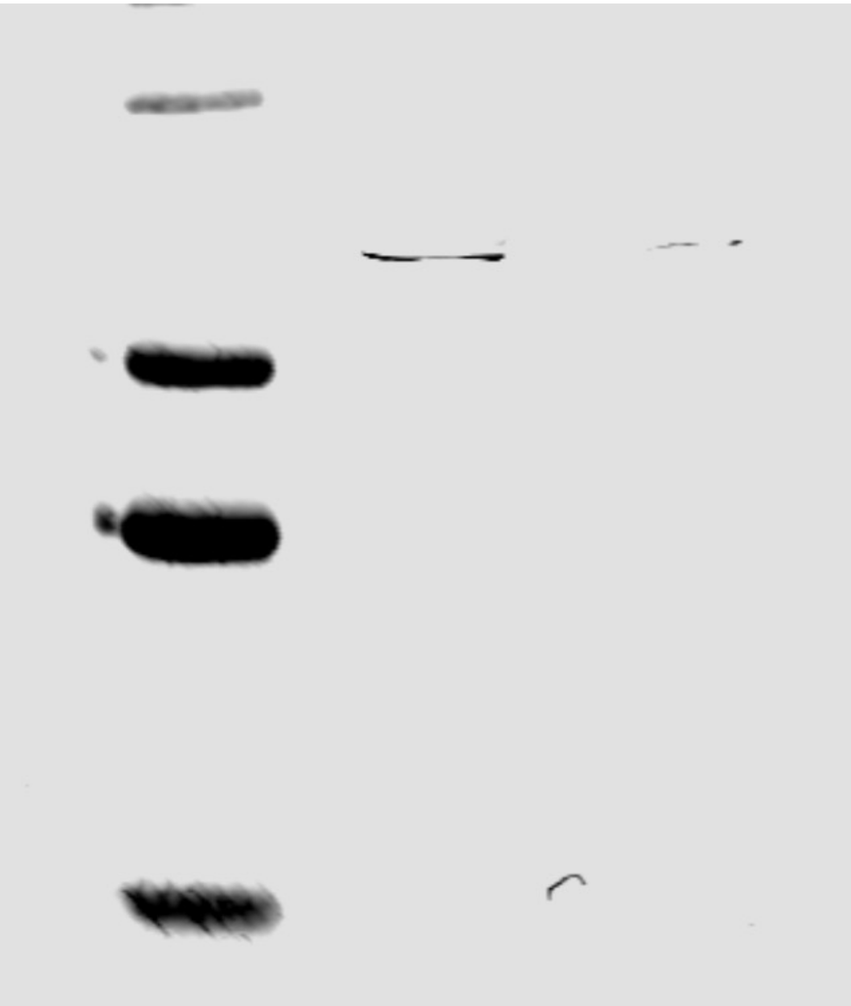

E2F1

Figure 5D

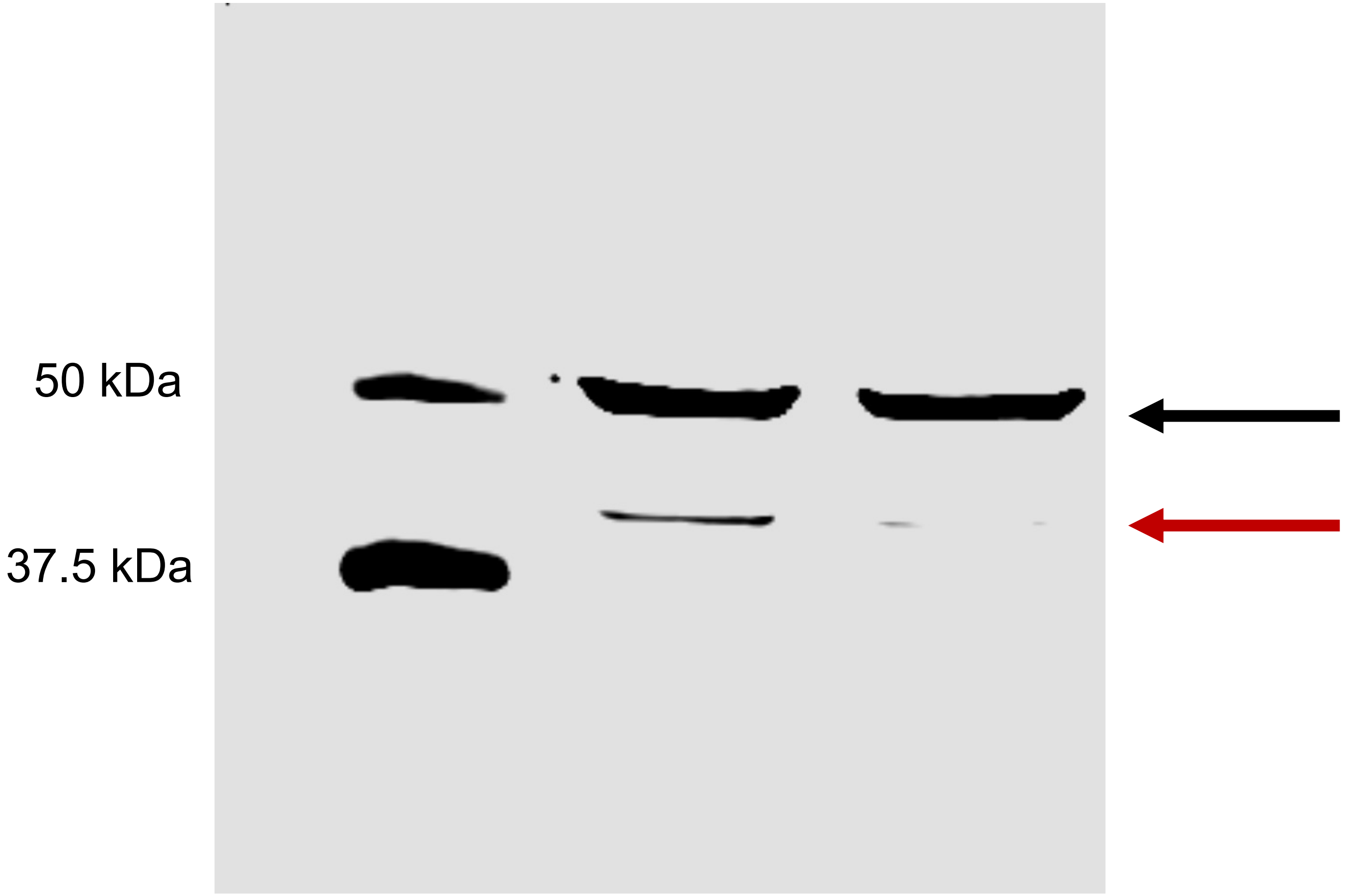

DDB2 (Red Arrow) and β-Tubulin (Black Arrow)

Supp. Figure S2

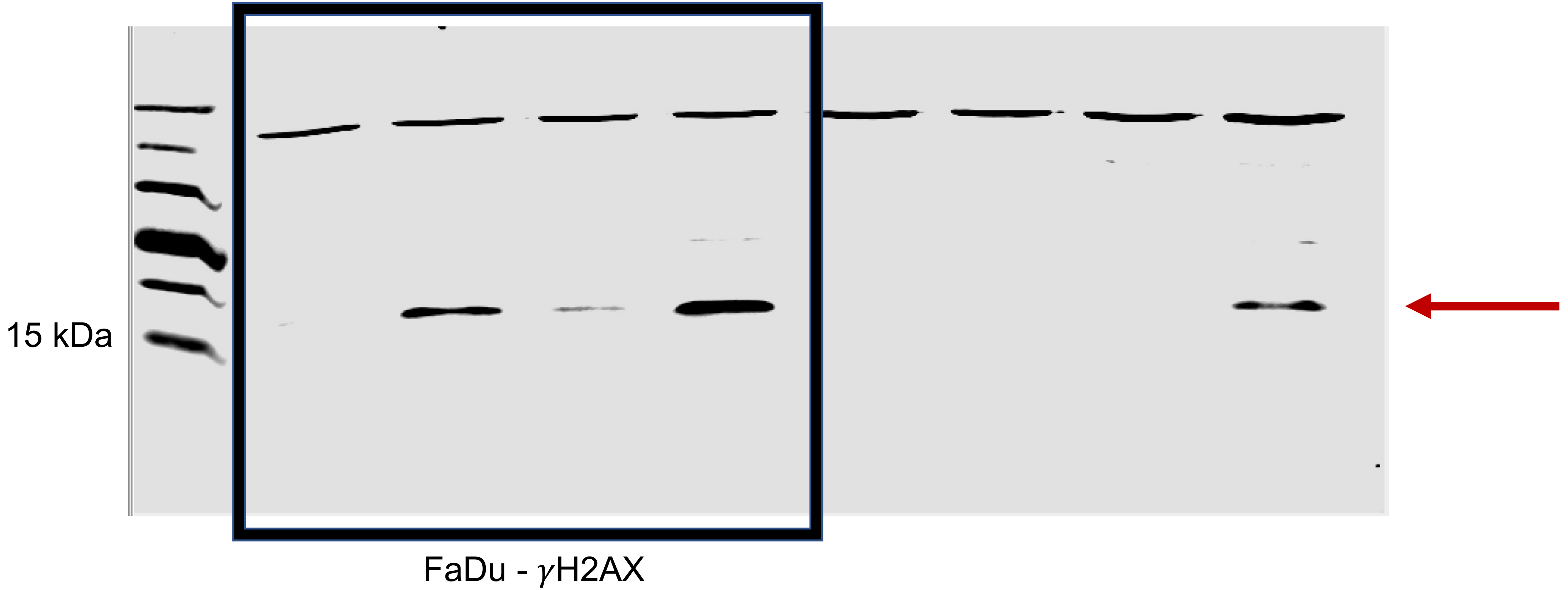

Supp. Figure S2

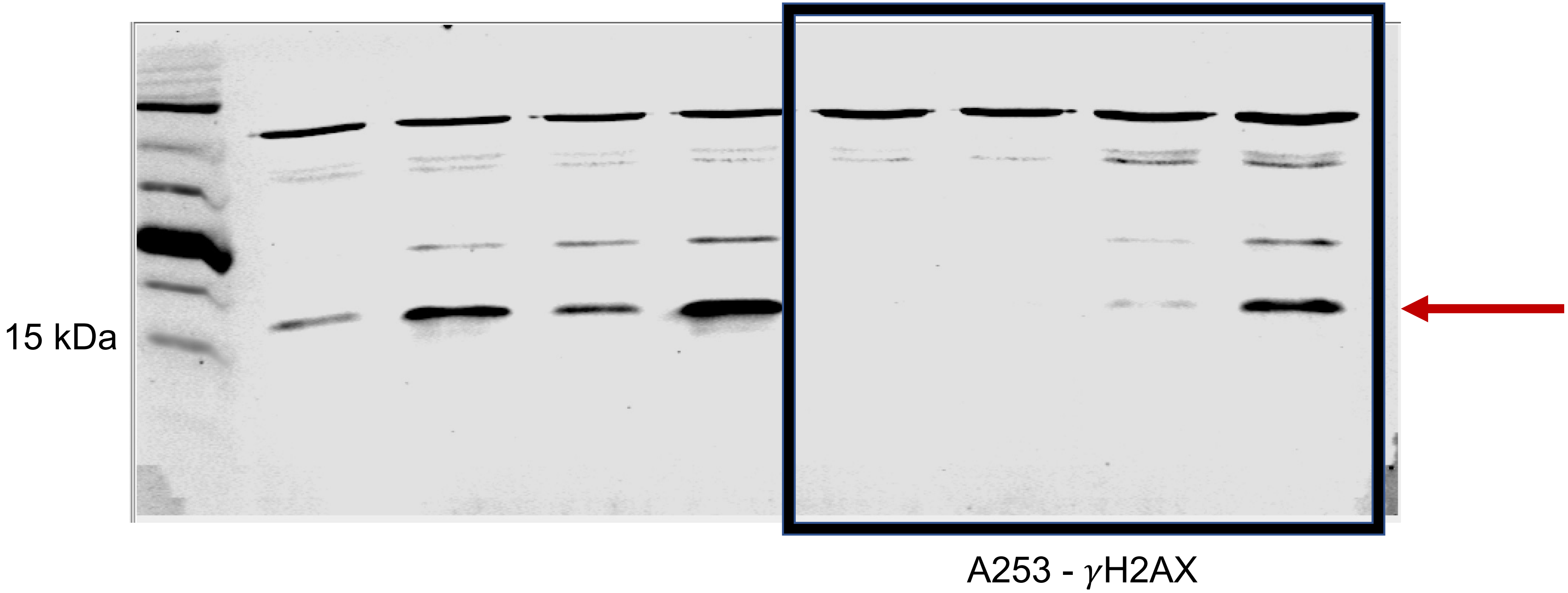

Supp. Figure S2

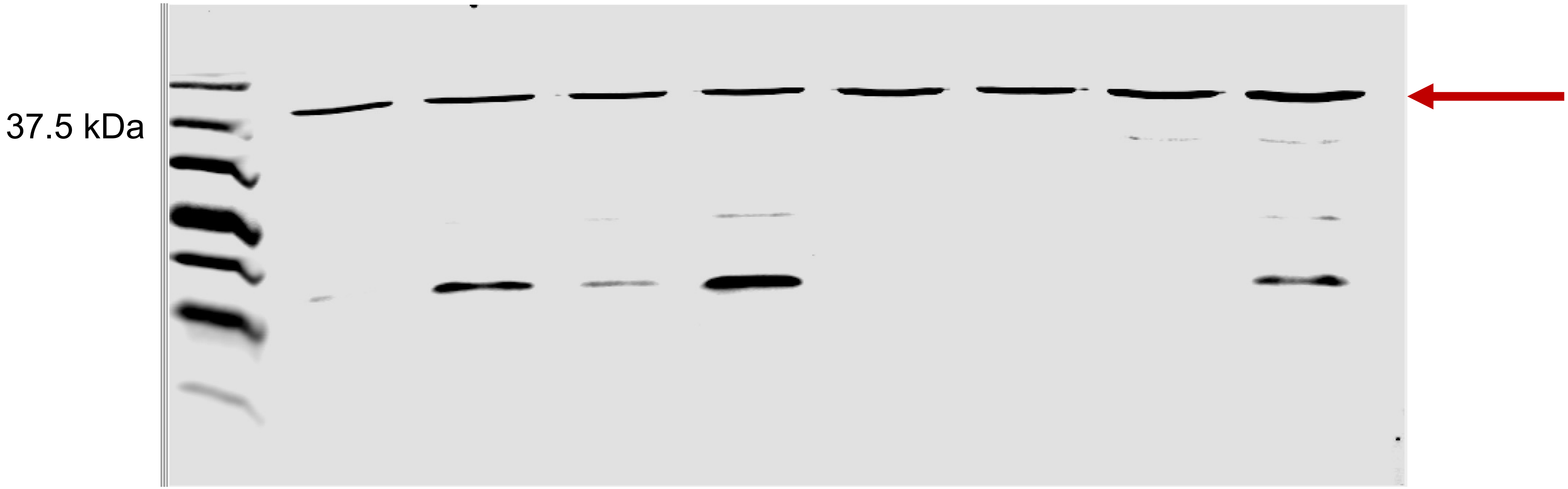

β-Actin

Supp. Figure S6

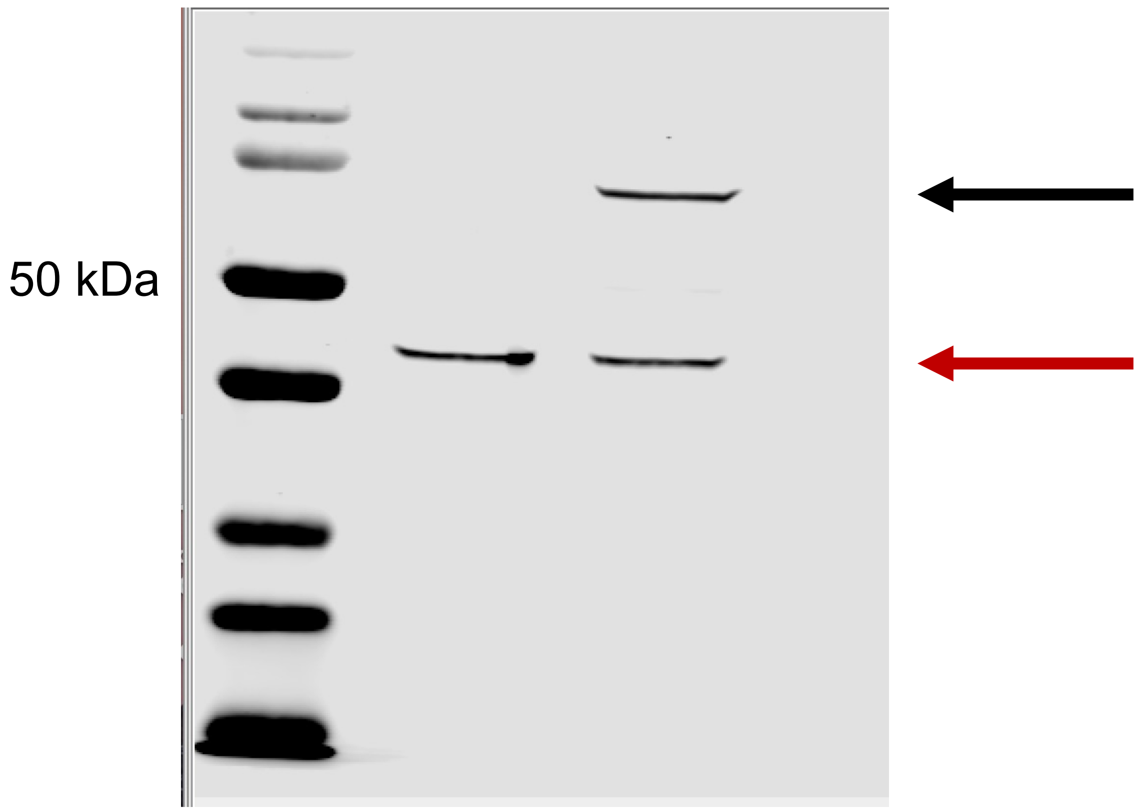

DDB2 (Red Arrow) and GFP-DDB2 (Black Arrow)

Supp. Figure S6

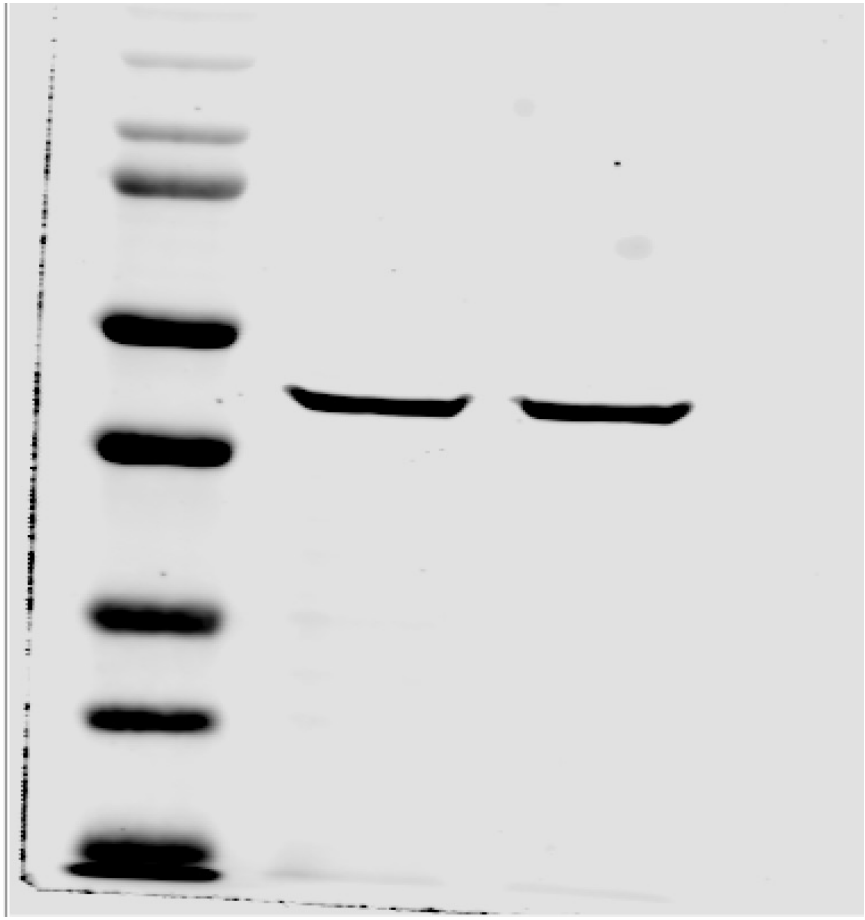

β-Actin
